# Nanomedicine for Diagnosis and Treatment of Atherosclerosis

**DOI:** 10.1002/advs.202304294

**Published:** 2023-10-28

**Authors:** Jingyun Cheng, Hui Huang, Yu Chen, Rong Wu

**Affiliations:** ^1^ Department of Ultrasound Shanghai General Hospital Shanghai Jiao Tong University School of Medicine Shanghai 200080 P. R. China; ^2^ Materdicine Lab School of Life Sciences Shanghai University Shanghai 200444 P. R. China; ^3^ Oujiang Laboratory (Zhejiang Lab for Regenerative Medicine, Vision and Brain Health) Wenzhou Institute of Shanghai University Wenzhou Zhejiang 325088 P. R. China

**Keywords:** atherosclerosis, biomaterials, diagnosis, nanomedicine, theranostic, therapy

## Abstract

With the changing disease spectrum, atherosclerosis has become increasingly prevalent worldwide and the associated diseases have emerged as the leading cause of death. Due to their fascinating physical, chemical, and biological characteristics, nanomaterials are regarded as a promising tool to tackle enormous challenges in medicine. The emerging discipline of nanomedicine has filled a huge application gap in the atherosclerotic field, ushering a new generation of diagnosis and treatment strategies. Herein, based on the essential pathogenic contributors of atherogenesis, as well as the distinct composition/structural characteristics, synthesis strategies, and surface design of nanoplatforms, the three major application branches (nanodiagnosis, nanotherapy, and nanotheranostic) of nanomedicine in atherosclerosis are elaborated. Then, state‐of‐art studies containing a sequence of representative and significant achievements are summarized in detail with an emphasis on the intrinsic interaction/relationship between nanomedicines and atherosclerosis. Particularly, attention is paid to the biosafety of nanomedicines, which aims to pave the way for future clinical translation of this burgeoning field. Finally, this comprehensive review is concluded by proposing unresolved key scientific issues and sharing the vision and expectation for the future, fully elucidating the closed loop from atherogenesis to the application paradigm of nanomedicines for advancing the early achievement of clinical applications.

## Graphical abstract

This review offers an up‐to‐date standpoint on advanced nanomaterials in atherosclerotic diagnostic imaging and/or therapy, biosafety, challenges, and prospects based on the pathophysiology and optional animal models of atherosclerosis and the rational design concepts of nanoplatforms.

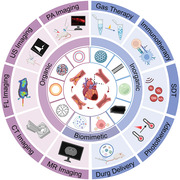

## 1 Introduction

Cardiovascular and cerebrovascular diseases are sweeping the globe and becoming the primary reason of mortality and morbidity worldwide.^[^ ^1^ ^]^ Atherosclerosis, the leading cause of cardiovascular and cerebrovascular diseases, is a chronic, systemic, and inflammatory disease, mainly affecting large and medium‐sized arteries.^[^ ^2^ ^]^ It is particularly common in arteries with low endothelial shear stress or disturbed blood flow, such as the site of arterial bends or bifurcations.^[^ ^3^ ^]^ The adverse consequences usually depend on the site of occurrence, such as the coronary arteries (coronary artery syndrome), carotid or intracranial arteries (stroke, transient ischemic attack), renal arteries (renovascular hypertension or renal dysfunction), and peripheral arteries (cold extremities or claudication).^[^ ^4^ ^]^ The common risk factors include hyperlipidemia, hypertension, smoking, diabetes, etc.^[^ ^5^ ^]^ Atherosclerosis is characterized by high incidence, disability as well as mortality rates at present, and changes in modern diet and exercise habits have given the disease a trend of getting younger. Therefore, early detection, diagnosis, and treatment should be the primary focus.^[^ ^6^ ^]^

As for atherosclerotic diagnosis, it mainly relies on ultrasound (US), magnetic resonance (MR), and computed tomography (CT) imaging, but they can merely identify advanced lesions, and the assessment of plaque vulnerability is not accurate enough.^[^ ^7^ ^]^ Furthermore, conventional contrast agents are only able to assist in morphological detections. Therefore, additional alternative measures are needed to optimize early diagnosis. Besides general treatment, atherosclerotic treatment mainly includes the best medical therapy (BMT) and surgery.^[^ ^8^ ^]^ On the one hand, the BMT refers to lipid‐lowering, antiplatelet, and antithrombotic treatments, which should be carefully selected based on the patient's condition. Nowadays, statin is mostly recommended to reduce circulating lipids by upregulating the low‐density lipoprotein receptor (LDLr) with the assist of apolipoprotein B (ApoB).^[^ ^9^ ^]^ However, long‐term drug treatment will cause liver damage, gastrointestinal bleeding, muscle discomfort, and arrhythmia.^[^ ^10^ ^]^ On the other hand, if severe stenosis happens, surgical intervention has to be considered, such as endarterectomy, balloon dilation, or stent implantation.^[^ ^11^ ^]^ While surgical treatment requires high demands on the patient's vital signs and may result in postoperative restenosis or bleeding complications.^[^ ^12^ ^]^ As a result, there is an urgent need to develop an accurate and efficient therapeutic method to reduce adverse reactions and improve treatment performance, which is more conducive to enhancing patient compliance and improving prognosis.

Nanotechnology integrates knowledge from various interdisciplinary fields including materials science, physics, biology, and chemistry, and is the result of the combination of modern science and technology.^[^ ^13^ ^]^ This technology explores the molecular world from a nanoscale perspective and has achieved widespread application in many fields, such as material engineering, biomedical science, environmental protection, electronic devices, and plant science.^[^ ^14^ ^]^ To date, the rapid development of nanotechnology and increasing clinical demands have given rise to the emerging interdisciplinary field of nanomedicine, bringing about a profound paradigm shift.^[^ ^15^ ^]^ Over the past few decades, a large number of nanomaterials used for disease diagnosis and/or treatment have been proposed, and the advantages of nanomaterials have gradually emerged: I) the ultra‐small size of nanomaterials provides a large surface area and more reaction sites;^[^ ^16^ ^]^ II) the physical and chemical properties of nanomaterials (e.g., optical, magnetic, mechanical, and electrical properties) can be regulated by the controllable composition and structure of the nanomaterials;^[^ ^17^ ^]^ III) the size of nanomaterials is much smaller than that of cells, which makes them easily penetrate through various biological barriers and have favorable biocompatibility;^[^ ^18^ ^]^ IV) the enhanced permeability and retention (EPR) effect promotes passive targeting accumulation of nanomaterials in tumors or other pathological sites;^[^ ^19^ ^]^ V) the off‐target effect of nanomaterials can be significantly reduced by modifying the active targeting ligand.^[^ ^20^ ^]^ These characteristics endow nanomaterials with potential value for clinical application.

Based on the continuous development of nanotechnology and the deepening of pathological research, nanomedicine has made breakthrough progress in the field of atherosclerosis, providing opportunities for precise individualized diagnosis and treatment.^[^ ^21^ ^]^ In general, atherosclerosis is characterized by excessive inflammatory burden, endothelial injury, neovascularization, and epigenetic abnormalities.^[^ ^22^ ^]^ According to these abnormalities, nanomedicine can be rationally designed and developed to differentiate these lesions from healthy tissues for subsequent diagnosis and/or treatment.^[^ ^23^ ^]^ In response to the problems existed in traditional imaging methods, the introduction of nanomaterials broadens the timing and scope of disease monitoring, achieving earlier and more comprehensive diagnosis with greater precision.^[^ ^24^ ^]^ Regarding adverse reactions associated with drugs or surgery, nanomaterials achieve higher drug utilization rates or further enrich the range of surgical options (such as nanodrug‐coated balloons or stents) through elaborated design.^[^ ^25^ ^]^ Importantly, nanomaterials provide the ability to integrate diagnosis and treatment for atherosclerosis. In the past, diagnosis and treatment were two separate fields. However, the surfaces or interiors of nanomaterials can easily couple with various components, making it possible to undergo treatment under imaging guidance or real‐time monitoring during treatment.^[^ ^26^ ^]^ This undoubtedly promotes ever‐greater advances and offers unprecedented solutions for disease exploration.

**Figure 1:**
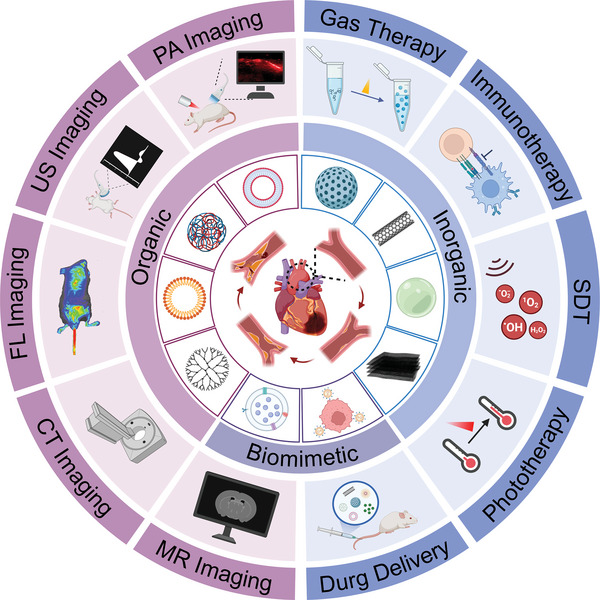
Schematic illustration of the application of nanomaterials in atherosclerosis, including atherogenesis (covering fatty streaks, fibrous plaque, atheromatous plaque, and secondary lesions), the representative classification of reported nanomaterials (including inorganic, organic, and biomimetic nanomaterials), diagnosis (e.g., magnetic resonance (MR), computed tomography (CT), fluorescence (FL), ultrasound (US), and photoacoustic (PA) imaging) and therapy strategies like drug delivery, phototherapy, sonodynamic therapy (SDT), immunotherapy, and gas therapy. Figure was created with BioRender.com.

To provide comprehensive information for researchers in this field, this review summarizes the pathological basis, development process, and corresponding animal models currently used in preliminary research. We also highlight the inorganic, organic, and biomimetic nanomaterials applied in the diagnosis and/or treatment of atherosclerosis and their physicochemical properties including particle size, shape, and potential. Furthermore, we provide a detailed and comprehensive introduction to the utilization of nanomaterials in the diagnosis (e.g., MR, CT, radionuclide, fluorescence (FL), photoacoustic (PA), optical coherence tomography (OCT), multi‐modal imaging and reported clinical trials), therapy (e.g., drug delivery, phototherapy, sonodynamic therapy (SDT), immunotherapy, gas therapy and reported clinical trials), and theranostic of atherosclerosis in recent years (**Figure** **1**). Besides, we summarize and conclude the issue on how to establish a biosafety evaluation system for nanomaterials, including but not limited to in vivo distribution and metabolism assessment, biological evaluation, immunological evaluation, and toxicology evaluation. Finally, existing challenges of nanomedicine in the atherosclerotic field are pointed out, and effective approaches and potential development directions for each issue are proposed to boost their entry into clinical practices.

## 2 Background and Foundation of Nanomaterials in Atherosclerosis

### 2.1 Pathological Characteristics

**Figure 2:**
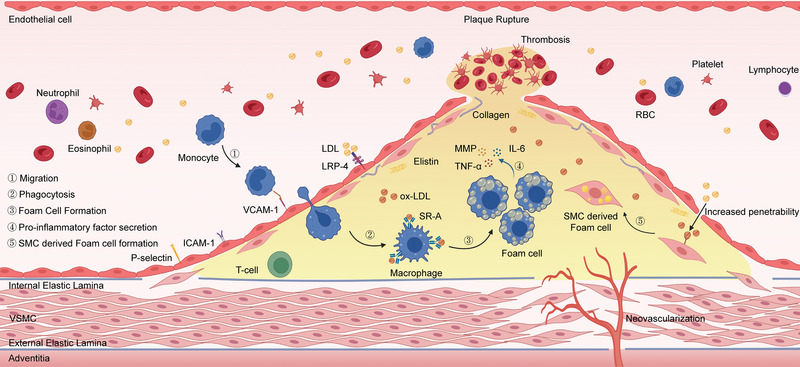
The diagrammatic sketch of the main components and the formation process of the atherosclerotic lesion. Figure was created with BioRender.com.

The term “atherosclerosis” originates from “atheroma” and “sclerosis,” where “atheroma” represents the atheromatous lesions formed by necrosis of lipid deposits and “sclerosis” refers to collagen fibrous hyperplasia. Macroscopically, the progression of atherosclerosis can be divided into four stages, including fatty streaks, fibrous plaque, atheromatous plaque, and secondary lesions. Besides, microscopic observation demonstrated the presence of endothelial cell damage, infiltration of inflammatory cells, migration of vascular smooth muscle cells (VSMCs), and lipid accumulation within the lesion. In this section, we mainly focus on the pivotal pathological components and progression within atherosclerosis (**Figure** **2**).

#### 2.1.1 Endothelium

Endothelium, the first barrier in contact with the blood, is normally in dynamic equilibrium with the various blood components.^[^ ^27^ ^]^ A series of irritants such as cholesterol, cigarette ingredients, hyperglycemia, and local hemodynamic disturbances cause endothelial cell dysfunction and impaired anatomical integrity. Increased endothelial permeability provides an opportunity for lipid to flow into subendothelial space, which in turn further activates the endothelium. Endothelial cell adhesion molecule (ECAM) is highly expressed in risk endothelium associated with atherosclerosis. In particular, vascular cell adhesion molecule 1 (VCAM‐1) binds to the very late antigen 4 (VLA‐4) on the monocytes and T cells, while intracellular adhesion molecule 1 (ICAM‐1) binds to lymphocyte function‐associated antigen 1 (LFA‐1) on neutrophils, which mediates the cellular adhesion.^[^ ^28^ ^]^ What is more, the up‐regulation of P‐selectin and E‐selectin also assists in the adherence of blood cells. Furthermore, activated endothelium secretes chemokines such as monocyte chemoattractant protein 1 (MCP‐1), granulocyte‐monocyte stimulating factor (GM‐CSF), and interleukin 8 (IL‐8) to strengthen the association of inflammatory cells with the lesion.^[^ ^29^ ^]^

#### 2.1.2 Lipoproteins

Lipids in the blood are transported in the form of lipoproteins. Briefly, a lipoprotein is made up of a single ApoB as well as the composition of triglycerides and cholesterol in different content. Whatever the content of the lipid, a lipoprotein with a diameter of less than 70 nm can penetrate the compromised endothelial barrier.^[^ ^30^ ^]^ Thereby, excess lipoproteins in plasma more easily enter the subendothelial space in the case of dyslipidemia.^[^ ^31^ ^]^ Among lipoproteins, the low‐density lipoprotein (LDL) has already been confirmed to promote atherogenesis.^[^ ^32^ ^]^ They deposit in the intima, where without plasma antioxidants, are easily oxidized by reactive oxygen species (ROS) produced by endothelial cells, macrophages, and VSMCs to proatherogenic and proinflammatory oxidized low‐density lipoprotein (ox‐LDL), which has explicit associations with clinical atherosclerotic cardiovascular disease.^[^ ^33^ ^]^ Compared to LDL, ox‐LDL is a more influential atherogenic lipoprotein throughout atherosclerotic formation and development. It promotes the secretion of various proinflammatory cytokines of endothelial cells,^[^ ^34^ ^]^ damages endothelial cells, reduces the gene expression of endothelial nitric oxide synthases (eNOS) and nitric oxide (NO) production,^[^ ^35^ ^]^ and weakens the self‐protective function of the vessel wall. In addition, it binds to and up‐regulates scavenger receptors (SR) on macrophages as auto‐antigen, accelerates the foaming process of macrophages and chemokines secretion, thereby, further amplifying the inflammatory cascade response.^[^ ^36^ ^]^ What's more, VSMCs are stimulated to proliferate and migrate from the media, phagocytose ox‐LDL with oxidized LDL receptor 1 (LOX‐1), and produce collagen‐rich matrix, conferring plaque stability and mediating luminal narrowing.^[^ ^37^ ^]^

#### 2.1.3 Inflammatory Cells

Initially, multiple inflammatory cells respond to the call of the activated endothelium, and especially circulating monocytes will recruit (rolling, activation, and arrest) and differentiate into macrophages in the intima. Monocyte‐derived macrophages engulf lipoproteins and switch them to foam cells via the SR, such as CD36 and scavenger receptors A1 (SR‐A1).^[^ ^38^ ^]^ Subsequently, foamy macrophages within the plaques secrete various cytokines such as MCP‐1, chemokine receptor 2 (CCR2), chemokine receptor 5 (CCR5), etc. to further recruit bone marrow cells. While in advanced plaque, macrophage proliferation in situ seems to play a crucial role.^[^ ^39^ ^]^ It is noteworthy that the number of macrophages in vulnerable plaques is three to five times higher than in stable plaques, hence the enrichment of macrophages becomes an important indicator of plaque vulnerability.^[^ ^40^ ^]^ These macrophages attempt to eliminate or efflux cholesterol to high‐density lipoprotein (HDL) with cholesterol efflux transporters ATP‐binding cassette transporter A1 (ABCA1) and G1 (ABCG1).^[^ ^41^ ^]^ Unfortunately, the SR won't down‐regulate its expression even though lipoproteins are already overloaded, so less efficient cholesterol efflux is observed compared with lipoproteins uptake. The foamy macrophages keep eating lipoproteins and finally have the inability to withstand undue stress and die.^[^ ^42^ ^]^

Macrophage apoptosis is beneficial in the early lesions because normal efferocytosis (refers to the engulfment of cell corpses by phagocytes) usually clears apoptosis cells in several minutes.^[^ ^43^ ^]^ As the disease progresses, macrophage apoptosis and necrosis increase, while efferocytosis‐associated proteins such as milk fat globule‐EGF factor 8 (Mfge8) and Mer receptor tyrosine kinase (Mertk) are dysfunctional. Massive cell corpses are unable to be removed by the defective efferocytosis and accumulate within the plaque to gradually establish, and expand a necrotic core, which exacerbates plaque instability.^[^ ^44^ ^]^

Besides, neutrophils, the most abundant white blood cells, are also early contributors to plaque and are associated with poor clinical prognosis.^[^ ^45^ ^]^ Neutrophils release metalloproteinases (MMPs) and proteolytic proteins to disrupt the physical barrier of the endothelium, and deposit chemotactic proteins like cathelicidin on the endothelium to promote monocyte recruitment.^[^ ^46^ ^]^ After entering subendothelial space, neutrophils further release myeloperoxidase (MPO), MMPs, elastases, cathelicidin, cathepsin G, and ROS, enhancing the oxidative stress levels and plaque vulnerability.

The bone marrow response is accompanied by the infiltration of adaptive immune systemic T cells and B cells. T cells are classified into multitude subtypes, of which CD4^+^ T cells are closely correlated with atherosclerotic progression.^[^ ^47^ ^]^ The T helper 1 (Th1) cells differentiated from CD4^+^ T cells are the most abundant T cell subpopulation in the lesion after antigen presentation, while the role of other T cell subtypes requires further confirmation.^[^ ^48^ ^]^ The investigation of B cells has lagged behind other cells, and early studies demonstrated that there were significantly fewer B cells in intimal plaques than in the adventitia.^[^ ^49^ ^]^

#### 2.1.4 Vascular Smooth Muscle Cells (VSMCs)

The balance of VSMCs proliferation and death is relevant to atherogenesis.^[^ ^50^ ^]^ To repair the injury, VSMCs within the media will migrate into the intima through the internal elastic lamina, proliferate and secrete extracellular matrix including collagen, elastin, and proteoglycan to form a fibrous cap that prevents plaque rupture and intrude into the arterial lumen.^[^ ^51^ ^]^ Over time, the senescence and death (especially apoptosis) of VSMCs contribute to a thin fibrous cap, large necrotic core, and inflammation activation, which are important features of the vulnerable plaque sites (mainly the shoulder).^[^ ^52^ ^]^ In addition, the loss of VSMCs induces medial atrophy, vascular calcification, vessel remodeling, and even the formation of aneurysm.^[^ ^53^ ^]^

With further studies, much plasticity of VSMCs was demonstrated that they can convert to other cell phenotypes from contractile phenotype during atherogenesis.^[^ ^54^ ^]^ Recent evidence suggests that they are derived into a transitional cell called “SEM” cells with versatility and such “SEM” cells have the potential to dedifferentiate or differentiate into macrophage‐like cells.^[^ ^55^ ^]^ To some degree, it explains why some VSMCs possess macrophage markers and can phagocytose lipoproteins to form VSMCs‐derived foam cells. Another important message is that judging a certain cell type within plaque barely based on a specific surface marker may be oversimplified.

Although there is no unanimous conclusion about the mechanisms of atherosclerotic formation, it is widely accepted that its internal environment is an intricate inflammation network in which both innate and adaptive immunity are involved. The above‐mentioned cells play the role of a double‐edged sword as the characteristic components of the atherosclerotic lesions. Therefore, the stage of disease progression, cellular origin, and function should be considered comprehensively when developing therapy strategies.

### 2.2 Experimental Animal Models

Direct atherosclerotic studies in humans are time‐consuming and difficult to obtain ideal specimens with many uncertain risks and confounding factors. Therefore, experimental animal models with similar pathogenesis and clinical manifestations to human atherosclerosis are needed for alternative studies. Currently, animals used to construct atherosclerotic models chiefly include mice, rats, rabbits, pigs, and non‐human primates. The modeling methods cover genetic engineering, high‐fat and high‐cholesterol feeding, surgical damaging as well as the introduction of risk factors (e.g., diabetes or pneumonia). In this section, we discuss the pros and cons of common experimental animal models and typical experimental modeling methods (**Table** **1**).

**Table 1:** The advantages, disadvantages, and representative models of different animal models.

Animal models	Advantages	Disadvantages	Representative models
Mice	Easy availability, high reproductive capacity, controlled conditions, short experimental periodicity low price	Limited plaque lesions, difficult operation because of thin blood vessels, abnormal inflammation and immune function.	C57BL/6J mice with a Western diet, ApoE‐/‐ mice, LDLr‐/‐ mice, ApoE/LDLr‐/‐mice, ApoE3‐Leiden mice ApoE‐/‐Fbn1^C1039G+/−^ mice
Rats	Large volume, easy operation	No gallbladder, difficult lipid absorption significant physiological differences to humans	Wistar or SD rats with a Western diet, I and vitamin D injection ApoE and/or LDLr‐/‐mice
Rabbits	Easy operation, similar anatomical structures and lipid metabolism to humans, high absorption of cholesterol.	High cost, inflammatory and hepatotoxicity, long duration or surgery required	New Zealand rabbits with a Western diet, I and balloon injury surgery, WHHL rabbits
Large animal models	Human‐like predilection site, homologous intimal structure,	High cost, long feeding time, small simple size, large individual differences	Porcine model non‐human primates

#### 2.2.1 Mice

Mice are the most common experimental animals due to their easy availability, docile temperament, high reproductive capacity, and controlled conditions. For example, C57BL/6J mice, the popular strain of atherosclerotic mice, are fed a high‐cholesterol and high‐fat diet to mimic the hyperlipidemia environment in humans. However, in terms of plaque formation, C57BL/6J mice need a long feeding period and form small plaques, thus genetic editing is introduced to regulate lipid metabolism. Apolipoprotein E knockout (ApoE‐/‐) mice and LDL‐receptor deficient (LDLr‐/‐) mice are the most widely applied models since the appearance of genetic editing.^[^ ^56^ ^]^ Apolipoprotein E (ApoE), an arginine‐rich protein, is found in many lipoproteins (e.g., chylomicron, chylomicron remnants, VLDL, LDL, and some HDL) and possesses anti‐inflammatory, antioxidant, and anti‐atherosclerosis effects.^[^ ^57^ ^]^ Generally, ApoE binds to LDLr on hepatocytes to participate in the uptake and degradation of lipoproteins by the liver, clears chylomicron via LDLr‐related proteins, engages in reverse cholesterol transport, and activates fat hydrolases.^[^ ^58^ ^]^ LDLr is a multifunctional protein widely distributed on the surface of hepatocytes, mediating the entry of plasma LDL into cells, regulating cholesterol levels, and exerting a hypolipidemic effect. Hence, the plasma cholesterol level is significantly elevated in LDLr‐/‐ mice.^[^ ^59^ ^]^ Although both are suitable for atherosclerotic experiments, it is noteworthy that ApoE‐/‐ mice can generate plaque with a normal diet, suggesting the difference in atherosclerotic mechanism between the two models. And in fact, when fed with a high cholesterol and high‐fat diet, ApoE‐/‐ mice exhibit higher cholesterol levels in plasma, larger plaque volumes, and greater necrotic cores compared to LDLr‐/‐ mice, making the former more widely used.^[^ ^60^ ^]^ Soon after, the ApoE/LDLr double knockout (ApoE/LDLr‐/‐) experimental mice were proposed by Bonthu and co‐workers. Faster lesion formation was observed in this model and it promised to investigate atherosclerosis with a normal diet.^[^ ^61^ ^]^ Additionally, to get closer to human lipid metabolism, ApoE3‐Leiden mice were created with a similar lipoprotein profile to human.^[^ ^62^ ^]^ Functional ApoE could be detected in ApoE3‐Leiden mice, and the effect of dyslipidemia on disease progression can be explored separately without the disturbance of inflammation compared to ApoE‐/‐ mice.^[^ ^63^ ^]^

The above genetically manipulated mice have generally matured with their own advantages, but still struggle to construct thrombosis, plaque rupture, and other complex complications. To address it, the cuff, artery partial ligation or angiotensin II infusion can be performed to artificially induce further endothelial damage and hemodynamic disturbances, thereby accelerating plaque formation, hemorrhage, and rupture.^[^ ^64^ ^]^ Another option is ApoE‐/‐Fbn1^C1039G+/−^ mice, a mutant heterozygote with a propensity for fibrin breakage, which develops highly unstable plaques that rupture spontaneously or even die suddenly.^[^ ^65^ ^]^ Combined with a western diet (21% fat and 0.15% cholesterol), ApoE‐/‐Fbn1^C1039G+/−^ mice can exhibit characteristic human‐like advanced plaques, which provides the opportunity to contribute to the research of vulnerable plaque.

#### 2.2.2 Rats

In general, Wistar or SD rats are also needed to establish the atherosclerotic model. Rats have an absent gallbladder and low lipid uptake, requiring prolonged feeding with a diet containing bile salts, thiouracil, high fat, and high cholesterol.^[^ ^66^ ^]^ High‐fat and high‐cholesterol feeding combined with intraperitoneal vitamin D injection are more popular since it promotes blood calcium absorption to damage the vascular endothelium.^[^ ^67^ ^]^ However, no available evidence to support the superiority of rats over mice. Similarly, to induce hyperlipidemia and atherosclerotic formation, ApoE‐/‐,^[^ ^68^ ^]^ LDLr‐/‐,^[^ ^69^ ^]^ or double knockout rats^[^ ^70^ ^]^ were constructed synchronously. Besides, there are significant physiological differences between rats and humans, thereby, conclusions drawn from rats are not necessarily applicable to humans. Notably, invasive manipulation such as balloon injury, electroshock injury, and arterial clamping and ex vivo analysis is more easily achieved in rats due to their volume superiority.

#### 2.2.3 Rabbits

Rabbits, the well‐established animal model for atherosclerosis, possess more similar lipid metabolism to humans than mice. At present, rabbit models used to study lipid metabolism and atherosclerosis include rabbits fed with a Western diet, Watanabe heritable hyperlipidemic (WHHL) rabbits, and genetically edited rabbits. First, New Zealand rabbits are usually chosen to develop the atherosclerotic lesions by 12–16 weeks and establish advanced plaque for more than 28 weeks with a high cholesterol diet (0.3%–1%).^[^ ^71^ ^]^ It is emphasized that rabbits fed with high cholesterol for a long time are prone to develop inflammation, hepatotoxicity, and fatty liver, which can incorporate balloon endothelial debridement to shorten the feeding period. Second, WHHL rabbits are characterized by more LDLs and fewer HDLs owing to defective LDLr expression, which is analogous to human familial hypercholesterolemia and atherosclerosis.^[^ ^72^ ^]^ Furthermore, genes involved in lipid metabolism could be introduced into or knocked out from rabbits by gene editing, providing new insights into the understanding of atherosclerosis.^[^ ^73^ ^]^ Nevertheless, rabbits are not a substitute for rodents as each rabbit requires a separate cage space, longer feeding time, and expense, and they are not inbred and vary widely among individuals. Overall, rabbits are more suitable for the studies of hyperlipidemia, clinical translation of drugs or pre‐experiments in large experimental animal models (pigs and monkeys).

#### 2.2.4 Large Animal Models

Small animal models are available for mechanistic exploration as well as preliminary pharmacological and toxicological studies. However, in order to apply previous research to the human body and achieve atherosclerotic diagnosis and treatment, other more appropriate animal models are needed.^[^ ^74^ ^]^ The atherosclerotic pig model is a promising bridging tool, and shares many similarities with human progressive atherosclerosis, including human‐like predilection site, homologous intimal structure, abundant neovascularization, and internal calcification.^[^ ^75^ ^]^ Pigs share similar omnivorous preferences with humans and possess a cardiovascular system of comparable size, making them more susceptible to the heightened fat content in their diets and corresponding vascular responses.^[^ ^76^ ^]^ Existing research manifested that nicotine injection combined with balloon injury can rapidly promote intimal hyperplasia, successfully inducing atherosclerotic pigs within one month.^[^ ^77^ ^]^ In addition, exerting extra for pigs PCSK9 gene mutation or somatic nuclear transfer aided by a high‐fat diet is also a kind of promising technique.^[^ ^78^ ^]^ Besides, the conclusions drawn from non‐human primates have high applicability in humans because of their comparative physiological similarity to humans.^[^ ^79^ ^]^ The development of gene editing tools may provide new approaches and reinforce the similarity between large animal models and human atherosclerosis.^[^ ^80^ ^]^ However, working with large animals is expensive, time‐consuming, highly regulated, and requires extremely specialized laboratory skills and a dedicated experimental site.

Therefore, the use of mouse models is recommended for the initial exploration of early drug screening, efficacy verification, mechanism clarification, and safety evaluation. Considering surgical manipulation, plaque imaging manifestation, and preliminary validation of large animal models, rats, and rabbits may be better choices. As research progresses and prepares for clinical trials, conducting verification in large animal models is crucial.

### 2.3 Material Design

To obtain benign anti‐atherosclerosis effects, some general principles are supposed to be considered when constructing nanomaterials. Nanomaterials should possess superior bioavailability, biocompatibility, stability, permeability, and desirable circulation time in vivo under physiological conditions, as well as accumulate at the target location as much as possible without damaging the surrounding healthy tissues via passive or active targeting.

#### 2.3.1 Passive Targeting

Passive targeting refers that the nanomaterials being recruited to the plaque either through the high permeability of the leaky vasculature or the phagocytosis by the mononuclear phagocyte system (MPS). Similar to cancer, the EPR effect may be informative due to the abundant neovascularization and abnormal arrangement of endothelial cells within atherosclerosis. However, the EPR effect remains to be investigated because it is unpredictable in animal models and the endothelial junctions tend to be stabilized in advanced plaques. Another possibility is that nanomaterials are selectively ingested by certain immune cells, which carry a considerable proportion of the nanomedicines into the plaque.^[^ ^81^ ^]^ These two routes allow the nanomaterials to “passively target” the diseased organ or site, although the specific mechanism is not completely understood. Notably, it is highly dependent on the physicochemical characteristics of nanomaterials such as size, shape, and surface properties.^[^ ^82^ ^]^

First, size has a profound impact on the biodistribution, pharmacokinetics, and half‐life of nanomaterials upon intravenous injection. Nanomaterials whose size are smaller than 10 nm are easily cleared directly by the kidney, whereas nanomaterials are preferentially captured by the reticuloendothelial system in the liver and spleen when their size is larger than 50 nm, and oversized nanomaterials (micron‐size) are difficult to enter plaque.^[^ ^83^ ^]^ It was demonstrated that as the size of nanomaterials increases in a limited range (from 40 nm to 200 nm), more capture by the reticuloendothelial system and shorter blood circulation times were observed. In a comparative study, polyion complex micelles of different sizes were constructed to evaluate the accumulation at the lesion site in a rat carotid balloon dilation model.^[^ ^84^ ^]^ Their work demonstrated that variation in the size of nanomaterials influenced their accumulation and therapeutic effects. After that, the same group further confirmed that resizing nanomaterials could adjust their interaction with macrophages in vascular diseases.^[^ ^85^ ^]^

Second, several studies have suggested that the shape of nanomaterials (e.g., spherical, rod, disk, wire, oblate ellipsoids, etc.) affects the interaction between nanomaterials and cells, circulating half‐life, and margination behavior. Although non‐spherical nanomedicines are more challenging to prepare than spherical nanomaterials, they seem to be more favored in blood circulation and cell internalization.^[^ ^86^ ^]^ On the one hand, mononuclear macrophages in the liver and spleen internalize fewer nonspherical nanomaterials than spherical nanomaterials, thus prolonging the circulating half‐life.^[^ ^87^ ^]^ On the other hand, the curvature of spherical nanomaterials limits their contact area with the endovascular surface, lowering the tendency of margination and adhesion.^[^ ^88^ ^]^ In a plate flow chamber model, rod particles with high aspect ratios manifested superior adhesive properties in comparison with conventional spherical particles.^[^ ^89^ ^]^ Another research aimed to explore the influence of different shape parameters on the targeting thrombi function in mice models.^[^ ^90^ ^]^ Likewise, rod nanomaterials are more powerful in terms of targeting effect, and the influence of shape is much stronger than that of various targeting peptides. Therefore, nonspherical nanomaterials seem to be more suitable as carriers for targeting atherosclerosis. Furthermore, nanomaterials with different shapes exhibit a preference for various inflammatory cell subpopulations. Researchers designed three types of nanostructures with the same surface chemical properties, including micelles, polymersomes, and filomicelles, to investigate the selective uptake of cell subpopulations by structural morphology differences. The study indicated that polymersomes were preferentially taken up by macrophages and DCs in the spleen and lymph nodes with a longer residence time. Micelles showed superior specificity for macrophages and DCs in the liver, while filomicelles were preferentially taken up by granulocytes in the blood. In addition, DCs in atherosclerotic plaques demonstrated a significant preference for polymersomes.^[^ ^91^ ^]^

What is more, the surface property is another crucial aspect in the design of nanomaterials. For example, the surface charge affects cellular uptake and blood circulation time. The positively charged nanomaterials are more readily internalized by cells compared to their negative or neutral counterparts, probably due to the interactions with negatively charged sialic acid groups on the surface of macrophage membrane.^[^ ^92^ ^]^ The CD36 on macrophages or foam cells can slightly reinforce the internalization of the negatively charged nanomaterials. In addition, nanomaterials without surface modification are usually hydrophobic and easily cleared by macrophages in the liver and spleen, hindering the delivery of the nanomaterials. To address it, hydrophilic polymers like polyethylene glycol (PEG) or amphoteric polymers are applied to coat the nanomaterials, forming a hydrated shell.^[^ ^93^ ^]^ This surface coating can additionally work as an intermediate medium to modify more functional components into nanomaterials. Notably, nanomaterials are exposed to physiological fluids in vivo and apt to bind proteins or other biomolecules to form “protein corona”. The charged or hydrophobic nanomaterials tend to adsorb more proteins and induce protein denaturation than their neutral and hydrophilic counterparts.^[^ ^94^ ^]^ These protein crowns will alter the basic features of the nanomaterials including targeting performance, biodistribution, immunogenicity, and toxicity and even confer new functions on them.^[^ ^95^ ^]^

#### 2.3.2 Active Targeting

Active targeting typically entails attaching specific targeting agents to the nanoparticle or constructing a stimulus‐responsive smart nanocarrier for effective diagnosis and/or therapy. Compared to passive targeting, active targeting allows higher intra‐lesion localization and fewer side effects in a shorter time, due to the reduced single dosage and administration frequency.

For one thing, the specific pathological components and overexpressed molecules during atherosclerotic progression offer properly targeted goals for the active targeting of nanomaterials. Most of these active targeting strategies rely on the interaction between antigen and antibody as well as ligand and receptor. Currently, the active targeting aims of nanomaterials mainly include macrophages,^[^ ^96^ ^]^ endothelial cells,^[^ ^97^ ^]^ neovascularization,^[^ ^98^ ^]^ thrombus,^[^ ^99^ ^]^ and extracellular matrix proteins.^[^ ^100^ ^]^

Macrophage, a kind of phagocyte, acts as one of the most important cell types in atherosclerosis. The strategy for constructing actively targeted nanomaterials is mainly based on the typical surface marker (e.g., CD44, CD68, and CD47) and cellular phagocytic function of macrophage.^[^ ^101^ ^]^ This function involves high expression of CD36, SR‐A1, LOX‐1, mannose receptors, and phosphatidylserine (PS) receptors. After macrophages engulf lipids, foam cells are formed, and osteopontin (OPN) as well as ABCA1 are further abnormally expressed. Therefore, existing literatures have chosen corresponding antibodies, peptides, or ligands for modification of nanomaterials to achieve active targeting for these types of cells. Activated endothelial cells overexpress VCAM‐1, ICAM‐1, P/E‐selectin,^[^ ^102^ ^]^ αvβ3 integrin,^[^ ^103^ ^]^ and stabilin‐2,^[^ ^100^ ^]^ thus antibodies or peptides with high affinity to these biomarkers have been widely applied for active targeting. In addition, αvβ3 integrin is a promising candidate for the visualization or mitigation of neovascularization. In terms of thrombosis, platelets adhere and network fibrin and red blood cells together to form aggregates, and several ligands targeting fibrin and platelets have been developed to detect or competitively inhibit the formation of thrombus. In addition, the extracellular matrix proteins involve collagens, elastic fibers, laminins, and glycoproteins.^[^ ^104^ ^]^ These ingredients occupy more than half of the plaque area, so they can be actively targeted to reflect the degree of luminal stenosis and plaque stability.

For another, in the pathological transformation of the normal arterial wall structure, many variations in the lesion components occur, specifically the expression of molecules as described above, the change of the enzyme profile (such as matrix metalloproteinases within the atherosclerotic plaque site) and hemodynamics as well as the local microenvironment (e.g., pH, temperature and oxidative stress).^[^ ^105^ ^]^ Therefore, it is possible to construct therapeutic or diagnostic systems under specific stimulus‐response based on the differences between the interior of the lesion and the normal tissues, thereby mitigating the interference with normal cells. Representative stimulus‐response paradigms include a localized acidic environment‐sensitive chemical bond breaking,^[^ ^106^ ^]^ a ROS‐responsive drug release, and the transition between hydrophobic and hydrophilic states mediated by the lipid microenvironment within the atherosclerotic plaque.^[^ ^107^ ^]^

## 3 Nanomaterials

### 3.1 Inorganic Nanomaterials

According to the reported literature, inorganic nanomaterials applied to atherosclerosis can be divided into metallic and non‐metallic nanomaterials.

#### 3.1.1 Nonmetallic Nanomaterials

**Figure 3:**
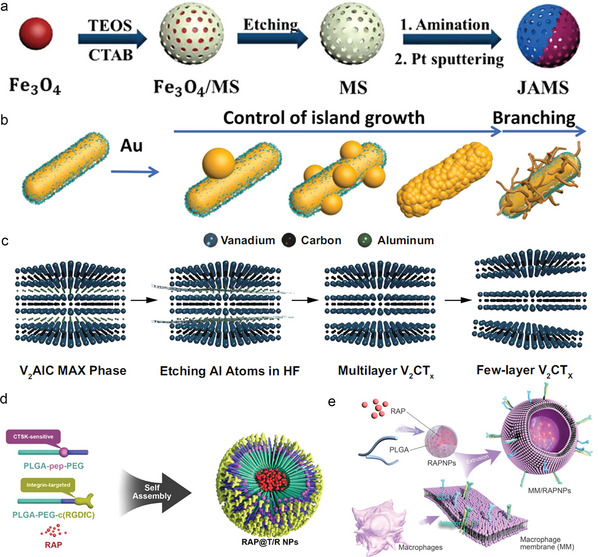
Representative schematic diagrams of typical nanomaterials such as a) MSNs, b) AuNPs and c) MXene. a) Reproduced with permission.^[^ ^109^ ^]^ Copyright 2020, Royal Society of Chemistry. b) Reproduced with permission.^[^ ^115^ ^]^ Copyright 2020, Wiley‐VCH. c) Reproduced with permission.^[^ ^119a^ ^]^ Copyright 2021, Springer Nature. d) Representative illustration of polymeric nanomaterials (RAP@T/R NPs). Reproduced with permission.^[^ ^124^ ^]^ Copyright 2022, Ivyspring International Publisher. e) Schematic diagram of biomimetic cell membrane‐coated nanomaterials (MM/RAPNPs). Reproduced with permission.^[^ ^131^ ^]^ Copyright 2021, Ivyspring International Publisher.

Nonmetallic nanomaterials such as mesoporous silica and carbon‐based nanomaterials are widely used in atherosclerosis. Mesoporous silica nanoparticles (MSNs) have a particle size of 10–600 nm and a pore size of 2–50 nm with a large surface area, unique mesh‐like pore structure, regular pore channels, and adjustable pore size. The adjustable central pore structure and high loading of cargo facilitate its function as a nanocarrier to improve the water solubility of drugs and decrease the side effects.^[^ ^108^ ^]^ In addition to filling inward as a shell structure, MSN can also be used as a platform for the growth of other materials (**Figure** **3**a).^[^ ^109^ ^]^ Besides, carbon‐based nanomaterials come in a variety of shapes and types, and in recent years, a myriad of novel carbon‐based nanomaterials have attracted a lot of attention, such as graphene, carbon nanotubes, nanodiamonds, and nanodots, etc.^[^ ^110^ ^]^ Several carbon‐based nanomaterials own unique structures and properties with high photothermal conversion efficiency and distinct FL effect, which are suitable for photothermal therapy (PTT), PA imaging, and FL tracing (e.g., carbon nanotubes,^[^ ^111^ ^]^ carbon nanocages,^[^ ^112^ ^]^ graphene oxide,^[^ ^113^ ^]^ and carbon quantum dots).^[^ ^114^ ^]^

#### 3.1.2 Metallic Nanomaterials

Gold nanoparticles (AuNPs) are currently the most widely used metal nanomaterials in the field of nanomedicine and are usually obtained by the reduction of inorganic acids containing gold in the presence of stabilizers (Figure 3b).^[^ ^115^ ^]^ AuNPs have controlled morphology and such as spheres, nanorods, and nanocages. AuNPs‐based PTT and PA imaging for atherosclerosis are feasible due to the presence of surface plasmon resonance (SPR) effects, and CT tracing on the basis of AuNPs is possible because of the large atomic number.^[^ ^116^ ^]^ Among the metal oxides, iron oxide with marvelous magnetic properties can be applied to MR imaging. Cerium oxide (CeO_2_), cobalt oxide (Co_3_O_4_) and manganese oxide (Mn_2_O_3_, Mn_3_O_4_) nanoenzymes with multienzyme‐like activities are also gaining attention for their ability to scavenge ROS and alleviate inflammation.^[^ ^117^ ^]^ Semiconductor quantum dots (QDs) are excellent fluorescent tracers owing to their intrinsic optical characteristics.^[^ ^118^ ^]^ QDs have high photoluminescence quantum yields and photostability, which is promising in multimodal imaging and theranostic. Transition metal carbides, nitrides, and carbonitrides (MXene) have a 2D sheet‐like structure with a huge surface area and abundant functional groups, which can be used as a carrier of many objects (Figure 3c).^[^ ^119^ ^]^ Additionally, the light absorption and photothermal conversion efficiency in the near‐infrared region (NIR) of MXene is helpful for atherosclerotic PA imaging.^[^ ^120^ ^]^

### 3.2 Organic Nanomaterials

#### 3.2.1 Lipid‐Associated Nanomaterials

Lipid‐associated nanomaterials mainly include liposomes and reconstituted high‐density lipoprotein nanoparticles (rHDL NPs). Liposomes have a phospholipid bilayer structure, which is composed of components such as cholesterol and phospholipids in a self‐assembly behavior. As an excellent drug delivery vehicle with high biocompatibility, a wide range of deliverable drugs, easy surface modification, and controllable drug release, liposomes have been widely used in a variety of disease models.^[^ ^121^ ^]^ Similarly, rHDL is composed of endogenous apolipoprotein A‐1 (ApoA‐1), phospholipids, cholesterol, and cholesteryl esters inherent in the body. Cholesteryl esters act as the hydrophobic core that can encapsulate hydrophobic substances, and the other components act as the hydrophilic surfaces that can carry hydrophilic components. Particularly, rHDL NPs have anti‐inflammatory and antioxidant effects, and mediate the reverse cholesterol transport in foam cells via ABCA1, ABCG1, and SR‐1 to alleviate the lipid burden, making it especially suitable for atherosclerotic treatment.^[^ ^122^ ^]^

#### 3.2.2 Polymeric Nanomaterials

Polymeric nanomaterials, with diverse structures and compositions, could be fabricated by using natural polymers including dextran, cyclodextrin and chitosan, as well as synthetic polymers such as poly(ethylene glycol) (PEG), poly(ethylene imine) (PEI), Poly‐L‐lysine (PLL), polyamidoamine (PAMAM) and poly (lactic co‐glycolic acid) (PLGA).^[^ ^123^ ^]^ They have strong plasticity and can introduce surfactants with different structures, thereby fulfilling various changes in biological functions (Figure 3d).^[^ ^124^ ^]^ On the one hand, many drug carriers or nanoparticles use polymers as surface coatings and exist in the form of polymeric micelles. Polymeric micelles have high stability and biodegradability and are apt to reduce phagocytosis of the reticuloendothelial system to achieve longer blood circulation time. On the other hand, polymers participate in the construction of dendrimers. Dendrimers are artificial macromolecules with tree‐like shapes, which have a well‐defined 3D structure and are usually composed of a core, polymeric chains, and surface functional groups. The internal cavity structure assists drug loading, the polymerization algebra is easy to adjust, and a large number of functional groups on the surface facilitates molecular coupling, enabling precise control of size, shape, and function.^[^ ^125^ ^]^

### 3.3 Biomimetic Nanomaterials

In recent years, biomimetic nanomaterials have gained widespread attention as a novel delivery strategy.^[^ ^126^ ^]^ These biomimetic nanomaterials mainly consist of nanoparticles and external biomimetic components, including biomimetic cell membranes, living cells, viruses, and bacteria. They retain the intrinsic characteristics of nanoparticles while acquiring extra properties of biological structures (e.g., immune escape, long circulation, and targeted delivery). Among them, cell membrane‐coated nanoparticles are popular in the treatment of atherosclerosis and are obtained by co‐extrusion methods or ultrasound methods.

Currently, the BMT used to regulate inflammation and lipids in atherosclerosis is often limited by the first‐pass effect and local low blood drug concentrations. Biomimetic nanomaterials endow nanoparticles with a longer circulation time, lasting drug release capabilities, and higher drug utilization. This may be related to the negative charge carried by cell membranes, which can reduce the rapid adsorption of most positively charged nanoparticles with cells in the bloodstream. Erythrocyte membranes were developed to encapsulate rapamycin, which took advantage of natural “don't eat me” CD47 molecules on the surface to evade phagocytosis, prolonged the half‐life in the bloodstream, and achieved targeted slow release of drugs across the leaks of endothelial cells and atherosclerotic microvessels.^[^ ^127^ ^]^ Additionally, gene editing technology could introduce functional probes like peptides, proteins, small molecules, etc. into red cells to achieve functional expansion, however, this section remains to be studied.^[^ ^128^ ^]^ Endothelial cell membranes, due to the large number of self‐recognition proteins on the surface, had homologous targeting ability, making them efficient candidates for targeted atherosclerosis.^[^ ^129^ ^]^ According to literature reports, platelets^[^ ^130^ ^]^ and macrophages^[^ ^131^ ^]^ possess positive inflammatory tropism,^[^ ^132^ ^]^ which may be another alternative method (Figure 3e). Among them, nanoparticles coated with macrophage membranes are more favored. It has been found that the antigens such as the SR on the surface of macrophages can bind ox‐LDL, reducing the uptake of lipids by macrophages and inhibiting the formation of foam cells. Moreover, the presence of membrane antigens sequestered key proinflammatory factors and chemokines, which was superior to live cell delivery in reducing inflammation within plaques.^[^ ^133^ ^]^

In summary, biomimetic nanomaterials exhibit improved pharmacokinetics, excellent plaque‐targeting ability, and controllable engineered design, some of which even have therapeutic effects, providing a promising platform for the treatment of atherosclerosis.

## 4 Diagnostic Imaging

For most, before the outbreak of the endpoint event, atherosclerosis is a relatively recessive disease without corresponding overt characteristics. Once the disease advances, the efficacy of drug treatment is limited, and the surgical treatment is accompanied by great damage. Therefore, scientists desperately wish that diagnostic tools could fulfill early diagnosis, provide an accurate assessment of atherosclerotic burden, and clarify future risk stratification, which is hard to achieve with conventional imaging tools and their contrast agents. In recent decades, a variety of nanoprobes flooded into our horizon as the nanoscale size allows easier access to the interior of plaque and more intuitive visualization of the pathological process by the EPR effect. Besides, coupled with antibodies, polypeptides, or other targeting ligands, the nanoprobes can bind specifically to the surface molecules of certain cells or tissues to evaluate specific mechanisms or processes. An increasing number of research demonstrated that the application of nanoprobes can facilitate drug delivery, reduce dosage, enhance the ability to anticipate risks, and guide the subsequent treatment. Obviously, the employment of nanoprobes is opening a new door in atherosclerotic diagnosis.^[^ ^134^ ^]^ We summarize the basic features of nanoprobes for atherosclerotic diagnosis in recent years (**Table** **2**).

**Table 2:** A summary of the application of nanomaterials in atherosclerosis.

Application method	Modality	Nanomaterials	Size	Targeting strategy	Animal models	Biomedical applications and mechanisms	Refs.
Diagnosis	MR imaging	Tropoelastin‐binding peptide coupled tetrameric Gd_4_‐TESMA	/	Tropoelastin‐binding peptide (the VVGS‐peptide) target tropoelastin in plaque	ApoE ‐/‐ mice	Gd‐mediated T1 weighted MR imaging	[139]
		DTPA/fucoidan/peptide complex nanoparticles	243.6 nm ± 10.0 nm (TEM)	Target P‐selectin overexpressing endothelial cells.	/	Gd‐mediated T1 weighted MR imaging	[140]
		Cy5.5‐OPN‐DMSA‐MNPs (COD‐MNPs)	DMSA‐MNPs core: 7.3 nm ± 0.6 nm (DLS)	OPN antibody target OPN on foam macrophage	ApoE ‐/‐ mice	Iron oxide nanoparticles‐mediated T2 weighted MR imaging	[142]
		Hyaluronan‐conjugated iron oxide nanoworms (HA‐NWs)	The length of HA‐NW: 65 nm ± 15 nm	Hyaluronan target CD44 on endothelium cells	ApoE ‐/‐ mice	Iron oxide nanoparticles‐mediated T2 weighted MR imaging	[144]
	CT imaging	N1177	259 nm	Phagocytosis by macrophages and quantification of macrophage burden	High‐fat fed and balloon damaged New Zealand White rabbits	Iodinated nanotracer‐mediated CT imaging	[151]
		Fib‐GC‐AuNP	127.4 nm ± 2.7 nm	Target fibrin in thrombi	C57Bl/6 mice with FeCl_3_‐induced carotid thrombosis and/or embolic ischemic stroke	AuNPs‐mediated CT imaging	[153]
		11‐MUDA AuNP	31.5 nm	Label and track monocytes	ApoE‐/‐ mice	AuNPs‐mediated CT imaging	[154]
		AuNP	12.5 nm	Phagocytosis by macrophages and quantification of macrophage burden	Balloon damaged New Zealand white rabbits	AuNPs‐mediated CT imaging	[156]
	Radionuclide imaging	^18^F‐Sodium Fluoride	/	Calcification imaging	Excised carotid specimens	^18^F‐mediated PET imaging	[157]
		^89^Zr‐DFO‐Gal_3_‐F(ab')_2_ mAb	/	Target the Galectin‐3 and macrophage	ApoE ‐/‐ mice	^89^Zr‐mediated PET imaging	[159a]
		^89^Zr‐labeled maleylated human serum albumin (^89^ Zr‐HSA)	/	Target the macrophage scavenger receptor SR‐A1	ApoE ‐/‐ mice	^89^Zr‐mediated PET imaging	[159b]
		^89^Zr‐labeled liposomes	106.0 nm ± 5.2 nm	Passive targeting	High‐fat fed and balloon damaged New Zealand White rabbits	^89^Zr‐mediated PET imaging	[161]
		^64^Cu‐DAPTA‐Combs	10%DAPTA‐Comb 14.8 nm, 25%DAPTA‐Comb 15.2 nm, 40%DAPTA‐Comb 10.9 nm	Target the CCR5 in plaque	ApoE ‐/‐ mice and excised carotid specimens	^64^Cu‐mediated PET imaging	[162]
		H‐ferritin nanocages(^99^mTc‐HFn)	16 nm	Phagocytosis into macrophages and quantification of macrophage burden	ApoE ‐/‐ mice	^99^mTc‐mediated SPECT imaging	[163]
	FL imaging	ApoA‐I‐Cy5.5	/	Protease‐activatable fluorescent probe to analyze ApoA‐I‐degrading activities	Human ApoB transgenic LDL‐/‐ atherosclerotic mice (ATX)	Cyanine‐mediated FL imaging	[165]
		TBNG@Mp	100 nm to 120 nm	Platelet membrane target foam macrophage	SD rats with high‐fat diets and vitamin D3	Naphthalimide‐mediated FL imaging	[166]
		Pyrene‐based naked‐eye colorimetric and fluorescent turn‐on probe (S‐ClO)	/	Imaging endogenous HClO^−^, ClO^−^promoted oxidative cleavage reaction	RAW264.7 cells, an arterial vessel inflammation nude mouse model and human serum	Pyrene‐mediated FL imaging	[168]
		IONP‐ICG	IONP‐ICG5 (35.9 nm ± 8.2 nm), IONP‐ICG10 (34.1 nm ±10.1 nm), IONP‐ICG20 (34.9 nm ± 9.2 nm)	Passive target	Spontaneously hyperlipidemic (SHL) mice with a disrupted ApoE gene	Indocyanine green (ICG)‐mediated FL imaging	[169]
		P‐ICG2‐PS‐Lip	180 nm (TEM)	Phosphatidylserine(PS) target phosphatidylserine receptor on macrophages	ApoE ‐/‐ mice and WHHL rabbits	Indocyanine green (ICG)‐mediated FL imaging	[172]
		Anti‐CD47‐modified TPE‐T‐RCN NPs (anti‐CD47 NPs)	105.1 nm ± 0.2 nm (DLS)	Anti‐CD47 antibody target CD47 on SMC and macrophages	ApoE ‐/‐ mice and excised carotid specimens	D‐π‐A type AIE luminogen‐mediated FL imaging	[173]
		PbS/CdS/ZnS quantum dots	5.6 nm (TEM) 20 nm (DLS)	Highly penetrating thermal transient thermometry discriminated between the ischemic and inflammatory phases	A murine model of hindlimb ischemia	QDs‐mediated FL imaging	[176]
		Dextran‐mimetic HgCdSe‐based QDs	5.5 nm ± 0.9 nm (TEM)	Dextran target macrophages	C57BL/6 mice with HFD	QDs‐mediated FL imaging	[177]
	PA imaging	OPN Ab/Ti_3_C_2_/ICG nanoprobes	Lateral size of less than 500, the thickness of about 1.5 nm (TEM)	OPN antibody target OPN on foam cell	ApoE ‐/‐ mice	Ti_3_C_2_ and ICG‐mediated PA imaging	[120]
		Ultraselective carbon nanotubes (SWNTs)	2 nm × 200 nm (width ×length)	Target the Ly‐6Chi monocytes subpopulation	FVB mice with high‐fat diet; intraperitoneal injections of streptozotocin; ligate left common carotid artery	SWNTs‐mediated PA imaging	[111]
		PBD‐CD36 nanoparticles	50 nm (TEM)	CD36 antibody target CD36 on inflammatory cells	ApoE ‐/‐ mice	Semiconductor nanomaterial PBD‐mediated PA imaging	[182]
		Ratiometric semiconducting polymer nanoparticles (RSPN)	20 nm (TEM)	Passive target	ApoE ‐/‐ mice and ApoE‐/‐ mice complicated with pneumonia	Semiconductor polymer nanomaterial‐mediated PA imaging	[183]
	OCT	AuNPs	/	Passive target	Non‐animal	AuNPs‐mediated OCT imaging	[186]
		Semiconductor QDs	5 nm ± 2 nm (TEM)	Passive target	Rabbits	QDs‐mediated OCT imaging	[187]
	Multimodal imaging	Zinc‐doped ferrite nanoparticles (Zn_0.4_Fe_2.6_O_4_)	zinc doped ferrite (ZF) core:24 nm ± 2 nm (TEM)	Heat shock protein (Hsp)‐70 antibodies target atherosclerotic inflammatory site.	ApoE ‐/‐ mice	Zinc‐mediated CT imaging, Ferrite nanocomplex‐mediated MR imaging	[188]
		VUSPIO‐TEG4	/	The scFv fragment of TEG4 antibody target the activated platelets (integrin αIIbβ3)	ApoE ‐/‐ mice and New Zealand rabbits (NZW)	Fluorochromes‐mediated FL imaging, Iron oxide nanoparticles‐mediated MR imaging	[189]
		PP1‐IO@MS‐IR820, PIMI	90 nm (TEM)	PP1 target scavenger receptors A1 (SR‐A1) on foam macrophages	ApoE ‐/‐ mice	NIRF dye (IR820)‐mediated FL imaging, Iron oxide nanoparticles‐mediated MR imaging	[190]
		^89^Zr/BODIPY‐labeled ^19^F‐HDL	Small: 40 nm middle: 105 nm, large:180 (DLS)	HDL target myeloid cells	ApoE ‐/‐ mice	^19^F‐HDL‐mediated ^19^F MR imaging, ^89^Zr‐mediated gamma imaging, Cholesteryl BODIPY‐mediated optical techniques	[81]
		5‐HT‐Fe_3_O_4_‐Cy7 nanoparticles (5HFeC NPs)	20.97 nm ± 2.23 nm (TEM) 50.27 nm ± 18.96 nm (DLS)	5‐hydroxytryptamine (5‐HT) target active MPO	ApoE ‐/‐ mice	Iron oxide nanoparticles‐mediated magnetic particle imaging, Cy7‐mediated FL imaging, CTA for anatomical information	[191]
Therapy	Drug delivery	Simvastatin/EGCG‐coloaded liposomes (SE LNPs)	20 nm (TEM), 208.90 nm ±3.99 nm (DLS)	Passive target	ApoE‐/‐ mice	Remove ROS, inhibit apoptosis, induce M2 macrophage polarization, decrease inflammation cytokines	[203]
		Simvastatin‐loaded nanoliposomes	106.8 nm ± 22.5 nm (DLS)	Passive target	/	Anti‐inflammatory and promote cholesterol efflux	[204]
		GW3965‐loaded Lyp‐1 liposomes	77.5 nm ± 4.0 nm (DLS)	The cyclic peptide Lyp‐1 target to gC1q receptor (p32) on foam cell	LDLr‐/‐ mice	GW3965 activate LXR on foam cell, improve the induction of ABCA1/ABCG1, and promote the excess cholesterol efflux	[205]
		NLRP3 siRNA‐loaded VCAM‐1 binding peptide targeting cationic liposomes (siRNA PCLs).	138 nm ± 40 nm (DLS)	VCAM‐1‐binding peptide target VCAM‐1 on endothelial cells.	ApoE‐/‐ mice	Inhibit the formation of NLRP3 inflammasome, thereby inhibit subsequent inflammatory reactions	[206a]
		PPARγ agonist of pioglitazone (PIO)‐loaded, PtdSer and DSPE‐PEG2000‐cRGDfK modified liposome (AP‐Lipo)	About 120 nm	cRGDfK peptide target to ανβ3 integrin on endothelial cells.	ApoE‐/‐ mice	Decrease M1 macrophages, increase M2 macrophages, secrete anti‐inflammation cytokines	[207]
		Rapamycin (RAP)‐loaded, platelets membrane‐coated nanoparticles (RAP‐PNP)	145.7 nm ± 6.7 nm	Congenital adhesion of platelets to damaged vascular endothelium	ApoE‐/‐ mice	Anti‐inflammatory, reduce plaque burden	[208]
		Rapamycin (RAP)‐loaded hybrid platelets membrane biomimetic liposomes	About 90 nm (TEM and DLS)	Congenital adhesion of platelets to damaged vascular endothelium	ApoE‐/‐ mice	Anti‐inflammatory, reduce plaque burden	[209]
		SHP1i‐loaded, macrophage membrane‐coated biomimetic nanoparticles	199.1 nm(DLS)	The inherent protein on macrophage membrane ensures the actively chemotactic towards the inflammatory lesion site	ApoE‐/‐ mice	SHP1i block CD47‐SIRPα signaling pathway, enhance efferocytosis, stabilize atherosclerosis	[211]
		Colchicine‐loaded, modified macrophage‐membrane (MMM)‐coated PLGA nanoparticles	202.02 nm	Integrin α4/β1 target to vascular cell adhesion protein 1 (VCAM‐1) on vascular endothelial cells	ApoE‐/‐ mice	Strongly anti‐inflammatory and stable vulnerable plaque	[212]
		Simvastatin acid (SA)‐loaded dendrimer nanoparticles adsorbed toRBCs (SA PAM@RBCs)	About 100 nm (TEM)	I) Shear stress separate nanoparticles from RBCs II) ROS promotes SA PAM to release simvastatin acid	ApoE‐/‐ mice	Eliminate hydrogen peroxide, reduce lipid content, and reduce plaque area	[213]
		Quercetin (QT)‐loaded macrophage‐liposome conjugate (MP‐QT‐NP)	105 nm (DLS)	The inherent chemotactic ability of macrophage towards the inflammatory lesion site	ApoE‐/‐ mice	β‐CD dissolve plaque cholesterol, quercetin (QT) remove active oxygen, inhibit inflammation, promote polarization of M1 to M2 macrophages	[214]
		PEDF‐loaded, cRGD‐modified PEG‐PCL (PEDF RNPs)	148.2 nm ± 68.7 nm (DLS)	cRGD peptide target to ανβ3 integrin on endothelial cells.	ApoE‐/‐ mice	Inhibit angiogenesis, stabilize plaque, and prevent plaque rupture	[215]
		ROS‐responsive simvastatin nanoprodrug combined ticagrelor‐loaded drug delivery system (TPTS/C/T)	189 nm (DLS)	ROS responsive and fibronectin‐targeted	ApoE‐/‐ mice	Anti‐inflammatory and anti‐oxidant stress, inhibit macrophage proliferation	[216a]
		Naringenin (Nrg)‐loaded, folic acid‐coated lipid‐polymer nanoparticle (FA‐LNPs/Nrg)	128.2 nm ± 5.8nm(DLS)	Folic acid mediated stride over the intestinal mucosal‐epithelial barrier and target to folate receptor β on M1 macrophages	ApoE‐/‐ mice	Anti‐inflammatory, reduce the plaque burden	[217]
		Vascular endothelial growth factor/paclitaxel‐loaded porous micro/nanomotor	Micron level	Anti‐VCAM‐1 antibody target VCAM‐1 on endothelial cells	ApoE‐/‐ mice	Release VEGF and paclitaxel under near‐infrared light excitation, Promote endothelial proliferation, anti‐inflammatory	[218a]
		Anti‐interleukin‐1β (anti‐IL‐1β)‐loaded mesoporous silica nanoparticles MSNs @ anti‐IL‐1β	183.29 nm ± 17.16 nm	Passive target	ApoE‐/‐ mice	Protect vascular endothelial cells, inhibit VSMC proliferation and phenotype transformation, and improve local microenvironment.	[219]
		Curcumin‐loaded, HA‐coated mesoporous MnO_2_ nanoparticles	About 200 nm (TEM)	Hyaluronic acid (oHA) target CD44 on macrophage	ApoE‐/‐ mice	Promote M2 polarization, restore lipid efflux and remove lipids	[220]
		Rapamycin‐loaded UiO‐66‐NH‐FAM‐IL‐1Ra (RUFI)	About 100 nm	IL‐1Ra target macrophage	ApoE‐/‐ mice	Promote M2 polarization, induce autophagy, anti‐inflammatory and antioxidant stress	[221]
	Phototherapy	Mesoporous‐silica‐coated upconversion fluorescent nanoparticles encapsulating chlorin e6 (UCNPs‐Ce6)	/	Passive target	/	Induce macrophage apoptosis by mitochondrial caspase pathway	[222]
		Ultrasmall CuCo_2_S_4_ Nanocrystals	14 nm(TEM)	Passive target	ApoE‐/‐ mice	Photothermal ablate inflammatory macrophages	[227]
		CS‐CNCs@Ce6/DS	204.5 nm± 21.9nm(DLS)	Dextran sulfates target the type A scavenger receptor (SR‐A)	ApoE‐/‐ mice	Ablate Activated Macrophages	[112]
		PM‐PAAO‐UCNPs	Around 120 nm (DLS)	Platelet membrane target macrophage‐derived foam cells	ApoE‐/‐ mice	Reduce macrophage‐derived foam cells	[226]
		MoO_2_ nanoclusters	Around 40 nm (TEM), around 70 nm(DLS)	Platelet membrane target macrophage‐derived foam cells	ApoE‐/‐ mice	Ablate inflammatory macrophages	[228]
		Black TiO_2_‐hyaluronan‐porphine (bTiO_2_‐HA‐p)	211 nm ± 2 nm(DLS)	HA target CD44 on macrophages	/	Mildly induce foam cell apoptosis, promote cholesterol efflux, and reduce lipid intake	[232]
	SDT	5‐aminolevulinic acid(ALA)	/	Passive target	Adult male New Zealand white rabbits	Macrophage apoptosis and apoptotic cell clearance	[234]
		5‐aminolevulinic acid(ALA)	/	Passive target	New Zealand white rabbits and ApoE‐/‐ mice	Foam cell apoptosis and efferocytosis, cholesterol efflux	[235a]
		Sinoporphyrin sodium (DVDMS)	/	Passive target	New Zealand white rabbits and ApoE‐/‐ mice	Macrophage apoptosis‐induced endothelial cell apoptosis	[236]
		Sinoporphyrin sodium (DVDMS)	/	Passive target	New Zealand white rabbits and ApoE‐/‐ mice	Reduce iron retention in macrophages, inhibit the ferritin expression, treat intraplaque hemorrhage (IPH)	[237]
	Nanoimmuno therapy	TRAF6i–HDL	20.7 nm ± 3.0 nm	HDL target scavenger receptor type B‐1 (SR‐B1) on macrophages	ApoE‐/‐ mice	Block the interaction between CD40 and tumor necrosis factor receptor‐associated factor 6 (TRAF6); reduce monocyte migration	[243]
		PEI‐Au nanoparticles/shSiglec‐1/PEI‐ASA	92 nm(DLS)	Passive Targeting	ApoE‐/‐ mice	shSiglec‐1 blocks the recognition and interaction between macrophages and CD8^+^T cells and NKT cells ASA promotes cholesterol efflux, inhibits Th17 cells differentiation, and remodels immune microenvironment	[244]
		Simvastatin‐loaded high‐density lipoprotein (S‐HDL) nanoparticles	23.6 nm ± 3.7 nm	HDL target scavenger receptor type B‐1 (SR‐B1) on macrophages	ApoE‐/‐ mice	Clear excessive inflammatory cells, reduce plaque burden	[245]
	Gas therapy	NO‐delivering HDL‐like particles (SNO HDL NPs)	13.12 nm ± 0.65 nm (DLS)	HDL target scavenger receptor type B‐1 (SR‐B1) on macrophages and VSMCs	ApoE‐/‐ mice	Improve macrophage efflux cholesterol, inhibit aortic SMC migration, reduce inflammation.	[247]
		Dendrimer nanoplatform carrying 18 NO release units	/	/	/	Regulate NO release, maintain endothelial stability	[248]
		L‐arginine (LA) and lovastatin (LV)‐coloaded PLGA nanoparticle	114.0 nm ± 1.65 nm (DLS)	Passive target	ApoE‐/‐ mice	Improve the expression of eNOS and p‐eNOS, increase NO generation; deliver lovastatin	[249a]
		PLT membrane coated l‐arginine and γ‐Fe_2_O_3_ magnetic nanoparticles (PAMNs)	215.50 nm ± 8.05 nm (DLS)	Platelets membrane target damaged blood vessel	Ischemic stroke mice	Increase NO generation, cause vasodilation, disrupt the local PLT aggregation	[249b]
		Lipophilic nanomotor PMA‐TPP/PTX‐loaded with drug PTX and lipophilic triphenylphosphine (TPP)	About 200 nm (DLS)	Balloons located	High‐fat‐fed rabbits	NO nanomotor enhances drug penetration and retention, improve endothelial function, reduce ROS PTX inhibits intimal hyperplasia	[249c]
		Palladium hydride nanopocket cubes (PdH_0.12_ NPCs)	75.38 nm (DLS)	Passive target	ApoE‐/‐ mice	Hydrogen promotes cholesterol transport Pd reduces ROS and ox‐LDL generation	[251]
		Tetrapod needle‐like PdH nanoparticle delivered by living macrophages	Tetrapod: 100 nm the edge length: 60 nm	Living macrophages target to the inflammatory site	ApoE‐/‐ mice	Hydrogen reduces inflammation palladium removes ROS spike‐like structure promotes macrophage autophagy	[252]
		Trehalose‐L arginine‐phosphatidylserine (TAP) carrier‐free nanomotor	Around 200 (TEM)	Phosphatidylserine (PS) target macrophages in the AS	ApoE‐/‐ mice	L arginine produces NO drive into plaque trehalose promotes macrophages autophagy	[250]
Theranostic	MR imaging‐based	Rosuvastatin/Gd‐coloaded, HÀ‐coated hybrid liposomal cerasomes (CCs) named as (HA‐CC‐RST)	178 nm (DLS)	Hyaluronic acid (HA) target CD44 on macrophages	ApoE‐/‐ mice	Rosuvastatin‐mediated drug delivery therapy Gd mediated MR imaging	[274a]
		Glucagon‐like peptide‐1 receptor (GLP‐1R) agonists/gadolinium chelates‐coloaded nanoparticles (GlpNP)	53.7 nm	Passive target	ApoE‐/‐ mice	GLP‐1R promotes cholesterol efflux and stable plaque gadolinium chelates‐mediated MR imaging	[274b]
		Mito‐magneto (MM)‐loaded, stearyl‐TPP/stearyl mannose‐coated HDL‐mimicking nanoparticles	140–180 nm (DLS)	I) Mannose target to mannose receptors (MMR) on macrophages II) stearyl‐TPP target mitochondrion	BALB/c Albino mice	HDL‐mimicking‐mediated reverse cholesterol transport (RCT) mito‐magneto (based on iron oxide)‐mediated MR imaging	[275]
	FL imaging‐based	Mannose‐coated; lobeglitazone and Cy7‐loaded (MMR‐Lobe‐Cy)	207.2 nm ± 34.79 nm (DLS)	Mannose target to mannose receptors (MMR) on lipid‐overloaded macrophages	Rabbits	Lobeglitazone‐mediated rapidly anti‐inflammation Cy7‐mediated OCT‐NIFR imaging	[276b]
		Prednisolone (Pred)‐fluorophore (TP)‐loaded PMPC–PMEMA (PMM) nanoparticles (TPP@PMM)	57.5 nm ± 0.15 nm (DLS)	ROS responsive structures	ApoE‐/‐ mice	ROS‐responsive cleavage of the linker moiety releases prednisolone, which inhibits foam cell formation and reduces lipid uptake, leading to anti‐inflammatory effects. AIE fluorophore‐mediated two‐photon AIE bioimaging	[276c]
		(Ru(bpy)_3_@SiO_2_‐mSiO_2_@SRT1720@AntiCD36	225.3 nm (DLS)	AntiCD36 target CD36 on macrophage	ApoE‐/‐ mice	SRT1720 (SIRT1 activator) reduces the expression of proinflammatory factors, the number of foam cells, and promotes the reverse transport of cholesterol, Ru(bpy)_3_Cl_2_‐mediated FL imaging	[277]
		Upconversion nanoparticle (UCNP) and gold nanoparticles (AuNPs)‐coloaded chlorophyll‐modified liposomal	150‐250 nm	ROS responsive structures	/	Light excites chlorophyll, electrons are transferred to gold, and electrons on gold react with H^+^ to generate hydrogen to remove ROS. Förster resonance energy transfer (FRET)‐associated FL imaging reflects ROS levels.	[278]
		Hexyl 5‐aminolevulinate hydrochloride (HAL)‐loaded M2 macrophage‐derived exosomes (M2 Exo)	About 190 nm	M2 Exo have inherent inflammation tropism	ApoE‐/‐ mice	M2 Exo carries anti‐inflammatory factors that inhibits inflammation, the catabolic metabolites CO and bilirubin of HAL have anti‐inflammatory effects. The metabolic intermediate protoporphyrin IX (PpIX) of HAL has red fluorescence.	[279]
	US and MR imaging‐based	Fe_3_O_4_ (F)/ perfluorhexane (P)/DiR (D)‐loaded and dextran sulfate (D)‐coated PLGA‐PEG‐PLGA nanoparticles (FPD@CD)	255.9 nm ± 2.94 nm (DLS)	Dextran sulfate (DS) target SR‐A on macrophage	APOE‐/‐ mice	Perfluorohexane (PFH) under low‐intensity focused ultrasound (LIFU) promotes macrophage apotosis. Fe_3_O_4_‐mediated MR imaging, perfluorhexane‐mediated ultrasound, and DiR‐mediated near‐infrared fluorescence (NIRF) imaging	[280]
	US, PA, and MR imaging‐based	PFP‐HMME @PLGA/MnFe_2_O_4_‐ramucirumab nanoparticles (PHPMR NPs)	347.4 nm (DLS)	Anti‐VEGFR‐2 antibody target VEGF on endothelial cells	Plaque‐bearing rabbits	HMME‐mediated SDT induces neovessel endothelial cells apoptosis and improves hypoxia MnFe_2_O_4_‐mediated MR imaging, PFP mediated US and PA imaging	[98]
	PET imaging‐based	^89^Zr‐labeled hyaluronan nanoparticles (^89^Zr‐HA‐NPs)	32 nm ± 0.5 nm (AFM)	Hyaluronic acid (HA) target CD44 on macrophage	ApoE‐/‐ mice and New Zealand White rabbits	Hyaluronan‐mediated anti‐inflammation, ^89^Zr mediated PET imaging	[297a]
	US, FL, and PA imaging‐based	Indocyanine green‐loaded boronated maltodextrin (ICG‐BM) nanoparticles	About 500 nm (DLS and TEM)	Passive target	Ischemic mice	Hydrogen peroxide reacts with boronic ester in BM to generate an anti‐inflammatory drug 4‐hydroxybenzyl alcohol (HBA). Hydrogen peroxide degrades boronic ester and generates carbon dioxide bubbles to mediate US and PA imaging. Indocyanine green (ICG)‐mediated FL and PA imaging	[297b]
	CT imaging‐based	Gold nanorods	About 68 nm × 15 nm	Passive target	ApoE‐/‐ mice	Au‐mediated PTT ablation Au‐mediated CT imaging	[298]

### 4.1 Magnetic Resonance Imaging

MR imaging has been already used to diagnose coronary artery disease by utilizing nuclear magnetic resonance phenomena of certain atomic nuclei in tissues. Compared to other modalities, MR imaging has advantages like higher soft tissue resolution, deeper tissue penetration, and no ionizing radiation. MR imaging can offer partial information related to the thickness of the fiber cap, the size of the lipid necrosis core, and hemorrhage via different imaging sequences. However, low sensitivity, long operation time, and motion artifacts are the limitations of MR imaging in the diagnosis of atherosclerosis. The emergence of nanomaterials can assist in solving the above problems. Paramagnetic nanomaterials can fulfill precise positioning, improve sensitivity as well as safety, and obviously distinguish lesions from healthy tissue. Specifically, paramagnetic gadolinium (Gd)‐based nanoparticles and superparamagnetic iron oxide nanoparticles (SPIONs) are the most common MR imaging nanotracers in atheroscoerosis.^[^ ^135^ ^]^

On the one hand, Gd‐based MR contrast agents exhibit high signal on T1 weighting, which shortens the T1 relaxation time by affecting the relaxation rate of the surrounding water protons,^[^ ^136^ ^]^ but the toxicity of gadolinium ion limits its biological application.^[^ ^137^ ^]^ Therefore, Gd chelates have been prepared to improve their biocompatibility, for example, 1,4,7,10‐tetraazacyclododecane‐1,4,7,10‐tetraacetic acid (Gd‐DOTA) and diethylene triamine pentaacetic acid (Gd‐DTPA).^[^ ^138^ ^]^ Due to the specific accumulation of tropoelastin within the plaque, Gd_4_‐TESMA made up of four Gd (III)‐DOTA‐monoamide chelate conjugated with a tropoelastin‐binding peptide (the VVGS‐peptide) was introduced to illustrate the location and size of plaque through MR imaging tropoelastin.^[^ ^139^ ^]^ In another research, Gd‐DTPA was incorporated in the CNP, a complex nanoparticle self‐assembled by protamine peptide TPP1880 and low molecular weight fucoidan LMWF8875, to target P‐selectin overexpressed on the inflammatory endothelial cells surface.^[^ ^140^ ^]^

**Figure 4:**
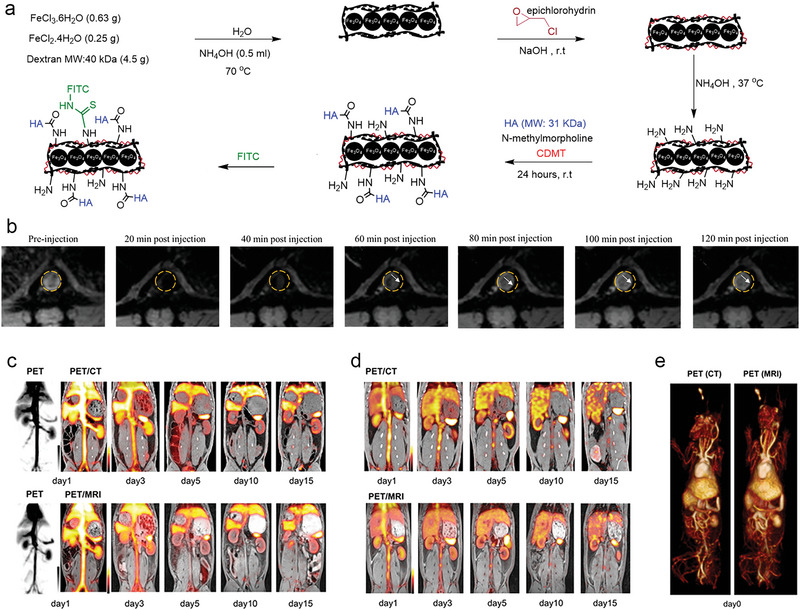
a) Schematic route illustration of the synthetic process of hyaluronan‐conjugated HA‐NWs. b) T2‐weighted MR images of the aorta at different time points after injection of HA‐NWs (8 mg Fe per kg body weight). a,b) Reproduced with permission.^[^ ^144^ ^]^ Copyright 2018, American Chemical Society. c) Biodistribution of ^89^Zr‐liposomes presented by PET and PET/CT (the upper chart) as well as PET and PET/MR imaging (the lower chart) in healthy rabbits at different time points after intravenous injection. d) Biodistribution of ^89^Zr‐liposomes presented by PET/CT (the upper) and PET/MR imaging (the lower) in atherosclerosis rabbits at different time points after intravenous injection. e) MIP from (c) PET/CT and (d) PET/MR imaging on day 0 after intravenous injection. c–e) Reproduced with permission.^[^ ^161^ ^]^ Copyright 2020, American Chemical Society.

On the other hand, iron oxide nanoparticles‐based MR contrast agents exhibit low signal on T2 weighting, which shortens T2 value by altering the local magnetic field strength and accelerating dephasing mediated by spin–spin effects. It is reported that very small iron oxide nanoparticles could be engulfed and enriched by mononuclear macrophages accumulating in inflammatory atherosclerosis.^[^ ^141^ ^]^ To further enhance the targeting function of SPIONs to plaque, OPN‐modified Fe_3_O_4_ nanoparticles (Cy5.5‐OPN‐DMSA‐MNPs, named as COD‐MNPs) were designed as MR imaging probe to indicate the lesions considering the overexpressed OPN on foamy macrophages.^[^ ^142^ ^]^ In another report, biomimetic nanomaterials were introduced to further improve the dispersibility and biosafety of SPIONs owing their live cell origin and the mutual recognition of cell surface markers.^[^ ^143^ ^]^ In addition, it reported that engineering the morphology of nanomaterials is significant to its targeting function. Hossaini Nasr and co‐workers constructed hyaluronan‐conjugated iron oxide nanoworms (named as HA‐NWs) with elongated shapes through the coprecipitation method to figure out the influence of the morphology of nanomaterials to MR imaging (**Figure** **4**a). Both in vitro and in vivo experiments suggested the higher uptake and lower inflammatory response of HA‐NWs than that of HA‐SPIONs. Besides, a series of T2 weighted MR images were presented via serial MR imaging, indicating the signal intensity of plaque reduced to 20% after 20 min compared with pre‐injection and kept its level until 120 min after injection (Figure 4b).^[^ ^144^ ^]^ Compared to the detection threshold of Gd‐based NPs, SPIONs owed better imaging sensitivity and biosafety with favorable magnetic property.^[^ ^145^ ^]^

### 4.2 Computed Tomography Imaging

CT imaging sketches a complete picture of the cardiovascular system at high speed and does not suffer from cardiac and respiratory artifacts. Additionally, the calcified component inherent in the plaque will appear as high‐density in CT images. Nevertheless, it is limited by ionization radiation and is difficult to detect the early lesions at the cellular level. Based on the photoelectric effect and the Compton effect, CT contrast agents commonly are elements with a large atomic number such as iodine (I), gold (Au), bromine (Br), barium (Ba), bismuth (Bi), tantalum (Ta), while most research on CT nanotracker in atherosclerosis pays attention to iodine and gold.

After intravenous injection, iodine will be rapidly cleared by the kidneys, resulting in a limited imaging time window. Therefore, higher dose needs to be injected in order to realize the desired effect. However, iodine is toxic when in direct contact with blood or tissue components. To address it, nanoemulsions,^[^ ^146^ ^]^ liposomal,^[^ ^147^ ^]^ dendrimers,^[^ ^148^ ^]^ polymeric NPs were prepared to weaken the direct contact between iodine and biological components.^[^ ^149^ ^]^ Extraordinarily, Hyafil et al. designed an iodinated nanotracer for CT imaging called N1177 to perform inflammatory macrophages imaging, which consists of ethyl‐3,5‐bis(acetylamino)‐2,4,6‐triiodobenzoate and surfactant.^[^ ^150^ ^]^ In another research, N1177 was used to identify ruptured plaques.^[^ ^151^ ^]^ The median CT value of ruptured plaques was 74 HU, which was approximately twice as high as that of unruptured plaques (32 HU).

AuNPs have the advantages of controlled shape and size, high biocompatibility, simple surface modification, and diversified functions.^[^ ^152^ ^]^ According to the thrombosis at the local site of atherosclerotic plaque and thromboembolism at distant sites due to plaque rupture, fibrin‐targeting peptides‐loaded AuNPs were developed to achieve the visualization of thrombus composition and the assessment of thrombolytic effect with tissue plasminogen activator via CT imaging.^[^ ^153^ ^]^ Besides, as monocytes play an important role in atherosclerotic pathology, Chhour et al. first used AuNPs‐labeled monocytes for tracing their migration and recruitment from blood to atherosclerotic plaques by non‐invasive CT imaging.^[^ ^154^ ^]^ It examined the effect of surface modification of AuNPs on monocytes with different ligands and demonstrated that AuNPs with small hydrodynamic diameters and negative zeta potentials were more accessible to monocytes. In another research, AuNPs were utilized in combination with photon‐counting CT k‐edge imaging,^[^ ^155^ ^]^ a technique capable of analyzing the photon energy, reclassifying it to fulfill high spatial resolution imaging, and preventing confusion from calcifications in conventional CT images.^[^ ^156^ ^]^ The researchers adopted an iodinated contrast agent to reflect the lumen structure and AuNPs to indicate the macrophage burden in the vessel wall to simultaneously achieve anatomic and molecular CT imaging.

### 4.3 Radionuclide Imaging

Radionuclides or radionuclide labeled substances are introduced into the body to participate in tissue metabolism, emitting nuclear rays that can penetrate tissues and be detected noninvasively by radionuclide imaging from the body surface to reflect specific pathophysiological processes. The radionuclide imaging modalities can be divided into two types: positron emission tomography (PET) and single‐photon emission CT (SPECT) depending on the type of radionuclide. Radionuclide imaging allows extremely high detection sensitivity for metabolism, which belongs to functional imaging. However, the disadvantages include low anatomical resolution and the interference from surrounding tissues, hence it is generally applied in fusion with MR or CT imaging to improve anatomical background.

As for PET, NaF could bind to hydroxyapatite in calcification,^[^ ^157^ ^]^ and a previous study verified the significant accumulation of ^18^F‐NaF in plaque calcification using Yucatan minipigs as a model.^[^ ^158^ ^]^ Their results confirmed that ^18^F‐NaF‐mediated PET imaging is a valid detection tool for identifying atherosclerotic calcification. Besides, radionuclides can directly label the corresponding antibodies, peptides, or ligands for selective tracing of a particular pathological process with high accuracy.^[^ ^159^ ^]^ What's more, metal positronium nuclides such as ^89^Zr, ^64^Cu, ^99m^Tc, ^68^Ga, etc. with longer half‐life time have gradually attracted more attention in recent years.^[^ ^160^ ^]^ In a report, Lobatto et al. structured ^89^Zr radiolabeled liposomes chelated with deferoxamine B, which provided new perspectives related to synergistic drug delivery therapy.^[^ ^161^ ^]^ It was observed that the blood pool and vascular‐rich organs were first filled, and over time, the liposomes in the blood pool were progressively cleared and the mononuclear phagocyte system was progressively brightened in healthy rabbits (Figure 4c). Then ^89^Zr‐labeled liposomes were injected into model rabbits, and when the contrast agent in the normal blood pool subsided, patchy atherosclerosis plaques could be clearly shown on PET/CT (Figure 4d). PET maximum intensity projections (MIP) offered a more stereoscopic view of the biodistribution of the ^89^Zr‐labeled liposomes (Figure 4e). As for ^64^Cu, here is another experimentation, Detering and co‐workers constructed CCR5‐targeted amphiphilic comb copolymers, denoted as D‐Ala‐peptide T‐amide (DAPTA‐Comb), which were synthesized and matched with ^64^Cu as radiolabel with favorable half‐life.^[^ ^162^ ^]^ By changing the ratio of various copolymers, the physicochemical state and surface function were precisely controlled.

Regarding SPECT, Liang and co‐workers conducted an intriguing study in which they isolated and purified natural human H‐ferritin nanocages from *Escherichia coli*.^[^ ^163^ ^]^ Chelation of ^99m^Tc with H‐ferritin in the presence of N‐hydroxysuccinimide ester MAG3 conferred H‐ferritin the ability to be imaged via SPECT/CT for the quantitative analysis of vulnerable plaque. Signal intensity analysis was in good agreement with the plaque area revealed by oil red O staining of the isolated arteries. Gamma imaging of the isolated arteries was performed to further verify the presence of plaque much more clearly. In addition, the high contrast and low background signal of ^99m^Tc‐ferritin allowed them not only to indicate the progression of atherosclerotic plaques but also to be employed as a sensitive tool to monitor the anti‐inflammatory effects.

### 4.4 Fluorescence Imaging

FL imaging, an emergent imaging technique for noninvasive visualization, is used for bioanalysis, disease monitoring and drug distribution thanks to its high sensitivity, low cost, and rapid acquisition and processing of images. It relies on optical materials with light absorption capabilities, especially NIR fluorescent materials with deeper imaging capabilities and higher signal‐to‐noise ratio. Organic small molecule fluorescent dyes (within a few kDa) such as rhodamine, coumarin,^[^ ^164^ ^]^ cyanine,^[^ ^165^ ^]^ naphthimide,^[^ ^166^ ^]^ BODIPY,^[^ ^167^ ^]^ pyrene,^[^ ^168^ ^]^ indocyanine green (ICG),^[^ ^169^ ^]^ etc. with superior optical properties are already used and make a contribution to multimodality imaging. Additionally, the development of nanomaterials offers new possibilities for FL detection of atherosclerosis. At present, there are three feasible approaches including (I) autofluorescence of tissues, (II) nanomodification of fluorescent dyes, or (III) the construction of fluorescent nanoprobes.

**Figure 5:**
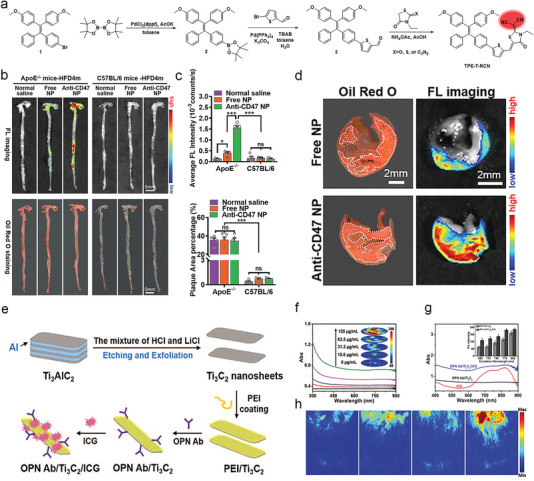
a) Preparation route of TPE‐T‐RCN. b) Typical fluorescence images and Oil Red O staining images of the isolated aorta from HFD‐fed 4 months ApoE−/− mice and HFD‐fed 4 months C57BL/6 mice under different treatments (intravenous inject normal saline, free NP, anti‐CD47 NP). c) Average fluorescence intensity value (upper) and the percentage of plaque area (lower) of the isolated aortas in every group. *P < 0.05, ***P < 0.001 and ns: not significant. d) Representative Oil Red O staining images and fluorescence images of human fresh carotid atherectomy specimens after different treatments (incubate with free NPs or anti‐CD47 NPs for 2 h). a–d) Reproduced with permission.^[^ ^173^ ^]^ Copyright 2022, Wiley‐VCH. e) Synthetic route to OPN Ab/Ti_3_C_2_/ICG. f) UV–vis absorption spectra and PA images under excitation wavelengths of 808 nm of Ti_3_C_2_ nanosheet (insert picture) with different concentrations. g) Absorption spectra before and after loading ICG as well as the distinguishable PA intensity of OPN Ab/Ti_3_C_2_ and OPN Ab/Ti_3_C_2_/ICG at different excitation wavelengths (inset picture). h) In vivo PA imaging of mice. Mice were divided into four groups: I) atherosclerotic mice without any treatment, II) atherosclerotic mice i.v. with Ti_3_C_2_/ICG, III) healthy mice i.v. with OPN Ab/Ti_3_C_2_/ICG, IV) atherosclerosis mice i.v. with OPN Ab/Ti_3_C_2_/ICG. e–h) Reproduced with permission.^[^ ^120^ ^]^ Copyright 2020, Wiley‐VCH.

First, the autofluorescence properties of endogenous complexes such as some lipid components (e.g., insoluble lipid or ceroid), ox‐LDL, collagen, heme, and its metabolites bilirubin can be employed to reflect compositional changes at different stages of atherosclerosis.^[^ ^170^ ^]^ Besides, functional group substitutions, surface modification, encapsulation, or optimization of ligands are able to retain intrinsic properties of FL agents while also improving their bio‐imaging applications.^[^ ^171^ ^]^ Recently, Narita et al. synthesized a cleavable peptide‐ICG2 and encapsulated it into PS‐loaded liposomes, which actively drive them to macrophages in embolism‐vulnerable plaques.^[^ ^172^ ^]^ These nanotracers undergone FL quenching at the existence of peptide linker in normal conditions. After taken up by macrophages, lysosomal enzyme and cathepsin B cleaved the peptide linker and terminated FL quenching, as a result, the fluorescence signaling was detectable in the deeper tissue. Another attractive research reported a dicyanomethylene‐substituted rhodamine derivative (TPE‐T‐RCN), an aggregation‐induced emission (AIE) nanoluminogen to perform atherosclerotic FL imaging (**Figure** 5).^[^ ^173^ ^]^ 3D geometric structures confirmed that it was a D–π–A type AIE luminogen with tetraphenylethene (TPE) as the electron donor group, dicyanomethylene unit (CN) as the electron acceptor group, and thiophene (T) as the π‐conjugate to achieve the optimal photoluminescence quantum yield. TPE‐T‐RCN was encapsulated with DSPE‐PEG and coupled with CD47 (a “do not eat me” signal) antibody to form the final FL nanoprobe. After 12 h injection of FL nanoprobe, the aorta of the experimental and control mice was excised intactly and performed by FL imaging and oil red O staining. The plaque areas in the aorta of the atherosclerotic mice were almost identical, whereas the imaging capabilities were totally distinct. What is more, there was a significant correspondence between FL signal and plaque site in anti‐CD47 NPs treated group (Figure 5b‐d). Significant accumulation of FL nanoprobe at the plaque site was observed with immunofluorescence imaging, which was hardly visible in the control group.

Additionally, nanomaterials with applications in FL imaging include QDs, AuNPs, carbon‐based nanomaterials, upconversion nanoparticles (UCNPs), etc.^[^ ^174^ ^]^ Wherein, QDs perhaps are the most critical nanomaterials, which possess long FL lifetime.^[^ ^175^ ^]^ PbS/CdS/ZnS QDs were introduced to identify the ischemic phase from the inflammatory phase due to their unique FL brightness and thermal sensitivity.^[^ ^176^ ^]^ Besides, Deng et al. first constructed dextran‐mimetic and HgCdSe‐based QD probes (Q‐Dex) to point the atherosclerotic area.^[^ ^177^ ^]^

### 4.5 Photoacoustic Imaging

PA imaging combines the high resolution of optical imaging with the deep penetration of acoustic imaging. Principally, thermal expansion caused by absorbers under laser light generates acoustic waves. PA imaging can detect acoustic waves in tissue at a depth of several centimeters and overcome the “soft limits” of optical depth.^[^ ^178^ ^]^ Research in PA imaging focus on preclinical study and aims to optimize imaging systems as well as algorithms and explores more suitable absorbers. Intravascular photoacoustic (IVPA), a tool for plaque components imaging, has been used to identify endogenous absorbers such as lipid, haemoglobin and collagen. In particular, the chronic deposition of lipid with its characteristic absorption peaks at 1210 nm in atherosclerotic plaques could be selected as a biomarker to indicate the accumulation of foam cells and the size of lipid core within plaque.^[^ ^179^ ^]^ Not only lipid core size, Cao et al. further analyze lipid composition to achieve tissue‐type characterization via IVPA.^[^ ^180^ ^]^

A safer option is screening the lesion from the body surface directly, but exogenous absorbers must be introduced to solve the weakened penetration problem caused by increased detection distance. For displaying inflammatory cells within the plaque, Ge et al. constructed a PA contrast agent based on NIR fluorescence probe ICG and osteopontin antibody (OPN Ab) co‐loaded Ti_3_C_2_ nanosheet (OPN Ab/Ti_3_C_2_/ICG) to target inflammatory foam cells and distinguish the vulnerable atherosclerosis plaque (Figure 5e).^[^ ^120^ ^]^ The nanosheet had obvious absorption at 808 nm in a concentration‐dependent manner, and the loading of ICG further enhanced its PA performance (Figure 5f,g). In vivo experiments demonstrated that OPN Ab/Ti3C2/ICG nanosheet could develop as a promising candidate which particularly accumulated in atherosclerotic plaque and achieved noninvasive PA imaging at the molecule level (Figure 5h). In previous research, Smith et al. found that single‐walled carbon nanotubes (SWNTs) could specifically target the Ly‐6C^hi^ monocytes subpopulation in circulating blood cells.^[^ ^181^ ^]^ Inspired by it, PEG‐coated SWNTs with long circulation times and favorable photothermal conversion efficiency were presented by the same group to detect the inflamed arterial plaque.^[^ ^111^ ^]^ Similar to this idea, Xie et al. investigated the PA performance of PBD, a semiconductor nanomaterial with absorbance in the NIR II, fulfilling a huge hint of resolution.^[^ ^182^ ^]^ In this report, PEG was selected to improve the water solubility of PBD while acting as a bridge to CD36 antibodies, facilitating specific PA imaging of foam cells. In addition, a novel ratiometric semiconducting polymer nanoparticle (RSPN) was proposed to reflect the oxidative stress level with an internal PA reference in ApoE‐/‐ mice complicated with pneumonia from normal mice, which revealed significant potential for detecting vulnerable plaque via PA imaging.^[^ ^183^ ^]^

### 4.6 Optical Coherence Tomography (OCT)

OCT detects the reflection, scattering, and other signals of biological tissues by utilizing their light transmission properties, which are then converted into electrical signals and reconstructed into images through computer analysis. However, due to the inherent limitations of light penetration, it is difficult to detect atherosclerotic plaques from the body surface, making endovascular imaging necessary. Intravascular OCT is a multifunctional and high‐resolution clinical tool that helps identify the morphology of blood vessels and plaques, their internal components, and plaque vulnerability, and can contribute to investigating the mechanisms of plaque erosion and healing.^[^ ^184^ ^]^ However, as the probe is placed in the bloodstream, the image is distorted by red blood cells, larger plaques, and thrombotic lesions.

Optical nanoprobes that target specific molecules expressed during different stages of plaque development can be beneficial in improving the detection sensitivity of intravascular OCT. By attaching to these specific molecules, these probes can provide contrast and enhance the visualization of plaque components during imaging. There have not been many studies on nanomaterials in this area to date. Au nanoparticles are crucial in providing OCT signals due to their strong backscattering cross‐section.^[^ ^185^ ^]^ Gold nanoshells, excited by OCT at 1300 nm, produced clear images of individual cells, which had been validated in both suspended and adherent cells.^[^ ^186^ ^]^ Another study involved infrared emitting quantum dots (IR‐QDs), which also produced sufficient backscattering when excited by a single‐line laser OCT at 1300 nm. According to theoretical calculations, the scattering cross‐section of a single IR‐QD is nine orders of magnitude smaller than that of gold nanoshells, which may be due to their significant differences in volume. However, at equal mass concentrations, the OCT intensity of both was almost the same, and IR‐QDs re‐emitted light at 1600 nm, which could be used for additional FL imaging.^[^ ^187^ ^]^ From a clinical perspective, the next step is to give these nanomaterials targeting abilities so that specific component imaging of atherosclerosis can be unlocked.

### 4.7 Multimodal Imaging

The various single imaging methods have significantly contributed to the atherosclerotic diagnosis, nevertheless, their intrinsic limitations make it difficult to meet the requirements of resolution, sensitivity, and selectivity at the same time. Multimodal imaging permits us to compensate for shortcomings, achieve complementary strengths, and obtain much accurate information at the early diagnosis stage of the disease. Multimodal imaging, whether fusion imaging (one machine for multiple technologies) like PET/CT, SPECT/CT and PA imaging or the combined application of multiple imaging techniques, relies on imaging probes as vehicles. The small size, large specific surface area, high payload, and intelligent design of the multimodal imaging nanoprobes allow each probe to carry more substances and information for the detection by multimodal techniques.

Previously, zinc‐doped ferrite nanocomplex was fabricated to improve the spatial resolution of MR imaging and achieve MR/CT bimodal imaging, which allowed stronger negative MR contrast and lower toxicity in comparison with commercial ferrite nanoparticles.^[^ ^188^ ^]^ For dual‐mode imaging, although MR imaging has already provided high anatomical resolution, it still requires the assistance of a high‐sensitivity device. Regarding PEG as a bridge, a near‐infrared fluorochromes‐carried and ligand‐attached SPION was designed to target atherosclerotic activated platelets, which achieved the superimposition of two imaging elements.^[^ ^189^ ^]^ Similarly, Wu et al. prepared the mesoporous silica layer‐coated, NIRF dye (IR820)‐loaded and PP1 (a foamy macrophage‐targeted peptide)‐coupled SPION, noted as PP1‐IO@MS‐IR820 (PIMI), to perform MR/FL bimodal imaging.^[^ ^190^ ^]^

As for multimodal imaging, to evaluate the inflow of immune cells, Senders et al. constructed a nanoprobe with bone marrow cell specificity to perform immunokinetic studies.^[^ ^81^ ^]^ They innovatively wrapped the perfluoro‐crown ether core with the HDL derivative ApoA‐1 and phospholipids, abbreviated as ^19^F‐HDL, followed by loading ^89^Zr and cholesteryl BODIPY to perform ^19^F MR imaging. The half‐life time of ^89^Zr is 78.4 h, only permitted high‐sensitivity monitoring for 3 days in vivo, while the join of fluorine core allowed quantitative study for up to 28 d after a single injection via ^19^F MR imaging. Gamma imaging and optical techniques such as flow cytometry were performed additionally to study cell subpopulations ex vivo. Besides, ^19^F MR imaging and flow cytometry indicated that the intraplaque inflammatory cells were mainly Ly6C^high^ monocytes recruited from the bone marrow and spleen in the intermediate stage of atherosclerosis, whereas were mainly the local proliferation of macrophages in the advanced stage. Additionally, the novel multimodal imaging probe 5‐HT‐Fe_3_O_4_‐Cy7 nanoparticles (5HFeC NPs) via FL/CT angiography/magnetic particle imaging (MPI) was constructed for the detection of excessive active MPO and inflammatory conditions within vulnerable plaque.^[^ ^191^ ^]^ Regarding SPIONs as the contrast agent, MPI (a novel tomographic method) greatly reduced the dose of SPIONs due to their high sensitivity. However, high sensitivity is often accompanied by the loss of spatial resolution, so CT angiography was selected as the contributor to provide anatomical information. What's more, the zero‐signal attenuation of MPI compensated for the problem caused by the limited penetration depth of FL imaging.

### 4.8 Clinical Studies of Atherosclerosis Diagnosis in Nanomedicine

There are some reports on the clinical research of ultra‐small superparamagnetic iron oxide (USPIO) in atherosclerosis. Initially, the first‐generation FDA‐approved USPIO contrast agent Ferumoxtran (Sinerem) was designed for lymph node and tumor imaging.^[^ ^192^ ^]^ Subsequently, researchers discovered signal loss in atherosclerotic plaques, suggesting its potential for atherosclerosis imaging and exploration.^[^ ^193^ ^]^ However, in 2010, its application was restricted due to liver function damage and was subsequently withdrawn from the market.

The second‐generation USPIO contrast agent Ferumoxytol (Feraheme) was originally intended as an iron replacement therapy for treating iron deficiency anemia. Ferumoxytol consists of iron oxide nanoparticle core coated with carboxymethyl dextran.^[^ ^194^ ^]^ In atherosclerotic research, it gained attention due to its ability to accumulate in inflammatory tissue surrounding atherosclerotic plaques, possibly due to a specific mechanism related to plaque permeability.^[^ ^195^ ^]^ The optimal time for evaluating atherosclerosis was found to be 48 h after injection.^[^ ^196^ ^]^ Additionally, Ferumoxytol can be used as a non‐invasive imaging method for diagnosing diseases related to coronary artery disease, vascular inflammation, and heart disease and peripheral artery disease (PAD).^[^ ^197^ ^]^

Overall, for the diagnosis of atherosclerosis, MR imaging plays a crucial role, often utilizes iron oxide nanoparticles as a core and obtains certain results in clinical trials. CT imaging relies on elements with high atomic numbers, with gold nanoparticles as a typical representative, constructing different forms. FL imaging is diverse in design, but always centers around certain endogenous metabolites, various modified/unmodified small molecule fluorescent dyes, and materials platforms with fluorescent emission properties such as UCNPs and quantum dots. Nuclear medicine imaging relies on labeled radioactive isotopes, while photoacoustic imaging depends on intrinsic absorption differences of biological components or material systems with absorption capacity in the NIR. It is noteworthy that US imaging is still a diagnostic system with a lot of exploration space in the nanomedicine field for atherosclerosis. The above‐mentioned imaging methods have their own strengths and weaknesses, and sometimes it is difficult to fulfill synchronous improvement of sensitivity and resolution. Therefore, the multi‐modal imaging mode for atherosclerosis has emerged to make up for the shortcomings of the single imaging methods and contributes to obtain more comprehensive and accurate information. The multimodal imaging modalities are constantly developing and innovating by preparing diverse multimodal nanoprobes. The targeting molecules and templates mentioned above also can be further designed for atherosclerotic treatment.

## 5 Therapy

The BMT for atherosclerosis includes lowering blood lipids, anti‐hypertension, anti‐thrombosis, and anti‐platelet treatment, which needs to be taken for the whole life and may cause systemic side effects, such as liver and kidney damage. If the lesion is advanced and leads to severe vascular occlusion, surgical intervention is required (e.g., stent implantation, intraluminal endarterectomy, or bypass surgery). Although the above methods have achieved certain outcomes in clinical practice, it is still urgent to develop new treatment strategies to optimize the existing treatment protocol. Nanotechnology‐based therapeutic systems are used to finely regulate specific stages of atherosclerotic development. In this section, we mainly discuss the potential application of nanosystems in the improvement of atherosclerosis including drug delivery, phototherapy, SDT, immunotherapy, and gas therapy (Table 2).

### 5.1 Drug Delivery

To further reduce the systemic toxicity of drugs and increase their circulation time in the body, researchers innovatively introduce nanocarriers to specifically deliver drugs into the targeted lesions. Afterward, the research scope extended from traditional drugs to various small molecule regulators, proteins, cytokines, and gene components. Besides, nanocarriers have gradually expanded to liposomes,^[^ ^198^ ^]^ biomimetic nanomaterials,^[^ ^199^ ^]^ polymers,^[^ ^26a^ ^]^ micelles,^[^ ^200^ ^]^ mesoporous structures,^[^ ^201^ ^]^ and metal‐organic framework (MOFs).^[^ ^202^ ^]^

Liposomes have been widely used for the delivery of nanomedicines in various fields due to their nontoxic structures, excellent biocompatibility, easy biodegradation and the protective function of drug.^[^ ^203^ ^]^ Statins have the ability to regulate lipids, combat inflammation, and stabilize plaque, but they are lipid‐soluble drugs that cannot be directly injected into veins and the long‐term oral administration of statins will impair liver function. Therefore, the group developed a stable simvastatin (STAT)‐loaded liposome and verified the excellent ability of the liposome to promote cholesterol efflux and its anti‐inflammatory properties.^[^ ^204^ ^]^ Besides statins, Benne et al. prepared LXR agonists‐loaded and cyclic peptide Lyp‐1‐modified liposomes in which Lyp‐1 can target the gC1q receptor on foam cell surfaces, and the encapsulation efficiency is close to 100%. After internalization, the released LXR agonists upregulated the expression level of ABCA1 and ABCG1, resulting in a significant reduction in plaque burden and inflammatory macrophages.^[^ ^205^ ^]^ In addition, liposomes have also been utilized for targeted delivery of gene regulators such as NLRP3 siRNA which inhibits the formation of inflammasomes and miRNA‐146a which restricts NF‐κB inflammation pathway activation.^[^ ^206^ ^]^ Interestingly, Wu et al. designed an apoptotic body biomimetic nanoliposome (AP‐Lipo) to deliver drug to the atherosclerotic macrophages with maximum efficiency to reduce plaque burden.^[^ ^207^ ^]^ PS and cRGDfK peptide‐coupled DSPE‐PEG were inserted into PPARγ agonist pioglitazone (PIO)‐loaded liposomes, in which PS emitted “eat me” signal and promoted the phagocytosis of inflammatory macrophages. The experimental results confirmed that AP‐Lipo significantly induced the phenotypic transition of macrophages from M1 to M2, increased the proportion of collagen, and stabilized the plaque.

**Figure 6:**
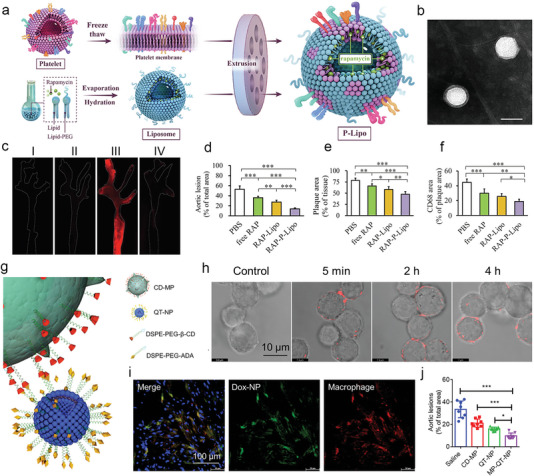
a) Illustration for the preparation of RAP‐P‐Lipo. b) TEM image of RAP‐P‐Lipo. c) Fluorescent images of the isolated aorta after different treatments (red: DiD‐labeled liposomes, I: PBS group, II: Lipo group, III: P‐Lipo group, IV: P‐Lipo+C57 Mice group). d) Quantitative analysis of plaque area as a percentage of the total aortic area. Quantitative analysis of e) the plaque area and f) macrophage content in sections of the aortic valve (*n* = 6). *p* < 0.05, ** *p* < 0.01, and *** *p* < 0.001. a–f) Reproduced with permission.^[^ ^209^ ^]^ Copyright 2023, Elsevier. g) Schematic diagram of the macrophage‐liposome conjugate. h) Confocal laser scanning microscope (CLSM) after co‐incubation of macrophages with rhodamine‐loaded liposomes at different times. i) FL imaging of aortic slices (blue: DAPI; green: Dox‐loaded liposomes; red: DiD‐labeled macrophages). j) Quantitative analysis of plaque area of total area. *P ≤ 0.05, **P ≤ 0.01, and ***P ≤ 0.001. g–j) Reproduced with permission.^[^ ^214^ ^]^ Copyright 2022, Elsevier.

Biomimetic nanoparticles retain partial basic structures and characteristics of cells, which can be used to wrap nanoparticles, increase biocompatibility, reduce immunogenicity, and improve targeting ability. Considering the inherent adhesion ability of platelets to damaged endothelium, a platelet membrane‐coated PLGA nano‐delivery platform loaded with rapamycin (RAP‐PNP) was designed to target plaques with high drug loading rate up to 3.55 ± 0.06%. Compared with free rapamycin, RAP‐PNP further reduced the area of plaques.^[^ ^208^ ^]^ In vitro, the binding capacity of RAP‐PNP conferred by platelet membranes was verified in static conditions and flow‐chamber model, respectively, and the uptake of RAP‐PNP by foam cells was significantly enhanced. Considering that the extraction of platelet membrane requires a large supply of platelets, a new strategy of fusing platelets with artificial liposomes was proposed, which greatly improved the utilization of platelets (**Figure** **6**a,b).^[^ ^209^ ^]^ In vivo, rapamycin‐loaded and platelet membrane‐fused liposomes (RAP‐P‐Lipo) were successfully accumulated in the plaques of the atherosclerotic model mice, which was not observed in free liposomes and was negligible in healthy C57 mice (Figure 6c). Histological sections of the aortic valve also showed that the fused structure had a highly co‐localized distribution with collagen, activated endothelial cells, and macrophages. After eight weeks of treatment, the aortas of mice in each group were obtained for analysis. The plaque area was significantly reduced, and the burden of macrophages was relieved, which had statistical significance both in the gross staining by Oil Red O and in the histological sections (Figure 6d–f). Compared with platelets, the inherent inflammatory chemotactic ability of macrophages has also attracted widespread attention.^[^ ^210^ ^]^ Sha et al. encapsulated SHP_1_ inhibitor (SHP1i) into liposome nanoparticles with macrophage membrane layer, and the membrane structure could competitively bind to ox‐LDL and lipopolysaccharide, thereby reducing anti‐inflammatory factors, ROS, and iNOS production. The loaded SHP_1_i blocked the CD47‐SIRPα “don't eat me” signaling pathway, promoting macrophages to engulf apoptotic cells (efferocytosis).^[^ ^211^ ^]^ In another report, CD47 plasmid was transfected into macrophages to express a large amount of anti‐phagocytosis protein CD47 to effectively evade phagocytosis by the reticuloendothelial system. At the same time, endothelin‐1 was used to stimulate macrophages to overexpress integrins α4/β1 and selectively bind to VCAM‐1 on the surface of endothelial cells, delivering potent anti‐inflammatory drug colchicine and providing a new strategy for the application of biomimetic cell membranes.^[^ ^212^ ^]^

Cell‐mediated drug delivery aims at using the inherent homing or chemotactic abilities of live cells to carry nanodrugs to the site of lesions. A simvastatin acid‐loaded dendritic polymer attached to red blood cells (SA PAM@RBCs) was constructed with 95.8 ± 3.1% encapsulation rate and 65.3 ± 2.1% drug loading rate, which responded to local shear stress and ROS. Briefly, the drug entered the bloodstream with red blood cells and reached the atherosclerotic site. Under the local high shear stress, the dendritic polymer detached from the surface of the red blood cells, and a large amount of ROS promoted SA PAM protonation to further release SA.^[^ ^213^ ^]^ In another report, a macrophage‐liposome living cell hitchhiking strategy was proposed innovatively to achieve precise delivery of β‐cyclodextrin and quercetin.^[^ ^214^ ^]^ The host–guest binding of β‐cyclodextrin to adamantane mediated the attachment of quercetin‐loaded liposomes to the surface of macrophage (Figure 6g). Then the macrophage‐liposome conjugate entered the plaque depending on the intrinsic inflammatory chemotaxis of macrophages and released quercetin. The research indicated that β‐cyclodextrin was stably anchored to macrophages within 8 h and mediated the binding of macrophages and liposomes within 4 h via the reacting with adamantane (Figure 6h). After injection into mice, the macrophage‐liposome conjugate showed a significant colocalization with macrophages, indicating excellent targeting ability for drug delivery (Figure 6i). Quercetin, as an antioxidant stress drug, could activate the NRF2 pathway, reduce local ROS levels and inhibit plaque inflammation. In addition, the β‐CD anchored on macrophages could dissolve cholesterol and increase lipid efflux. The synergistic effect of quercetin and β‐cyclodextrin reduced the proportion of the area of aortic plaques from 34.6% to 8.6% (Figure 6j).

Polymeric nanoparticles have been widely used as promising candidates for drug delivery due to their variable size, easy surface modification, and controllable drug release. Li et al. established cRGD‐modified polymeric nanoparticles that specifically deliver angiogenesis inhibitors pigment epithelium‐derived factor (PEDF) with 91.7 ± 2.6% entrapment efficiency to endothelial cells to stabilize plaques.^[^ ^215^ ^]^ In addition, the multiple sites on the surface of the polymer make it easy to construct intelligent responsive delivery systems, which can stably and continuously release loaded drugs under stimulation, thereby effectively reducing off‐target effects.^[^ ^216^ ^]^ Furthermore, folic acid‐modified lipid‐polymeric nanoparticles encapsulating naringenin (Nrg) were found to effectively overcome the intestinal mucosal barrier and accumulate in atherosclerotic macrophages to exert an anti‐inflammatory effect.^[^ ^217^ ^]^

In addition to the commonly used drug delivery platforms mentioned above, other nanosystems, such as mesoporous systems, MOFs, tubular systems, and metal nanoparticles have also been elaborated for atherosclerotic drug delivery in recent years.^[^ ^218^ ^]^ For example, Pu et al. designed a mesoporous silica system with a pore size of 15.5 nm to deliver anti‐IL‐1β, which effectively protected blood vessels, and inhibited VSMC proliferation and inflammatory phenotype transformation.^[^ ^219^ ^]^ Manganese dioxide (MnO_2_) was also developed as a mesoporous structure and added with metal coordination to deliver curcumin, achieving a loading efficiency of up to 54%.^[^ ^220^ ^]^ Additionally, a MOFs structure based on UiO‐66 (named RUFI) was constructed to co‐load rapamycin and interleukin‐1 receptor antagonists, achieving synergistic therapeutic effects. They regulated macrophage phenotype, promoted autophagy, and enhanced the immune regulatory function against atherosclerosis.^[^ ^221^ ^]^

### 5.2 Phototherapy

Phototherapy refers to the process of generating ROS or heating up under the excitation of the light at a specific wavelength, converting light energy into chemical or thermal energy to kill specific cells (e.g., macrophages, and endothelium). For the treatment of atherosclerosis, phototherapy mainly includes photodynamic therapy (PDT) and photothermal therapy (PTT).

PDT usually refers to the process of promoting the generation of ROS and inducing chemical damage by photosensitizers under light irradiation.^[^ ^222^ ^]^ The previous reports included Ce6‐mediated foam cell autophagy and cholesterol efflux,^[^ ^223^ ^]^ IR780‐mediated inflammatory cell ablation,^[^ ^224^ ^]^ and curcumin‐induced VSMC autophagy.^[^ ^225^ ^]^ To solve the problem of weak penetration ability of light, Ma et al. incorporated Ce6 into micelles together with UCNPs to handle atherosclerosis, which activated Ce6 to produce ROS at 980 nm excitation wavelength. Considering the affinity of platelets to early atherosclerotic plaques, the nanoparticles were further coated with platelet membranes, named as PM‐PAAO‐UCNPs. The in vitro experiments demonstrated that UCNPs conferred superior tissue penetration to PM‐PAAO‐UCNPs while generating considerable ROS. The in vivo experiments revealed that PM‐PAAO‐UCNPs‐mediated PDT (980 nm, 10 mW, 30 min) significantly reduced plaque volume and lipid area, decreased the secretion of pro‐inflammatory factors (IL‐6 and TNF‐𝛼) and promoted the secretion of anti‐inflammatory factors (TGF‐𝛽 and IL‐10).^[^ ^226^ ^]^

Nanomaterials^[^ ^227^ ^]^ with high photothermal conversion efficiency can be used to perform PTT, which is the process of converting light energy into heat energy and inducing physical damage to eliminate specific pathological components.^[^ ^228^ ^]^ Peng et al. designed a ternary semiconductor with up to 43% photothermal conversion efficiency based on theoretical calculations, achieving good macrophage ablation effects.^[^ ^229^ ^]^ In addition, the light‐responsive thermal effect generated a thermophoresis phenomenon, promoting the movement of nanomaterials and drug release.^[^ ^218^, ^230^ ^]^

**Figure 7:**
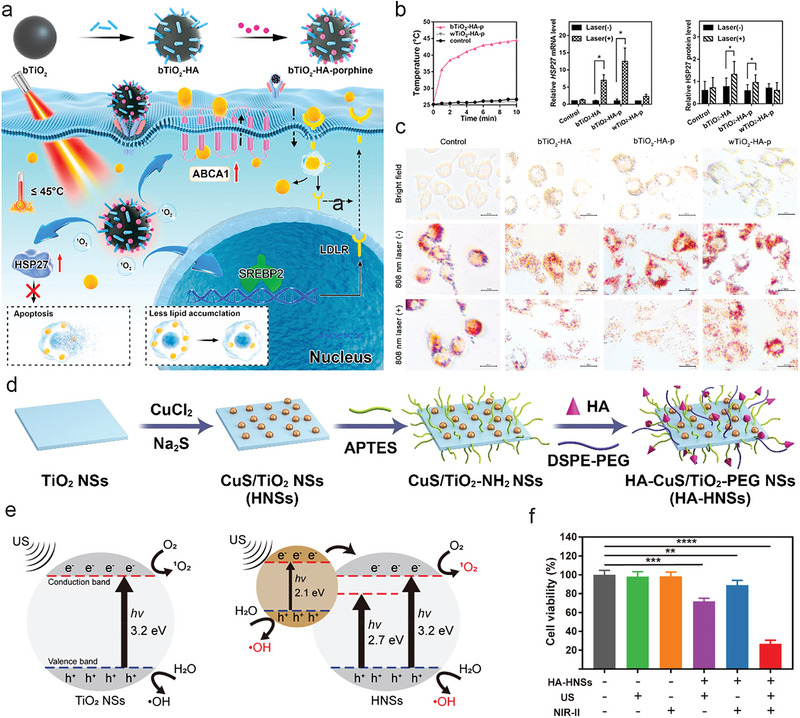
a) Schematic diagram of the synthesis process of bTiO_2_‐HA‐P and its therapy strategy. b) Temperature curves of bTiO_2_‐HA‐p and wTiO_2_‐HA‐p at 808 nm laser (left) and relative HSP27 mRNA level (middle) and protein level (right) after different treatments. c) Representative Oil Red O staining images after different treatments to assess the lipid burden of foam cells. *p < 0.05. a–c) Reproduced with permission.^[^ ^232^ ^]^ Copyright 2022, Elsevier. d) Schematic diagram of HA‐HNSs. e) The principle of HA‐HNSs‐mediated SDT. (f)Viability of macrophages under different treatments (*n* = 3). *, *P* < 0.05; **, *P* < 0.01; ***, *P* < 0.001; ****, *P* < 0.0001. d–f) Reproduced with permission.^[^ ^241^ ^]^ Copyright 2023, American Chemical Society.

Further combining PDT with PTT, Chlorin e6 (Ce6) was loaded with carbon nanocages and then electrostatic adsorbed dextran sulfate (DS) to target SR‐A on the surface of activated macrophages, which killed inflammatory macrophages and reduced inflammatory factor secretion via PTT/PDT (808 nm (1 W cm^−2^)/633 nm (80 mW cm^−2^), 5 min).^[^ ^112^ ^]^ However, high temperatures could cause damage to the tissue, hence mild phototherapy has been proposed and gained more recognition in various inflammatory disease models including atherosclerosis. This study used polydopamine nanoparticles as the core to generate photothermal effects with OPN antibody as the targeting component. After 10 min of irradiation with the 808 nm laser, mild PPT was produced to promote fibrosis within the lesion, stabilizing the plaque without causing damage to the blood vessels.^[^ ^231^ ^]^ Intriguingly, a black TiO_2_ modified with HA and porphyrin was constructed (denoted as bTiO_2_‐HA‐P) to perform mild PTT in combination with PDT (**Figure** **7**a). Porphyrin, a traditional photosensitizer, generated singlet oxygen (^1^O_2_) to promote foam cells apoptosis under NIR irradiation, and the black TiO_2_ nanoparticles with high photothermal conversion efficiency exerted PTT under 808 nm and 1 W cm^−2^ for 10 min. This mild PTT upregulated the expression of ABCA1 to promote cholesterol efflux, downregulated the LDLr expression to reduce lipid endocytosis, and eventually reduced foam cell formation, while raising the temperature to 44.5 °C as well as promoting the expression of HSP27 to maintain PDT‐mediated macrophage apoptosis within a stable range and reduce the adverse effects of excessive necrosis and apoptosis (Figure 7b,c).^[^ ^232^ ^]^

### 5.3 Sonodynamic Therapy (SDT)

SDT refers to the production of transient ROS from acoustic sensitizers with the exist of energy by cavitation effect under ultrasound excitation.^[^ ^233^ ^]^ SDT is similar to PDT, but its significant advantage is the large increase in penetration depth, reaching up to several centimeters in soft tissues.^[^ ^13b^ ^]^

Tian et al. proposed that 5‐aminolevulinic acid (ALA)‐mediated SDT (1.5 W cm^−2^) could alleviate plaque progression in the early year.^[^ ^234^ ^]^ Then they explored different intensities of ultrasound and the influence on atherosclerosis, the results showed that high‐intensity ultrasound‐mediated cell necrosis, while low‐intensity ultrasound‐induced cell apoptosis and cell burial of inflammatory cells, which was beneficial to alleviate the lesion. Furthermore, the investigators focused on the action mode of ALA‐mediated low‐intensity ultrasound in advanced APOE‐/‐ atherosclerotic mice. They found that SDT (0.5 W cm^−2^, 15 min) promoted cholesterol efflux via the PPARγ pathway and induced the apoptosis of inflammatory macrophages through the mitochondrial pathway. While upregulating the expression of Mertk to promote efferocytosis, which greatly diminished the inflammatory macrophages as well as the inflammatory burden within the plaque and rapidly stabilized the atherosclerosis.^[^ ^235^ ^]^ Meanwhile, sonosensitizers sinoporphyrin sodium (DVDMS)‐mediated SDT (mice: 0.8 W cm^−2^, rabbits: 1.5 W cm^−2^, both 30% duty factor, 15 min) was used to promote macrophage and endothelial cell apoptosis and inhibit neovascularization.^[^ ^236^ ^]^ Another report indicated that DVDMS‐mediated SDT (mice: 0.4 W cm^−2^, rabbits: 1.5 W cm^−2^, 15 min) upregulated PFN1 expression by activating the ROS‐Nrf2‐PFN1 signaling pathway and promoted transferrin efflux, which reduced iron retention in plaques and especially in the macrophages.^[^ ^237^ ^]^

Another representative sonosensitizer is a natural extract represented by hydroxysafflor yellow and curcumin. The former induced THP‐1 macrophage autophagy and cleared local inflammation in plaque by inhibiting PI3K/Akt/mTOR induction under US irradiation.^[^ ^238^ ^]^ The sonodynamic effect of curcumin mediated THP‐1 macrophage clearance by inducing mitochondrial damage.^[^ ^239^ ^]^ To further improve the solubility and efficacy of natural products, a curcumin nanosuspension‐mediated SDT (0.4 W cm^−2^, 1.0 MHz, 15 min) stabilized with PVPK30 and SDS was developed to enhance macrophage apoptosis under the mitochondrial injury pathway and interfere with macrophage polarization.^[^ ^240^ ^]^

In addition, inorganic TiO_2_ was selected to prevent early plaque progression due to its narrow bandgap (Figure 7d). This study combined SDT (0.5 W cm^−2^, 10 min) with PTT (0.8 W cm^−2^, 10 min) by preparing CuS/TiO_2_ heterostructured nanosheets (abbreviated as HA‐HNSs), which possessed a better electron‐hole separation effect (Figure 7e). The in vitro results showed that synergistic therapy synergistically induced macrophage apoptosis and inflammation clearance (Figure 7f).^[^ ^241^ ^]^

### 5.4 Immunotherapy

With the progressive understanding of atherosclerotic pathology, the current view holds that atherosclerosis has a complex immune inflammatory reaction, making immunotherapy possible.^[^ ^242^ ^]^ Early acute inflammatory cells are enriched in plaque, attracted major contributors (mononuclear macrophages), and eventually macrophages interacted with neutrophils and T cells to maintain inflammation. Immunotherapy can regulate the intraplaque inflammatory microenvironment by cutting off or alleviating the cascade response of the inflammatory network.^[^ ^243^ ^]^

**Figure 8:**
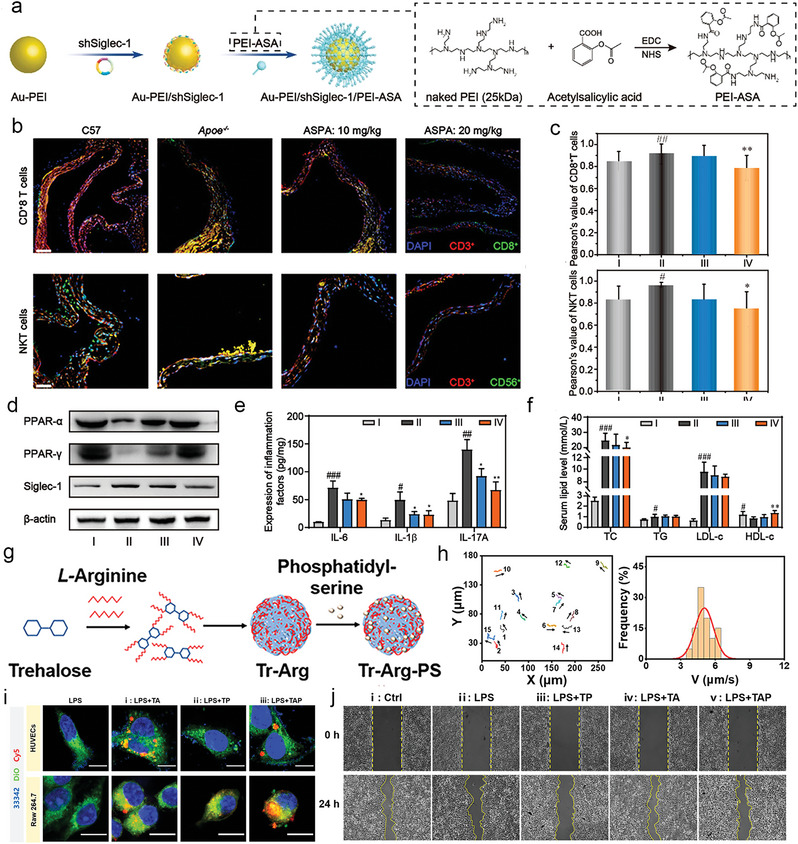
a) The diagrammatic sketch of ASPA and the covalent binding of PEI‐ASA. b) Typical immunofluorescence images and c) their quantitative analysis indicating CD8^+^T cells and NKT cells from the cardiac valve of healthy and atherosclerotic mice under different treatments. d) The results of PPAR‐α, PPAR‐γ, and Siglec‐1 protein expression. e) The expression of IL‐6, IL‐1β, and IL‐17A in extracted aortas after corresponding treatments. f) The levels of serum lipids. I: C57 group, II: Apoe‐/‐ group, III: ASPA: 10 mg per kg group, IV: ASPA: 20 mg per kg group. #*p* < 0.05, ##*p* < 0.01; *p < 0.05, **p < 0.01, ***p < 0.001. a–f) Reproduced with permission.^[^ ^244^ ^]^ Copyright 2022, American Chemical Society. g) Composition strategy of Tr‐Arg‐PS. h) Motion trajectories (left) and velocities (right) of TAP nanomotors. i) Confocal laser scanning microscope (CLSM) of cells uptake (red: sample, blue: nucleus, green: cell membrane). j) Representative microscope images of scratch wound healing assay under different treatments of HUVECs. g–j) Reproduced with permission.^[^ ^250^ ^]^ Copyright 2022, American Chemical Society.

Recently, a nano‐immunoplatform named ASPA was reported, which combined shSiglec‐1 with PEI‐Au NPs by electrostatic adsorption, and it was wrapped with pH‐responsive polyethylenimine‐acetylsalicylic acid (PEI‐ASA) to regulate the atherosclerotic inflammatory microenvironment (**Figure** **8**a). In order to verify the acid‐responsive hydrolysis of ASPA and the release of shSiglec and ASA, physiologically relevant buffers at different PH were used to simulate the in vivo environment, and the high‐performance liquid chromatography (HPLC) and ^1^H NMR spectrum were performed after co‐incubation. The results indicated that ASA can be released up to 90.2% at pH 5 buffer, but only 12.8% at pH 7.4 buffer. On the one hand, shSiglec‐1 silenced CD169 on macrophages, blocked the mutual recognition and interaction between macrophages and CD8^+^ T cells and NKT cells, and inhibited the lipids antigen presentation, which eventually reduced the infiltration of inflammatory cells (Figure 8b,c). On the other hand, the ASA up‐regulated peroxisome proliferator‐activated receptor α (PPAR‐α) and γ (PPAR‐γ) (Figure 8d). The former reduced the inflammatory Th17 cell differentiation and cooperatively inhibited the expression of inflammatory factors (Figure 8e). The latter participated in the cholesterol efflux pathway, decreased the formation of foam cells, and further impaired the lipid antigen presentation of macrophages (Figure 8f). Finally, ASPA successfully changed the atherosclerotic immune microenvironment and turned it from a complex thermal environment to a stable cold environment by remodeling various immune cells.^[^ ^244^ ^]^

Besides, rHDL is also a promising nano‐immunotherapeutic agent, which can specifically aim at myeloid cells. The researchers used phospholipid DMPC/MHPC and ApoA1 as ingredients to prepare r‐HDL on a large scale and loaded it with simvastatin via microfluidic homogenization. Radionuclide imaging demonstrated high enrichment of r‐HDL in the liver, kidney, spleen, and artery, while flow cytometry results manifested excellent myeloid targeting ability of r‐HDL. After the tail vein injection in ApoE‐/‐ mice on Western diet, the aorta was removed for analysis, and the results showed that immunotherapy rapidly attenuated excessive inflammatory cells within the lesion and reduced plaque burden.^[^ ^245^ ^]^

Collectively, although the reviewed immunotherapies have achieved significant efficacy, as for the immune system, the slightest nudge causes the widest chain reaction. Therefore, it requires a deep understanding of cell subsets, surface markers, and their role in different disease stages. Furthermore, immunotherapy is expected to achieve high precision in targeting therapies by modulating the size, surface charge, and ligand of nanoparticles. And now, the paradigm of nanomedicine has shifted from simply avoiding the immune system to actively engaging with it in a meaningful way.

### 5.5 Gas Therapy

Gas therapy is a new treatment modality based on special gas signal molecules that participate in various physiological regulation of cells and tissues.^[^ ^246^ ^]^ It has received wide attention in many fields due to its high efficiency and safety. Currently, the gases used in the treatment of atherosclerosis are nitric oxide (NO) and hydrogen (H_2_). NO, as a special endogenous substance, can regulate vasodilation, repair endothelial damage, reconstruct the endothelial barrier, and reduce oxidative stress, but its synthesis is impaired during atherosclerosis. To solve this problem, one of the methods is directly introducing exogenous NO into the lesion, and the liposomes, dendrimers, and macromolecular backbones are candidates as NO carriers. For example, Rink et al. assembled the NO transporter protein RSON and HDL together to construct bio‐nanoparticles that integrated NO delivery and lipid regulation, reducing the atherosclerotic burden and ischemia‐reperfusion injury.^[^ ^247^ ^]^ Similarly, to maintain favorable NO concentration, a dendrimer nanoplatform carrying 18 NO release units was constructed to finely modulate the slow release of NO.^[^ ^248^ ^]^ Another alternative is delivering NO donor L‐arginine into the atherosclerotic plaques to produce endogenous NO.^[^ ^249^ ^]^

Interestingly, the concept of “nanomotor” has been proposed recently that regarded NO production as a driving force to enhance the abilities of nanoparticles for the targeting, retention, and penetration into atherosclerotic plaques. In this research, carrier‐free nanomotor based on trehalose‐L arginine‐phosphatidylserine (Tr‐Arg‐PS) was structured to enable cascade targeting of intraplaque macrophages (Figure 8g). First, L‐arginine reacted with the high levels iNOS as well as ROS in the microenvironment of plaque and produced NO as driving force to achieve primary targeting, then relied on the affinity of PS to macrophages to realize secondary targeting. Motion is the key behavior of nanomotor. Brownian motion was only observed in normal RAW264.7 macrophages and HUVEC cells, while significant displacement of nanomotor for up to 24 h was observed in LPS‐induced inflammation cell models (Figure 8h). The CLSM results also exhibited significant enrichment of Tr‐Arg‐PS with motor structure in inflammatory cells (Figure 8i). After sequential treatments, the nanomotor repaired the integrity and consistency of the vascular endothelial barrier and presented the well‐aligned endothelium (Figure 8j).^[^ ^250^ ^]^

H_2_, an exogenous gas, has been reported to alleviate oxidative stress, reduce the expression of pro‐inflammatory cytokines, and promote cholesterol efflux from macrophages. However, it is difficult to directly deliver H_2_ in the blood circulation, so metallic palladium has been used as a hydrogen carrier as its interstitial void can accommodate hydrogen atoms. Palladium had been reported to come in a variety of shapes, and Xu et al. constructed a palladium nanopocket cube that can load up to 0.12% hydrogen atoms. The superior hydrogen storage and release activated the PPAR‐γ pathway, promoted lipid efflux and alleviated atherosclerosis.^[^ ^251^ ^]^ Attractively, a tetrapod needle‐like PdH nanoparticle was designed to perform living cell delivery depending on the chemotaxis towards macrophages within the plaque microenvironment. After entering the plaque, H_2_ was released from nanoparticle‐loaded live cells in several minutes under the 808 nm NIR laser irradiation. In addition, the hydrogen carrier palladium had nanoenzyme activity to remove ROS in the lesion and the spike‐like structure was able to induce macrophage autophagy, demonstrating significant therapeutic effects.^[^ ^252^ ^]^

### 5.6 Clinical Studies of Atherosclerosis Therapy in Nanomedicine

Although nanomaterials have demonstrated efficacy in animals with atherosclerosis, there are currently no formally approved nanomedicines, and only a few have entered clinical trials. Liposomes have received broad attention as excellent candidates. Previous reports on atherosclerosis clinical trials have included liposomal nanoparticle encapsulating prednisolone (LN‐PLP) and paclitaxel‐associated cholesterol‐rich non‐protein nanoemulsion (PTX‐LDE). Based on the safety and efficacy validation of LN‐PLP in rabbits, it has been approved for clinical trials.^[^ ^253^ ^]^ LN‐PLP exhibited a long half‐life of 45–63 h, which was 7–15 times that of free prednisolone. In addition, preoperative injection of LN‐PLP through the brachial vein of patients undergoing arterial stripping surgery showed targeting of macrophages but lacked anti‐inflammatory efficacy.^[^ ^254^ ^]^ Another study administered PTX‐LDE to severely atherosclerotic patients every three weeks for six consecutive weeks. This preliminary study suggested that using high‐dose PTX‐LDE (175 mg m^‐2^ body surface area) was safe. Four out of eight subjects in the experimental group had an average plaque volume reduction, but this was not statistically significant.^[^ ^255^ ^]^ Hence, longer‐term observations may be necessary.

In vivo, HDL participates in reverse cholesterol transport, so the use of rHDL mimetics may be feasible. The first rHDL (ETC‐216) composed of ApoAI Milano and palmitoyl‐oleoyl phosphatidyl choline (POPC) was associated with a significant reduction in plaque volume in acute coronary syndrome patients.^[^ ^256^ ^]^ However, clinical trials were suspended due to adverse reactions such as increased neutrophils and decreased lymphocytes. Subsequently, improvements were made to the formula, resulting in MDCO‐216. Results from a single‐ascending‐dose phase I study showed that the drug promoted ABCA1‐mediated cholesterol efflux in healthy individuals and stable coronary heart disease patients.^[^ ^257^ ^]^ However, in another randomized clinical trial focused on acute coronary syndrome patients, MDCO‐216 (*n* = 59) was compared with a placebo (*n* = 67) while all patients received statin therapy, and the addition of MDCO‐216 did not further reduce plaque volume.^[^ ^258^ ^]^ However, MDCO‐216 demonstrated good overall safety and tolerability, leaving room for further exploration.

CER‐001 is composed of ApoAI, dipalmitoylphosphatidylglycerol (DPPG), and sphingomyelin (SPM). Similarly, two clinical trials investigated the effect of CER‐001 on plaque regression in patients with acute coronary syndrome. The results showed that neither a low‐dose regimen (3 mg kg^‐1^) for up to 10 weeks nor a dose‐escalation approach reduced coronary atherosclerosis.^[^ ^259^ ^]^ CSL111 is formed by the combination of native ApoAI and phospholipids. In a randomized controlled trial, short‐term administration of CSL111 resulted in a statistically significant improvement in plaque characteristic index and coronary artery score, but there was still no statistically significant regression of plaque volume.^[^ ^260^ ^]^ CSL112 is another discoidal rHDL NPs similar to CSL111.^[^ ^261^ ^]^ The in vitro experiments have clearly demonstrated its ability to promote cholesterol efflux and reshape plaque characteristics. Single‐dose (NCT01129661) and multi‐dose escalating trials (NCT01281774) have completed phase I clinical trials, aiming to study the safety, tolerability, and pharmacokinetics of CSL112.^[^ ^257^ ^]^ The results of both two trials confirmed that CSL112 administration can increase ApoAI and pre‐β1‐HDL, but cannot seem to increase lipids promoting atherothrombotic risk, and no serious adverse events occurred.^[^ ^262^ ^]^ In another multicenter escalating dose trial (NCT02427035), there were no significant changes in liver and kidney function for all investigators, even in patients with moderate renal impairment.^[^ ^263^ ^]^ In addition, three phase II clinical trials have been completed. First, a single escalating dose trial of CSL112 (1.7 g, 3.4 g, and 6.8 g) was determined to be well tolerated in stable atherosclerotic patients.^[^ ^264^ ^]^ Subsequently, it was further determined that a dose of 6g of CSL112 was acceptable in patients with moderate renal impairment.^[^ ^265^ ^]^ And regardless of weight, gender, and race, a 6g dose of CSL112 is beneficial for acute myocardial infarction patients.^[^ ^262^, ^266^ ^]^ Encouraged by these findings, a phase III clinical trial (NCT03473223) is currently actively being conducted to study the efficacy and safety of CSL112 in patients with acute coronary syndrome.

Apart from the lipid‐based nanomaterials mentioned above, other types of nanomaterials have also been applied in clinical trials. A randomized, double‐blinded, placebo‐controlled clinical trial revealed that the natural compound curcumin micelle (80 mg once daily for 3 months) can prevent atherosclerosis by reducing high‐sensitivity C‐reactive protein in type II diabetic patients with mild to moderate coronary artery disease (CAD).^[^ ^267^ ^]^

Another team developed a silica‐gold core‐shell nanoparticle (NANO) for the treatment of atherosclerosis using plasmonic photothermal therapy (PPTT).^[^ ^268^ ^]^ The results showed that the treatment of NANO and PPTT indeed regressed total atheroma volume (TAV) and reduced mortality, complications, and thrombosis. However, the side effects cannot be ignored, such as an increase in the incidence of defects in erythrocyte membranes in patients treated with NANO.^[^ ^269^ ^]^ To improve long‐term safety and efficacy, the lesion preparation such as treated with stent first or treated with predilution by drug‐coated balloon prior to NANO treatment was found to be beneficial.^[^ ^270^ ^]^

Compared to bare metal stents, drug‐eluting stents (DES) are more effective in reducing restenosis and improving blood flow in patients receiving coronary stent implantation. COBRA PzF is a thin strut cobalt‐chromium alloy stent coated with Polyzene‐F. A single‐center clinical trial of 155 stents in 100 patients conducted a one‐year follow‐up and no acute adverse events were observed, providing an opportunity for further research on COBRA PzF.^[^ ^271^ ^]^ However, this was a small‐scale clinical trial with no control group. Subsequently, Maillard et al. conducted a multicenter prospective study (e‐Cobra) including 980 patients aiming to evaluate the safety and effectiveness of COBRA PzF in short‐term dual antiplatelet therapy (DAPT) patients. The patients were followed up for one year, and only 9% of them had major adverse cardiovascular events (MACE), and 0.7% of them experienced stent thrombosis.^[^ ^272^ ^]^ Furthermore, the team compared the COBRA PzF stents followed by DAPT for 14 days with FDA‐approved DES followed by guideline‐recommended (3‐6 months) DAPT therapy, and found that the former therapy reduced bleeding without promoting thrombosis.^[^ ^273^ ^]^ It should be noted that these results are from small‐scale and limited control group clinical trials, so more research and verification are needed before making any decisions.

## 6 Theranostic

Combining contrast agents and therapeutic agents in a single atherosclerosis theranostic nanoplatform can integrate diagnostic, therapeutic, and monitoring functions. It facilitates the diagnosis and treatment of diseases, as well as streamlines monitoring of disease progression, evaluation of therapeutic efficacy, and timely adjustment of drug administration strategies, thus enabling personalized treatments. The development of a multi‐functional theranostic nanoplatform for atherosclerosis is currently a hot research topic and remains challenging. In this section, we mainly discuss how nanomaterials have promoted the development of theranostic approaches and their current applications in atherosclerosis recently, with the aim of providing deep insight into the promotion of theranostic development (Table 2).

MR imaging‐based theranostic still relied on gadolinium and iron oxide, and based on existing reports, they were mostly constructed by loading diagnostic probes and therapeutic components into the same nanosystem to form theranostic probes.^[^ ^274^ ^]^ Interestingly, in one report, an iron oxide‐based mito‐magneto (MM) played the significant role of MR imaging, exhibiting favorable high contrast. The team directly selected biomimetic HDL nanoparticles with therapeutic effects to achieve dual‐targeted delivery of contrast agents to macrophages and mitochondria. This dual‐targeting mode achieved molecular‐level imaging of macrophages, reversed cholesterol transport (RCT), and oxidative stress relief targeted at mitochondria.^[^ ^275^ ^]^

FL imaging‐based theranostic have been found to be rapidly expanding in the field of cardiovascular disease, and substances that can produce fluorescence are diverse and easy to modify, making FL imaging an indispensable part of theranostic.^[^ ^276^ ^]^ I In addition to the previously mentioned fluorescence imaging substances, Ru(bpy)_3_Cl_2_, a FL molecular probe, was loaded into mesoporous silica systems along with the SIRT1 activator SRT1720 and targeted macrophages via coating CD36 antibody. Real‐time monitoring of inflammation cells in plaques was realized while reducing cholesterol content in macrophages by SRT1720.^[^ ^277^ ^]^

**Figure 9:**
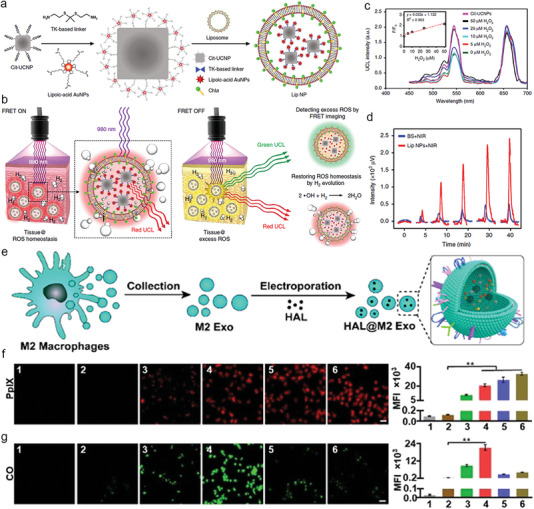
Schematic illustration of a) the composition of Lip NP b) and the principles of theranostic under light excitation of Lip NP. c) FL excitation spectra and linear correlation curve of Lip NP under different concentrations of H_2_O_2_ with a NIR laser (980 nm) for 30 min. d) H_2_ release after irradiation at different times. a–d) Reproduced with permission.^[^ ^278^ ^]^ Copyright 2020, Springer Nature. e) Composition strategy of HAL@M2 Exo. FL imaging (left) and quantitative analysis (right) of f) PpIX and g) CO under different treatments (1. Control 2. M2 Exo 3. HAL 4. HAL@M2 Exo 5. HAL+HO‐1 inhibitors 6. HAL@M2 Exo+HO‐1 inhibitors, *n* = 3). e–g) Reproduced with permission.^[^ ^279^ ^]^ Copyright 2020, Wiley‐VCH.

Besides, inspired by photosynthesis, a report proposed a chlorophyll a (Chla)‐modified liposome that can produce H_2_ under NIR light and continuously detect local ROS levels. The core of the liposome was coupled between lanthanide elements doped UCNPs and AuNPs via the thioketal‐based linker (**Figure** **9**a). On the one hand, the distance between UCNPs and AuNPs was short enough for Förster resonance energy transfer (FRET) in a normal environment. Therefore, the AuNPs (acceptor) were able to absorb the green upconversion luminescence (UCL) emitted by the UCNPs (donor) upon 980 nm excitation. When the liposome entered the plaque and monitored the excessive ROS in the lesion, the linker broken, and the distance between UCNPs and AuNPs increased. The green UCL (at 550 nm) emitted by the UCNPs cannot be absorbed by AuNPs, so it achieved in situ detection of ROS levels. On the other hand, when UCNPs were excited by 980 nm light, it produced red UCL (at 660 nm), which stimulated the photosensitizer Chla into the excited state and provided excitation electrons to AuNPs, while the citrates on the surface of UCNPs provided protons, and the protons are reduced to H_2_ by AuNPs, removing excess ROS (Figure 9b). The experimental results indicated that the higher the concentration of hydrogen peroxide (H_2_O_2_), the stronger the green fluorescence emitted, and there was a good linear relationship between them, without affecting the emission of red fluorescence (Figure 9c). At the same time, gas chromatography was used to measure the release of H_2_ under exposure to the infrared laser, and the H_2_ release amount gradually increased with the prolongation of irradiation time (Figure 9d). In vitro, after the irradiation with 980 nm near‐infrared laser, the ROS, pro‐inflammatory cytokine IL‐1β, and IL6 were significantly reduced. Consistent with the results of detection kits, FRET technology sensitively evaluated the level of H_2_O_2_ in cells. In summary, this report fulfilled the systematic combination of diagnosis and therapy by ingeniously applying the transfer of electrons and energy between donors and acceptors and the reduction catalysis of protons.^[^ ^278^ ^]^

Another study used exosomes from M2 macrophages to carry hexyl 5‐aminolevulinate hydrochloride (HAL@M2 Exo) by electroporation, fully utilizing the biosynthesis and metabolism of HAL for atherosclerotic diagnosis and treatment (Figure 9e). The metabolic intermediate of HAL was protoporphyrin IX (PPIX), a photosensitizer that emitted red fluorescence to track the lesion. Next, further biological metabolism occurred, which produced anti‐inflammatory and antioxidant substances carbon monoxide (CO) and bilirubin. In addition, M2 exosomes had excellent inflammatory chemotaxis activity for plaque targeting, and they carried anti‐inflammatory factors secreted by M2 macrophages to further clear local inflammation in the lesion. At the cellular level, FL imaging and quantitative analysis illustrated the red fluorescence emitted by ppIX and the green fluorescence of CO, which validated the feasibility of theranostic (Figure 9f,g).^[^ ^279^ ^]^

US is an ideal device for detecting vascular diseases and is typically used for the real‐time assessment of vascular wall lesions. Contrast‐enhanced ultrasound relies on the intravenous injection of microbubbles to artificially enhance the contrast of blood, thereby imaging normal or pathological structures of blood vessels. Inspired by this, microbubble‐mediated atherosclerotic theranostic has made significant progress recently.^[^ ^280^ ^]^ Since inflammatory macrophages within the plaque are an important part of atherosclerotic plaques, PLAG nanoparticles loaded with perfluorohexane (PFH) were designed to target macrophages for ultrasound imaging. At the same time, low‐intensity focused US (LIFU) was applied to the lesion to induce PFH to undergo a gas‐liquid phase transition. This process was accompanied by a series of physicochemical reactions known as the acoustic droplet vaporization (ADV) effect. In the ADV effect, bubbles regularly oscillated, expanded, contracted, or collapsed, potentially generated energy, destroyed cells, and promoted macrophage apoptosis, which achieved a similar effect to ablation, reversed plaque progression, and facilitated early diagnosis and intervention of vulnerable plaques.^[^ ^281^ ^]^ Similarly, perfluoropentane (PFP) can also be transformed into gas microbubbles through the ADV effect. A multifunctional nanoplatform with PFP as a core carrying sonosensitizer hematoporphyrin monomethyl ether (HMME) and MR/PA imaging probe MnFe_2_O_4_ was constructed to target neovascularization in atherosclerotic plaques. LIFU‐mediated endothelial cell apoptosis achieved complete inhibition of neovascularization, and ultimately, the plaque became stable.^[^ ^98^ ^]^

**Figure 10:**
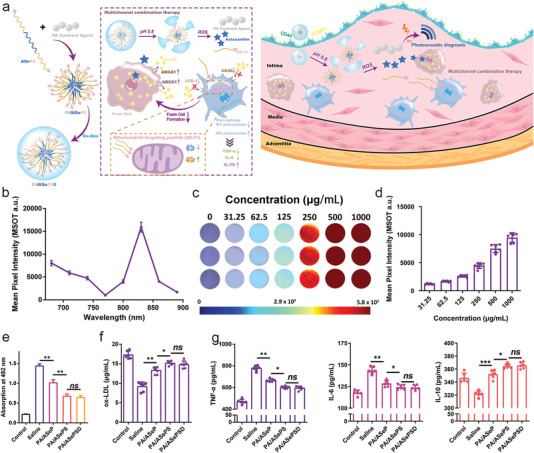
a) Schematic illustration of the composition structure and theranostic strategy of PMeTPP‐MBT. b) Photoacoustic absorption spectrum of PA/ASePSD. c) Photoacoustic images and d) quantitative analysis of PA/ASePSD at different concentrations (*n* = 3). e) Quantitative analysis of intracellular Oil red staining and f) the ox‐LDL in supernatant under different treatments 3D reconstruction PA images of the aorta and carotid arteries. g) The expression of various inflammatory factors (TNF‐α, IL‐6, and IL‐10) in macrophages (*n* = 6). *p < 0.05, **p < 0.01, ***p < 0.001; ns, no significance. Reproduced with permission.^[^ ^282^ ^]^ Copyright 2023, Wiley‐VCH.

PA imaging‐mediated theranostic made tremendous progress on latest research. A multifunctional and smart nanotheranostic agent controlled by pH and ROS dual switches with accurate cascade targeting ability for damaged endothelial cells, macrophages, and mitochondria has been proposed as a multi‐channel diagnostic and therapeutic system for atherosclerosis (**Figure** **10**a). This group innovatively synthesized a novel π‐conjugated polymer (PMeTPP‐MBT) for in vivo PA imaging. Based on it, they connected the therapeutic components SS‐31 peptide and astaxanthin to enable non‐invasive early theranostic of atherosclerosis (named as PA/ASePSD). The maximum photoacoustic absorption peak of PA/ASePSD exists at 830 nm, and there is a good linear relationship between the signal intensity and concentration (Figure 10b–d). Briefly, SS‐31 peptide was a versatile mitochondrial‐targeting peptide that restored mitochondrial function while reduced intracellular ROS and suppressed the expression of CD36 and LOX‐1 receptor on macrophage surfaces. Oil red staining and quantitative analysis of foam cells after treatment with PA/ASePSD revealed a significant decrease in intracellular lipid content. Consistent with the results of Oil red, the increase in ox‐LDL in the supernatant medium of the PA/ASePSD group confirmed the synergistic effect of inhibiting lipid endocytosis and enhancing lipid exocytosis (Figure 10e,f). Astaxanthin targeted foam cells and enhanced cholesterol efflux mediated by ABCA/G‐1, thereby reducing foaminess. The synergistic effects of both substances fulfilled lipid management and anti‐inflammatory to atherosclerosis (Figure 10g), and the introduction of PA imaging provided a new perspective for atherosclerotic theranostic.^[^ ^282^ ^]^

Vascular interventional surgery performs atherosclerotic diagnosis and treatment simultaneously in clinical practice, such as bare metal stents (BMS), drug‐eluting stents (DES), and drug‐coated balloons (DCB). However, BMS causes re‐narrowing,^[^ ^283^ ^]^ DES increases the risk of late thrombosis,^[^ ^284^ ^]^ and local retention and permeability of drugs in DCB are unsatisfactory.^[^ ^285^ ^]^ Nanomaterials for stent and balloon functionalization can enrich the types of interventional devices in vascular interventional surgery and improve the aforementioned issues. Inorganic nanomaterials such as TiO_2_ and magnesium fluoride (MgF_2_) nanofilm layers have been reported to promote endothelial cell growth and proliferation with confirmed biocompatibility in animals.^[^ ^286^ ^]^ Polymer nanomaterials are promising candidates for organic stent coatings due to their biodegradability and surface modifiability. Dopamine self‐polymerizes in alkaline environments to form a polydopamine (PDA) film on the stent surface, enhancing stent corrosion resistance, and promoting endothelial cell proliferation while reducing VSMC proliferation.^[^ ^287^ ^]^ The breakdown of PLGA ester linkages with oligomers and monomers is the main method for delivering drugs after hydrolysis.^[^ ^288^ ^]^ Interestingly, Huang et al. designed a NIR‐controlled tip‐separable microneedle‐loaded balloon with anti‐proliferation drugs paclitaxel encapsulated in the microneedle tip. A ring laser was placed in the catheter axis, emitting a NIR laser to heat and melt the phase‐change material lauric acid (LA) when the catheter was slowly removed from the blood vessel. Then gently withdrawing the balloon kept the drug delivery tip in the atherosclerotic lesion, slowly releasing the drug under the regulation of PLGA and polycaprolactone (PCL).^[^ ^289^ ^]^ Additionally, several new polymers such as chitosan (CS),^[^ ^290^ ^]^ polycyclodextrin,^[^ ^291^ ^]^ polyurethane,^[^ ^292^ ^]^ and phosphorylcholine^[^ ^293^ ^]^ are also involved in the application of nano‐coatings. Carbon‐based nanomaterials such as carbon nanotubes and graphene oxide also have some reports due to their ability to promote cholesterol efflux and photoresponse performance.^[^ ^294^ ^]^ Inspired by mussels, biomimetic nanomaterial coating has carried out some research, in which mussel adhesive protein is extracted to replace polymer coating and enhance the adhesion of the coating to the stent.^[^ ^295^ ^]^ Further modification of groups on mussel adhesive proteins can connect biological effect molecules and exert anti‐atherosclerosis applications.^[^ ^296^ ^]^ Encouraged by the above results, it is believed that nanomaterial coating stents and balloons provide an alternative theranostic strategy for patients.

In addition to the above, theranostic for atherosclerosis based on CT or nuclear medicine had also been reported.^[^ ^297^ ^]^ For example, AuNPs have a high atomic number and are suitable for CT imaging. Besides, they have a broad and distinct absorption peak in the NIR and a high photothermal conversion efficiency, which are capable of photothermal ablation.^[^ ^298^ ^]^ This multi‐functional theranostic agent can utilize the inherent diagnostic and therapeutic properties of a single nanomaterial, greatly enhancing the utilization and providing innovative ideas for further therapy of inflammatory diseases.

## 7 Biosafety

The unique properties and application potential of nanomaterials have made nanomedicine a research hotspot in fields like disease diagnosis, drug delivery, molecular technology, etc. Although many engineered nanomaterials have shown enormous potential, most of the research for atherosclerosis is in its early stages, and there are some issues need to be resolved. Unlike conventional medical drugs, the special physicochemical properties of nanomaterials may have unpredictable behaviors and impacts on living organisms. Therefore, the issue of biosafety should be an important part of nanomedicine research for final clinical translation.^[^ ^299^ ^]^

The biosafety assessment of nanomaterials in atherosclerosis involves multiple fields, such as nanotechnology, fluid dynamics, pathology, and toxicology, and it is a complex evaluation process. A comprehensive evaluation method is necessarily required, which typically includes the followings:

- In vivo distribution and metabolism assessment of nanomaterials:^[^ ^300^ ^]^ The main factors that affect distribution and metabolism are the characteristics of nanomaterials, such as targeting substances, size, potential, shape, stiffness, and route of administration.^[^ ^301^ ^]^ The evaluation typically requires the use of biological imaging techniques and other biological detection methods. It is worth noting that when nanomaterials enter the body, they may adsorb proteins and form a “protein corona,” which slows down the clearance from the body or mediates special drug delivery methods.^[^ ^302^ ^]^ A literature validated the critical role of protein corona in atherosclerotic mice. Designed anionic liposomes interacted with proteins in the serum and formed a protein corona, in which the complement component C1q significantly mediated the cellular uptake of liposomes.^[^ ^121b^ ^]^ Subsequently, nanomaterials mostly enter tissues or organs through the bloodstream and are mainly metabolized and excreted by the liver, kidneys, and lungs.^[^ ^303^ ^]^ Wang and co‐workers designed spherical MSNs with a diameter of 61.4 nm and found drug accumulation in the liver and gallbladder at 0.5 and 1 h after intravenous injection respectively, and gradually disappeared after 24 h, suggesting that the metabolic pathway may be the hepatobiliary system.^[^ ^277^ ^]^ In addition, disk‐shaped rHDL NPs with a diameter of 23.6 nm were excreted through the hepatobiliary and urinary systems of atherosclerotic mice.^[^ ^245^ ^]^ It is consistent with previous views that small‐sized nanomaterials (below 10 nm) are mainly metabolized by the kidneys after glomerular filtration, large nanomaterials (above 50 nm) are mainly metabolized by the liver, and medium‐sized nanomaterials (10–50 nm) often exhibit the joint metabolic pattern.
- Biological evaluation: It is supposed to evaluate the effect of nanomaterials on body weight, behavior, and mental state as well as biological molecules such as DNA, RNA, and proteins. The biological evaluation of nanomaterials needs to comprehensively consider the different stages of cellular interactions and tissue transportation, and the content involved is relatively extensive.^[^ ^304^ ^]^ For the diagnosis and/or therapy of atherosclerosis, intravenous injection is the most commonly used method. Nanomaterials often directly interact with blood components therefore it is particularly crucial to analyze the types, morphology, proportion, and number of complete blood cells. In addition, Wang et al. designed a biomimetic red blood cell membrane‐coated PLGA nanomaterial for a one‐month treatment, and regularly monitored the body weight of ApoE‐/‐ mice during the treatment period. After the treatment, further evaluation of the function index of vital organs such as the heart, liver, spleen, lungs, and kidneys was performed to obtain a more comprehensive biological assessment. This study demonstrated the biosafety advantages of biomimetic nanomaterials as a next‐generation drug delivery system for atherosclerotic management.^[^ ^127^ ^]^
- Immunological evaluation: Nanomaterials that enter the body may have close contact with the immune system and cause direct or indirect effects.^[^ ^305^ ^]^ Immunological evaluation of nanomaterials needs to be started from both in vitro and in vivo aspects. In vitro evaluation includes co‐culturing the nanomaterials with immune cells to observe their morphology, proliferation, differentiation, and changes in cytokines. The in vivo evaluation mainly involves immune activation evaluation and immune toxicity evaluation. For example, Lameijer and co‐workers selected RAW264.7 cells and bone marrow‐derived macrophages to assess the immunological safety of rHDL NPs. Nanomaterials were cocultured with these macrophages and tested immune cell function. The results indicated that these rHDL NPs did not affect the expression of chemokine ligand 2 in macrophages, nor did it affect the immune response of macrophages to lipopolysaccharide. Further, the expression of cytokine chemokines such as IL‐6, chemokine ligand 2, and TNF‐𝛼 were detected within the normal range after the rHDL NPs were injected into atherosclerotic mice, and no systemic inflammatory response and potential adverse immune effects were observed. These results demonstrated the great potential of rHDL NPs as a new‐generation nanoplatform for the treatment of atherosclerosis.^[^ ^243^ ^]^
- Toxicology evaluation: The toxicology evaluation of nanomaterials needs to evaluate toxic effects on cells, various organs, and biological systems, which mainly embraces acute and chronic toxicity evaluation.^[^ ^306^ ^]^ Cell culture, biological slice technology, and blood biochemical detection methods can assist toxicological evaluation to ensure the safe application of nanomaterials. It has been demonstrated that several types of nanomaterials for atherosclerosis exhibited low toxicity both in vitro and in vivo. On the one hand, cell proliferation, and death assessment is the most common method in vitro, and in one report, a kind of manganese ferrite (MnFe_2_O_4_)‐based nanomaterial at the concentration of 31.25 µg mL^‐1^ was safe for the growth of RAEC cells.^[^ ^98^ ^]^ Another metal element Zr‐contained MOFs did not lead to cytotoxic death at the concentration of 32 µg mL^‐1^ after coincubation with cells for 24 h.^[^ ^221^ ^]^ Comparatively, organic nanomaterials without metal elements demonstrated superior results. For instance, a rapamycin‐loaded polymer exhibited a safe concentration of up to 300 µg mL^‐1^ at the cellular level.^[^ ^124^ ^]^ On the other hand, the in vivo toxicity assessments require the observation of blood samples and major organs (e.g., heart, liver, spleen, lungs, and kidneys). The above MnFe_2_O_4_, MOFs, and polymer all exhibited outstanding biosafety and the blood indices and tissue sections had no significant differences between the treatment and control group. In addition, another study on palladium‐hydrogen nanopocket cubes also analyzed serum triglycerides and HDL in atherosclerotic mice with no significant abnormalities, suggesting that nanomaterials are suitable for biological application.^[^ ^251^ ^]^

It is worth noting that there are remarkable individual differences between nanomaterials, and they need to be treated differently during biological safety evaluation. Although breakthroughs have been made in the application of nanomaterials, we should not be complacent and need to consider the challenges they face in terms of biosafety and biocompatibility.^[^ ^307^ ^]^

## 8 Summary and Prospect

**Figure 11:**
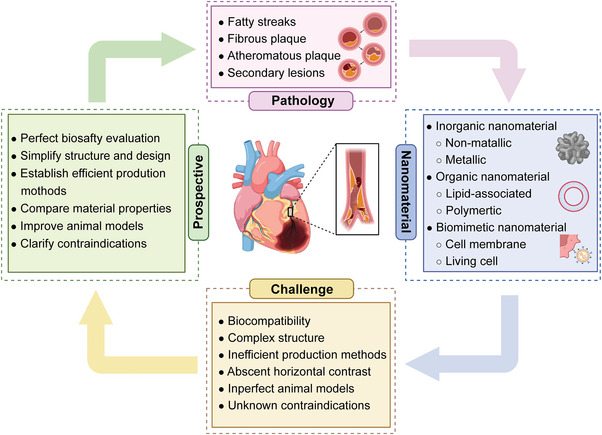
Schematic illustration of nanomedicine application (including pathological progress, the classification of nanomaterials, facing challenges and prospects) in atherosclerosis. Figure was created with BioRender.com.

In this review, we elaborate on the pathological basis of atherosclerosis, the characteristics of nanomaterials, the applications of nanomedicine in atherosclerosis, and the assessment of biosafety, in order to provide useful references for further research in this field. The application of nanomedicine in atherosclerosis involves many pathological processes, such as inflammatory burden, immune cell infiltration, lipid accumulation, endothelial injury, glycolysis, neovascularization, and thrombosis. The commonly used nanoplatforms include liposome, micelle, polymer, metallic oxide, AuNP, carbon‐based nanomaterial, QD, membrane‐coated biomimetic nanoparticle, living cell, cell‐derived extracellular vesicles, etc. These nanomaterials passively target plaques through the EPR effect, or specifically target certain components within plaques through modified proteins, peptides, and other ligands. On the one hand, the use of nanomaterials has expanded the imaging range from basic anatomical imaging to molecular functional imaging, including MR, CT, FL, radioisotopes, PA, OCT, and multimodal imaging, enabling early detection of plaques, distinguishing the stable and vulnerable plaques, and providing more optimized diagnostic data for the clinic. On the other hand, the methods of atherosclerotic therapy can be roughly categorized into five types, including drug delivery, phototherapy, SDT, immunotherapy, and gas therapy. It has demonstrated the remarkable abilities of these nanomaterials to inhibit plaque formation. Furthermore, the biosafety evaluation including in vivo distribution and metabolism assessment, biological evaluation, immunological evaluation, and toxicology evaluation of various nanomedicine for biomedical applications were thoroughly discussed (**Figure** **11**).

In the past decade, the scientific community has witnessed significant breakthroughs in the biomedical applications of nanomaterials. In the field of nanomedicine, cardiovascular diseases have become the second‐largest application branch after cancer. The term “atherosclerosis” is also appearing more frequently in the field of nanomedicine. Even though the application of nanomedicine is still in the research stage, it has demonstrated fascinating prospects based on the improvement of diagnostic capabilities, therapeutic effects, and even theranostic assessment. However, in order to achieve clinical translation and long‐run development, there is still an urgent need to consider and address several crucial challenges and issues:

- The unknown interaction between nanomaterials and biological systems is a major obstacle to the application of nanomaterials in biomedical fields. Especially for atherosclerosis, a chronic cardiovascular disease, lesions spread over various parts of the body including the heart, brain, kidneys, and limbs. Before targeting the atherosclerotic lesions, nanomaterials generally encounter many biological components in the body and manifest a wide distribution range whether injected intravenously or orally. Therefore, scientists ought to conduct more detailed biocompatibility and biosafety assessments, including risk‐benefit assessments for blood circulation, distribution, uptake, toxicity, immune activation, biodegradation, metabolism, and elimination. It is a critical prerequisite for achieving further clinical translation.
- Material design for atherosclerosis is cumbersome. An increasing number of reports tend to prepare multifunctional nanomaterials involving various chemical components, which may inadvertently increase the complexity of the nanoparticles. In the field of atherosclerotic theranostic, the complex components also bring unprecedented challenges to the biocompatibility assessment. For example, researchers cannot accurately evaluate the biological effects of each component under the premise of control variables, let alone speculate on the subtle results of interactions between various components. Thus, material design for atherosclerotic diagnosis and/or therapy should not be a combination and stacking of complex components but should fully explore the deeper and multifaceted effects of a certain material, subtract components from the material and make each component have a supportive and constructive relationship with each other.
- Nanomaterials need to be improved in production methods. Atherosclerosis is a highly prevalent chronic disease that requires long‐term administration. However, most existing nanomaterials are prepared through manual synthesis in the laboratory and have low yields. These hand‐prepared nanomaterials are often differences between batches including material size, yield, drug loading, and other parameters. Therefore, high‐precision and large‐scale commercial production methods need to be established to enhance the reproducibility of material parameters and performance, as well as meet clinical translation and application needs.
- The nanomaterial system lacks horizontal comparison. The number of articles on treating atherosclerosis is increasing exponentially, and the nanomaterial system is diverse with most reports claiming significant efficacy. However, there has been no scientific and systematic evaluation of which nanomaterial has the optimal effect. According to existing reports, the pathological composition within atherosclerosis is uneven and unequal, which may be dominated by lipids, inflammatory cells, neovascularization, or matrix proteins. Therefore, the same nanomaterial may exhibit significantly different effects in different individuals. It is necessary to compare the functions of nanomaterials based on the pathological types of atherosclerosis, which may be helpful for individualized diagnosis and treatment of patients.
- The animal models suitable for studying atherosclerosis need to be expanded. Most animal models used in atherosclerotic research related to nanomedicine are mice and rabbits. Although models used to simulate human atherosclerosis (such as ApoE‐/‐ mice, LDLr‐/‐mice, balloon‐injured rabbits) contribute to understanding the pathophysiology of atherosclerosis and validating the emerging therapies to some extent, their pathologic condition differs from that of the human body. Moreover, large animals such as pigs and monkeys are costly. Therefore, establishing a mature atherosclerotic model that is similar to humans is an urgent problem that needs to be solved.
- The contraindications of nanomedicine for the use of atherosclerosis are still unknown. Generally, patients with atherosclerosis are often accompanied by other complications including coronary heart disease, stroke, diabetes, obesity, liver and kidney dysfunction, etc. It is difficult to study the feasibility and/or contraindications of applying nanoparticles in complex disease populations. Therefore, it may be possible to identify contraindications of nanomedicine by constructing an animal model with multiple diseases.

These challenges exactly pose resistance to the development of nanomedicine to some degree. However, they also drive us to think further about the direction of our future efforts and guide us from multiple dimensions to promote the development and clinical translation of the next wave of nanomedicine in atherosclerosis theranostics. In addition to solving the above obstacles, we must deepen our understanding of atherosclerosis to make great strides forward through more extensive research. In the future, as the key scientific issues and technical challenges related to nanomedicine are resolved, and the preparation methods and evaluation measures based on multidisciplinary fields are standardized, it is highly expected that the rapid development of nanomedicine can bring sustained innovation to the diagnosis and treatment of atherosclerosis, breaking further development constraints. It is believed that in the near future, we can definitely achieve significant progress and more exciting breakthroughs in the field of atherosclerosis and even the entire medical field through nanomedicine.

## Conflict of Interest

The authors declare no conflict of interest.

## References

[1] a) M. Zhou , H. Wang , X. Zeng , P. Yin , J. Zhu , W. Chen , X. Li , L. Wang , L. Wang , Y. Liu , J. Liu , M. Zhang , J. Qi , S. Yu , A. Afshin , E. Gakidou , S. Glenn , V. S. Krish , M. K. Miller‐Petrie , W. C. Mountjoy‐Venning , E. C. Mullany , S. B. Redford , H. Liu , M. Naghavi , S. I. Hay , L. Wang , C. J. L. Murray , X. Liang , Lancet 2019, 394, 1145; 31248666 10.1016/S0140-6736(19)30427-1 PMC6891889

[2] P. Libby , J. E. Buring , L. Badimon , G. K. Hansson , J. Deanfield , M. S. Bittencourt , L. Tokgözoğlu , E. F. Lewis , Nat. Rev. Dis. Primers 2019, 5, 56. 31420554 10.1038/s41572-019-0106-z

[3] a) M. R. Maurya , S. Gupta , J. Y.‐S. Li , N. E. Ajami , Z. B. Chen , J. Y. J. Shyy , S. Chien , S. Subramaniam , Proc. Natl. Acad. Sci. USA 2021, 118, e2023236118; 33468662 10.1073/pnas.2023236118 PMC7848718

[4] W. Herrington , B. Lacey , P. Sherliker , J. Armitage , S. Lewington , Circ. Res. 2016, 118, 535. 26892956 10.1161/CIRCRESAHA.115.307611

[5] a) N. S. Parikh , R. F. Gottesman , J. Am. Coll. Cardiol. 2021, 77, 899; 33602473 10.1016/j.jacc.2020.12.046

[6] a) K. M. Sturgeon , L. Deng , S. M. Bluethmann , S. Zhou , D. M. Trifiletti , C. Jiang , S. P. Kelly , N. G. Zaorsky , Eur. Heart J. 2019, 40, 3889; 31761945 10.1093/eurheartj/ehz766 PMC6925383

[7] N. V. Mushenkova , V. I. Summerhill , D. Zhang , E. B. Romanenko , A. V. Grechko , A. N. Orekhov , Int. J. Mol. Sci. 2020, 21, 2992. 32340284 10.3390/ijms21082992 PMC7216001

[8] V. Aboyans , J.‐B. Ricco , M.‐L. E. L. Bartelink , M. Bjorck , M. Brodmann , T. Cohnert , J.‐P. Collet , M. Czerny , M. De Carlo , S. Debusa , C. Espinola‐Klein , T. Kahan , S. Kownator , L. Mazzolai , A. R. Naylora , M. Roffi , J. Rotherb , M. Sprynger , M. Tendera , G. Tepe , M. Venermoa , C. Vlachopoulos , I. Desormais , Rev. Esp. Cardiol. 2018, 71, 111.e1. 29425606 10.1016/j.rec.2017.12.014

[9] M. Lee , C.‐Y. Cheng , Y.‐L. Wu , J.‐D. Lee , C.‐Y. Hsu , B. Ovbiagele , JAMA Neurol. 2022, 79, 349. 35188949 10.1001/jamaneurol.2021.5578 PMC8861901

[10] C. B. Newman , D. Preiss , J. A. Tobert , T. A. Jacobson , R. L. Page , L. B. Goldstein , C. Chin , L. R. Tannock , M. Miller , G. Raghuveer , P. B. Duell , E. A. Brinton , A. Pollak , L. T. Braun , F. K. Welty , Arterioscler., Thromb., Vasc. Biol. 2019, 39, e38. 30580575 10.1161/ATV.0000000000000073

[11] J. S. Lawton , J. E. Tamis‐Holland , S. Bangalore , E. R. Bates , T. M. Beckie , J. M. Bischoff , J. A. Bittl , M. G. Cohen , J. M. DiMaio , C. W. Don , S. E. Fremes , M. F. Gaudino , Z. D. Goldberger , M. C. Grant , J. B. Jaswal , P. A. Kurlansky , R. Mehran , T. S. Metkus , L. C. Nnacheta , S. V. Rao , F. W. Sellke , G. Sharma , C. M. Yong , B. A. Zwischenberger , Circulation 2022, 145, e4. 34882436 10.1161/CIR.0000000000001039

[12] M. Venermo , M. Sprynger , I. Desormais , M. Björck , M. Brodmann , T. Cohnert , M. De Carlo , C. Espinola‐Klein , S. Kownator , L. Mazzolai , R. Naylor , C. Vlachopoulos , J.‐B. Ricco , V. Aboyans , Eur. J. Vasc. Endovasc. Surg. 2019, 58, 641. 31685166 10.1016/j.ejvs.2019.06.017

[13] a) Y. Zhang , M. Li , X. Gao , Y. Chen , T. Liu , J. Hematol. Oncol. 2019, 12, 137; 31847897 10.1186/s13045-019-0833-3 PMC6918551

[14] a) R. M. Williams , S. Chen , R. E. Langenbacher , T. V. Galassi , J. D. Harvey , P. V. Jena , J. Budhathoki‐Uprety , M. Luo , D. A. Heller , Nat. Chem. Biol. 2021, 17, 129; 33414556 10.1038/s41589-020-00690-6 PMC8288144

[15] a) S. N. Bhatia , X. Chen , M. A. Dobrovolskaia , T. Lammers , Nat. Rev. Cancer 2022, 22, 550; 35941223 10.1038/s41568-022-00496-9 PMC9358926

[16] Y. Liu , Y. Cheng , C. Zhao , H. Wang , Y. Zhao , Adv. Sci. 2022, 9, e2104272. 10.1002/advs.202104272 PMC8811803 34816629

[17] L. Yang , Z. Zhou , J. Song , X. Chen , Chem. Soc. Rev. 2019, 48, 5140. 31464313 10.1039/c9cs00011a PMC6768714

[18] D. Furtado , M. Björnmalm , S. Ayton , A. I. Bush , K. Kempe , F. Caruso , Adv. Mater. 2018, 30, 1801362. 10.1002/adma.201801362 30066406

[19] Y. Shi , R. van der Meel , X. Chen , T. Lammers , Theranostics 2020, 10, 7921. 32685029 10.7150/thno.49577 PMC7359085

[20] M. P. Vincent , J. O. Navidzadeh , S. Bobbala , E. A. Scott , Cancer Cell 2022, 40, 255. 35148814 10.1016/j.ccell.2022.01.006 PMC8930620

[21] a) X. Liang , H. Li , X. Li , X. Tian , A. Zhang , Q. Luo , J. Duan , Y. Chen , L. Pang , C. Li , X.‐J. Liang , Y. Zeng , J. Yang , Acta Pharm. Sin. B 2022, 36, 841; 10.1016/j.apsb.2022.04.002 PMC9939301 36815039

[22] a) T. H. Nøst , K. Alcala , I. Urbarova , K. S. Byrne , F. Guida , T. M. Sandanger , M. Johansson , Eur. J. Epidemiol. 2021, 36, 841; 34036468 10.1007/s10654-021-00752-6 PMC8416852

[23] a) J. He , W. Zhang , X. Zhou , F. Xu , J. Zou , Q. Zhang , Y. Zhao , H. He , H. Yang , J. Liu , Bioact. Mater. 2023, 19, 115; 35475030 10.1016/j.bioactmat.2022.03.041 PMC9010555

[24] a) M. Punjabi , L. Xu , A. Ochoa‐Espinosa , A. Kosareva , T. Wolff , A. Murtaja , A. Broisat , N. Devoogdt , B. A. Kaufmann , Arterioscler., Thromb. Vasc. Biol. 2019, 39, 2520; 31597443 10.1161/ATVBAHA.119.313088

[25] a) N. Distasio , F. Dierick , T. Ebrahimian , M. Tabrizian , S. Lehoux , Acta Biomater. 2022, 143, 356; 35257950 10.1016/j.actbio.2022.02.043

[26] a) Y. Dai , X. Sha , X. Song , X. Zhang , M. Xing , S. Liu , K. Xu , J. Li , Int. J. Nanomed. 2022, 17, 5413; 10.2147/IJN.S371873 PMC9677925 36419720

[27] M. A. Gimbrone , Ann. N. Y. Acad. Sci. 1987, 516, 5. 3439744 10.1111/j.1749-6632.1987.tb33025.x

[28] S. González‐Ramos , M. Paz‐García , C. Rius , A. Del Monte‐Monge , C. Rodríguez , V. Fernández‐García , V. Andrés , J. Martínez‐González , M. A. Lasunción , P. Martín‐Sanz , O. Soehnlein , L. Boscá , FASEB J. 2019, 33, 3912. 30496704 10.1096/fj.201801231RR

[29] M. A. Gimbrone , G. García‐Cardeña , Circ. Res. 2016, 118, 620. 26892962 10.1161/CIRCRESAHA.115.306301 PMC4762052

[30] B. A. Ference , J. J. P. Kastelein , A. L. Catapano , JAMA, J. Am. Med. Assoc. 2020, 324, 595. 10.1001/jama.2020.5685 32717042

[31] I. Tabas , K. J. Williams , J. Borén , Circulation 2007, 116, 1832. 17938300 10.1161/CIRCULATIONAHA.106.676890

[32] B. A. Ference , H. N. Ginsberg , I. Graham , K. K. Ray , C. J. Packard , E. Bruckert , R. A. Hegele , R. M. Krauss , F. J. Raal , H. Schunkert , G. F. Watts , J. Borén , S. Fazio , J. D. Horton , L. Masana , S. J. Nicholls , B. G. Nordestgaard , B. van de Sluis , M.‐R. Taskinen , L. Tokgözoglu , U. Landmesser , U. Laufs , O. Wiklund , J. K. Stock , M. J. Chapman , A. L. Catapano , Eur. Heart J. 2017, 38, 2459. 28444290 10.1093/eurheartj/ehx144 PMC5837225

[33] S. Gao , D. Zhao , M. Wang , F. Zhao , X. Han , Y. Qi , J. Liu , Can. J. Cardiol. 2017, 33, 1624. 29173602 10.1016/j.cjca.2017.07.015

[34] S. Yui , T. Sasaki , A. Miyazaki , S. Horiuchi , M. Yamazaki , Arterioscler. Thromb. 1993, 13, 331. 8443137 10.1161/01.atv.13.3.331

[35] K. Kugiyama , S. A. Kerns , J. D. Morrisett , R. Roberts , P. D. Henry , Nature 1990, 344, 160. 2106627 10.1038/344160a0

[36] a) Y. M. Park , Exp. Mol. Med. 2014, 46, e99; 24903227 10.1038/emm.2014.38 PMC4081553

[37] A. Akhmedov , I. Rozenberg , F. Paneni , G. G. Camici , Y. Shi , C. Doerries , A. Sledzinska , P. Mocharla , A. Breitenstein , C. Lohmann , S. Stein , T. von Lukowicz , M. O. Kurrer , J. Borén , B. Becher , F. C. Tanner , U. Landmesser , C. M. Matter , T. F. Lüscher , Eur. Heart J. 2014, 35, 2839. 10.1093/eurheartj/eht532 24419805

[38] a) M. S. Brown , J. L. Goldstein , Nature 1990, 343, 508; 2300201 10.1038/343508a0

[39] B. W. Parks , A. J. Lusis , N. Engl. J. Med. 2013, 369, 2352. 24328470 10.1056/NEJMcibr1312709 PMC3934498

[40] C. S. Robbins , I. Hilgendorf , G. F. Weber , I. Theurl , Y. Iwamoto , J. L. Figueiredo , R. Gorbatov , G. K. Sukhova , L. M. Gerhardt , D. Smyth , C. C. Zavitz , E. A. Shikatani , M. Parsons , N. van Rooijen , H. Y. Lin , M. Husain , P. Libby , M. Nahrendorf , R. Weissleder , F. K. Swirski , Nat. Med. 2013, 19, 1166. 23933982 10.1038/nm.3258 PMC3769444

[41] S. Frambach , R. de Haas , J. A. M. Smeitink , G. A. Rongen , F. G. M. Russel , T. J. J. Schirris , Pharmacol. Rev. 2020, 72, 152. 31831519 10.1124/pr.119.017897

[42] I. F. Charo , M. B. Taubman , Circ. Res. 2004, 95, 858. 15514167 10.1161/01.RES.0000146672.10582.17

[43] a) Y. Kojima , I. L. Weissman , N. J. Leeper , Circulation 2017, 135, 476; 28137963 10.1161/CIRCULATIONAHA.116.025684 PMC5302553

[44] A. Tajbakhsh , M. Rezaee , P. T. Kovanen , A. Sahebkar , Pharmacol. Ther. 2018, 188, 12. 29444453 10.1016/j.pharmthera.2018.02.003

[45] D. A. Chistiakov , A. V. Grechko , V. A. Myasoedova , A. A. Melnichenko , A. N. Orekhov , J. Cell. Mol. Med. 2018, 22, 1366. 29364567 10.1111/jcmm.13462 PMC5824421

[46] Y. Döring , M. Drechsler , S. Wantha , K. Kemmerich , D. Lievens , S. Vijayan , R. L. Gallo , C. Weber , O. Soehnlein , Circ. Res. 2012, 110, 1052. 22394519 10.1161/CIRCRESAHA.112.265868

[47] X. Zhou , A. Nicoletti , R. Elhage , G. K. Hansson , Circulation 2000, 102, 2919. 11113040 10.1161/01.cir.102.24.2919

[48] J. Li , K. Ley , Arterioscler., Thromb., Vasc. Biol. 2015, 35, 40. 25301842 10.1161/ATVBAHA.114.303227 PMC4429868

[49] M. A. Houtkamp , O. J. de Boer , C. M. van der Loos , A. C. van der Wal , A. E. Becker , J. Pathol. 2001, 193, 263. 11180175 10.1002/1096-9896(2000)9999:9999<::AID-PATH774>3.0.CO;2-N

[50] J. Wang , A. K. Uryga , J. Reinhold , N. Figg , L. Baker , A. Finigan , K. Gray , S. Kumar , M. Clarke , M. Bennett , Circulation 2015, 132, 1909. 26416809 10.1161/CIRCULATIONAHA.115.016457

[51] P. Lacolley , V. Regnault , A. Nicoletti , Z. Li , J. B. Michel , Cardiovasc. Res. 2012, 95, 194. 22467316 10.1093/cvr/cvs135

[52] J. J. Boyle , P. L. Weissberg , M. R. Bennett , Arterioscler., Thromb., Vasc. Biol. 2002, 22, 1624. 12377740 10.1161/01.atv.0000033517.48444.1a

[53] M. C. H. Clarke , T. D. Littlewood , N. Figg , J. J. Maguire , A. P. Davenport , M. Goddard , M. R. Bennett , Circ. Res. 2008, 102, 1529. 18497329 10.1161/CIRCRESAHA.108.175976

[54] G. L. Basatemur , H. F. Jørgensen , M. C. H. Clarke , M. R. Bennett , Z. Mallat , Nat. Rev. Cardiol. 2019, 16, 727. 31243391 10.1038/s41569-019-0227-9

[55] a) H. Pan , C. Xue , B. J. Auerbach , J. Fan , A. C. Bashore , J. Cui , D. Y. Yang , S. B. Trignano , W. Liu , J. Shi , C. O. Ihuegbu , E. C. Bush , J. Worley , L. Vlahos , P. Laise , R. A. Solomon , E. S. Connolly , A. Califano , P. A. Sims , H. Zhang , M. Li , M. P. Reilly , Circulation 2020, 142, 2060; 32962412 10.1161/CIRCULATIONAHA.120.048378 PMC8104264

[56] a) S. H. Zhang , R. L. Reddick , J. A. Piedrahita , N. Maeda , Science 1992, 258, 468; 1411543 10.1126/science.1411543

[57] a) F. A. Shelburne , S. H. Quarfordt , J. Biol. Chem. 1974, 249, 1428; 4361735

[58] a) F. Kuipers , J. M. van Ree , M. H. Hofker , H. Wolters , G. In't Veld , R. Havinga , R. J. Vonk , H. M. Princen , L. M. Havekes , Hepatology 1996, 24, 241; 8707269 10.1002/hep.510240138

[59] P. A. VanderLaan , C. A. Reardon , R. A. Thisted , G. S. Getz , J. Lipid Res. 2009, 50, 376. 18957695 10.1194/jlr.M800284-JLR200 PMC2638101

[60] D. Teupser , A. D. Persky , J. L. Breslow , Arterioscler., Thromb., Vasc. Biol. 2003, 23, 1907. 12907460 10.1161/01.ATV.0000090126.34881.B1

[61] S. Bonthu , D. D. Heistad , D. A. Chappell , K. G. Lamping , F. M. Faraci , Arterioscler., Thromb.,Vasc. Biol. 1997, 17, 2333. 9409199 10.1161/01.atv.17.11.2333

[62] B. J. van Vlijmen , A. M. van den Maagdenberg , M. J. Gijbels , H. van der Boom , H. HogenEsch , R. R. Frants , M. H. Hofker , L. M. Havekes , J. Clin. Invest. 1994, 93, 1403. 8163645 10.1172/JCI117117 PMC294153

[63] M. J. Gijbels , M. van der Cammen , L. J. van der Laan , J. J. Emeis , L. M. Havekes , M. H. Hofker , G. Kraal , Atherosclerosis 1999, 143, 15. 10208477 10.1016/s0021-9150(98)00263-9

[64] a) T. Sasaki , M. Kuzuya , K. Nakamura , X. W. Cheng , T. Shibata , K. Sato , A. Iguchi , Arterioscler., Thromb., Vasc. Biol. 2006, 26, 1304; 16574894 10.1161/01.ATV.0000219687.71607.f7

[65] J. L. Van Herck , G. R. Y. De Meyer , W. Martinet , C. E. Van Hove , K. Foubert , M. H. Theunis , S. Apers , H. Bult , C. J. Vrints , A. G. Herman , Circulation 2009, 120, 2478. 19948973 10.1161/CIRCULATIONAHA.109.872663

[66] W. A. Thomas , W. S. Hartroft , Circulation 1959, 19, 65. 13619021 10.1161/01.cir.19.1.65

[67] L. Yu , R. Gao , X. Song , X. Li , J. Zhu , J. Oleo Sci. 2021, 70, 1447. 34615830 10.5650/jos.ess21168

[68] M. Gao , G. Xin , X. Qiu , Y. Wang , G. Liu , J. Biomed. Res. 2016, 31, 47. 28808185 10.7555/JBR.31.20160020 PMC5274512

[69] S. D. Sithu , M. V. Malovichko , K. A. Riggs , N. S. Wickramasinghe , M. G. Winner , A. Agarwal , R. E. Hamed‐Berair , A. Kalani , D. W. Riggs , A. Bhatnagar , S. Srivastava , JCI Insight 2017, 2, e86442. 28469073 10.1172/jci.insight.86442 PMC5414561

[70] Y. Zhao , Y. Yang , R. Xing , X. Cui , Y. Xiao , L. Xie , P. You , T. Wang , L. Zeng , W. Peng , D. Li , H. Chen , M. Liu , Atherosclerosis 2018, 271, 26. 29459263 10.1016/j.atherosclerosis.2018.02.015

[71] J. Liang , E. Liu , Y. Yu , S. Kitajima , T. Koike , Y. Jin , M. Morimoto , K. Hatakeyama , Y. Asada , T. Watanabe , Y. Sasaguri , S. Watanabe , J. Fan , Circulation 2006, 113, 1993. 16636188 10.1161/CIRCULATIONAHA.105.596031

[72] Y. Watanabe , Atherosclerosis 1980, 36, 261. 7406953 10.1016/0021-9150(80)90234-8

[73] J. Fan , S. Kitajima , T. Watanabe , J. Xu , J. Zhang , E. Liu , Y. E. Chen , Pharmacol. Ther. 2015, 146, 104. 25277507 10.1016/j.pharmthera.2014.09.009 PMC4304984

[74] E. J. Keliher , Y.‐X. Ye , G. R. Wojtkiewicz , A. D. Aguirre , B. Tricot , M. L. Senders , H. Groenen , F. Fay , C. Perez‐Medina , C. Calcagno , G. Carlucci , T. Reiner , Y. Sun , G. Courties , Y. Iwamoto , H.‐Y. Kim , C. Wang , J. W. Chen , F. K. Swirski , H.‐Y. Wey , J. Hooker , Z. A. Fayad , W. J. M. Mulder , R. Weissleder , M. Nahrendorf , Nat. Commun. 2017, 8, 14064. 28091604 10.1038/ncomms14064 PMC5241815

[75] A. L. Al‐Mashhadi , C. B. Poulsen , K. von Wachenfeldt , A.‐K. Robertson , J. F. Bentzon , L. B. Nielsen , J. Thygesen , L. P. Tolbod , J. R. Larsen , S. K. Moestrup , B. Frendéus , B. Mortensen , L. Drouet , R. H. Al‐Mashhadi , E. Falk , J. Diabetes Res. 2018, 2018, 6823193. 29682581 10.1155/2018/6823193 PMC5845503

[76] D. Hamamdzic , R. L. Wilensky , J. Diabetes Res. 2013, 2013, 761415. 23844374 10.1155/2013/761415 PMC3697774

[77] M. Kim , H. B. Kim , D. S. Park , K. H. Cho , D. Y. Hyun , H. J. Kee , Y. J. Hong , M. H. Jeong , Sci. Rep. 2021, 11, 13695. 34211091 10.1038/s41598-021-93229-1 PMC8249376

[78] a) A. F. Hedayat , K. H. Park , T. G. Kwon , J. R. Woollard , K. Jiang , D. F. Carlson , A. Lerman , L. O. Lerman , Transl. Res. 2018, 192, 30; 29175268 10.1016/j.trsl.2017.10.007 PMC5811343

[79] J. R. Kaplan , S. B. Manuck , Ann. N. Y. Acad. Sci. 1999, 896, 145. 10681895 10.1111/j.1749-6632.1999.tb08112.x

[80] Y. Niu , B. Shen , Y. Cui , Y. Chen , J. Wang , L. Wang , Y. Kang , X. Zhao , W. Si , W. Li , A. P. Xiang , J. Zhou , X. Guo , Y. Bi , C. Si , B. Hu , G. Dong , H. Wang , Z. Zhou , T. Li , T. Tan , X. Pu , F. Wang , S. Ji , Q. Zhou , X. Huang , W. Ji , J. Sha , Cell 2014, 156, 836. 24486104 10.1016/j.cell.2014.01.027

[81] M. L. Senders , A. E. Meerwaldt , M. M. T. van Leent , B. L. Sanchez‐Gaytan , J. C. van de Voort , Y. C. Toner , A. Maier , E. D. Klein , N. A. T. Sullivan , A. M. Sofias , H. Groenen , C. Faries , R. S. Oosterwijk , E. M. van Leeuwen , F. Fay , E. Chepurko , T. Reiner , R. Duivenvoorden , L. Zangi , R. M. Dijkhuizen , S. Hak , F. K. Swirski , M. Nahrendorf , C. Pérez‐Medina , A. J. P. Teunissen , Z. A. Fayad , C. Calcagno , G. J. Strijkers , W. J. M. Mulder , Nat. Nanotechnol. 2020, 15, 398. 32313216 10.1038/s41565-020-0642-4 PMC7416336

[82] J. Tang , S. Baxter , A. Menon , A. Alaarg , B. L. Sanchez‐Gaytan , F. Fay , Y. Zhao , M. Ouimet , M. S. Braza , V. A. Longo , D. Abdel‐Atti , R. Duivenvoorden , C. Calcagno , G. Storm , S. Tsimikas , K. J. Moore , F. K. Swirski , M. Nahrendorf , E. A. Fisher , C. Pérez‐Medina , Z. A. Fayad , T. Reiner , W. J. M. Mulder , Proc. Natl. Acad. Sci. USA 2016, 113, E6731. 27791119 10.1073/pnas.1609629113 PMC5098679

[83] a) H. S. Choi , W. Liu , P. Misra , E. Tanaka , J. P. Zimmer , B. Itty Ipe , M. G. Bawendi , J. V. Frangioni , Nat. Biotechnol. 2007, 25, 1165; 17891134 10.1038/nbt1340 PMC2702539

[84] R. Taniguchi , Y. Miura , H. Koyama , T. Chida , Y. Anraku , A. Kishimura , K. Shigematsu , K. Kataoka , T. Watanabe , Mol. Pharmaceutics 2016, 13, 2108. 10.1021/acs.molpharmaceut.6b00219 27183493

[85] M. Suhara , Y. Miura , H. Cabral , D. Akagi , Y. Anraku , A. Kishimura , M. Sano , T. Miyazaki , N. Nakamura , A. Nishiyama , K. Kataoka , H. Koyama , K. Hoshina , J. Controlled Release 2018, 286, 394. 10.1016/j.jconrel.2018.07.049 30076876

[86] a) X. Hu , J. Hu , J. Tian , Z. Ge , G. Zhang , K. Luo , S. Liu , J. Am. Chem. Soc. 2013, 135, 17617; 24160840 10.1021/ja409686x

[87] a) S.‐Y. Lin , W.‐H. Hsu , J.‐M. Lo , H.‐C. Tsai , G.‐H. Hsiue , J. Controlled Release 2011, 154, 84; 10.1016/j.jconrel.2011.04.023 21565231

[88] a) N. Doshi , B. Prabhakarpandian , A. Rea‐Ramsey , K. Pant , S. Sundaram , S. Mitragotri , J. Controlled Release 2010, 146, 196; 10.1016/j.jconrel.2010.04.007 PMC4128960 20385181

[89] A. J. Thompson , E. M. Mastria , O. Eniola‐Adefeso , Biomaterials 2013, 34, 5863. 23642534 10.1016/j.biomaterials.2013.04.011

[90] A. M. Wen , Y. Wang , K. Jiang , G. C. Hsu , H. Gao , K. L. Lee , A. C. Yang , X. Yu , D. I. Simon , N. F. Steinmetz , J. Mater. Chem. B 2015, 3, 6037. 26509036 10.1039/C5TB00879D PMC4620043

[91] S. Yi , S. D. Allen , Y.‐G. Liu , B. Z. Ouyang , X. Li , P. Augsornworawat , E. B. Thorp , E. A. Scott , ACS Nano 2016, 10, 11290. 27935698 10.1021/acsnano.6b06451 PMC5418862

[92] J. Tang , M. Rakshit , H. M. Chua , A. Darwitan , L. T. H. Nguyen , A. Muktabar , S. Venkatraman , K. W. Ng , Nanotechnology 2021, 32, 505105. 10.1088/1361-6528/ac2810 34536952

[93] A. A. D'Souza , R. Shegokar , Expert Opin. Drug Delivery 2016, 13, 1257. 10.1080/17425247.2016.1182485 27116988

[94] a) M. Sarparanta , L. M. Bimbo , J. Rytkönen , E. Mäkilä , T. J. Laaksonen , P. Laaksonen , M. Nyman , J. Salonen , M. B. Linder , J. Hirvonen , H. A. Santos , A. J. Airaksinen , Mol. Pharmaceutics 2012, 9, 654; 10.1021/mp200611d 22277076

[95] a) C. Cao , L. Zhang , B. Kent , S. Wong , C. J. Garvey , M. H. Stenzel , Angew. Chem., Int. Ed. 2021, 60, 10342; 10.1002/anie.202101129 33543582

[96] a) X. Huang , C. Liu , N. Kong , Y. Xiao , A. Yurdagul , I. Tabas , W. Tao , Nat. Protoc. 2022, 17, 748; 35121853 10.1038/s41596-021-00665-4 PMC9734002

[97] S. Wang , Y. Wang , X. Lai , J. Sun , M. Hu , M. Chen , C. Li , F. Xu , C. Fan , X. Liu , Y. Song , G. Chen , Y. Deng , ACS Nano 2023, 17, 2761. 36719043 10.1021/acsnano.2c11058

[98] J. Yao , Z. Yang , L. Huang , C. Yang , J. Wang , Y. Cao , L. Hao , L. Zhang , J. Zhang , P. Li , Z. Wang , Y. Sun , H. Ran , Adv. Sci. 2021, 8, e2100850. 10.1002/advs.202100850 PMC8498883 34382370

[99] C. N. Morrell , D. N. Pariser , Z. T. Hilt , D. Vega Ocasio , Annu. Rev. Immunol. 2019, 37, 125. 30485751 10.1146/annurev-immunol-042718-041607

[100] C. P. Manta , T. Leibing , M. Friedrich , H. Nolte , M. Adrian , K. Schledzewski , J. Krzistetzko , C. Kirkamm , C. David Schmid , Y. Xi , A. Stojanovic , S. Tonack , C. de la Torre , S. Hammad , S. Offermanns , M. Krüger , A. Cerwenka , M. Platten , S. Goerdt , C. Géraud , Circulation 2022, 146, 1783. 36325910 10.1161/CIRCULATIONAHA.121.058615

[101] a) W. Chen , M. Schilperoort , Y. Cao , J. Shi , I. Tabas , W. Tao , Nat. Rev. Cardiol. 2022, 19, 228; 34759324 10.1038/s41569-021-00629-x PMC8580169

[102] H. B. Sager , P. Dutta , J. E. Dahlman , M. Hulsmans , G. Courties , Y. Sun , T. Heidt , C. Vinegoni , A. Borodovsky , K. Fitzgerald , G. R. Wojtkiewicz , Y. Iwamoto , B. Tricot , O. F. Khan , K. J. Kauffman , Y. Xing , T. E. Shaw , P. Libby , R. Langer , R. Weissleder , F. K. Swirski , D. G. Anderson , M. Nahrendorf , Sci. Transl. Med. 2016, 8, 342ra80. 10.1126/scitranslmed.aaf1435 PMC5125383 27280687

[103] A. J. Beer , J. Pelisek , P. Heider , A. Saraste , C. Reeps , S. Metz , S. Seidl , H. Kessler , H. J. Wester , H. H. Eckstein , M. Schwaiger , JACC: Cardiovasc. Imaging 2014, 7, 178. 24412187 10.1016/j.jcmg.2013.12.003

[104] R. Mohindra , D. K. Agrawal , F. G. Thankam , J. Cardiovasc. Transl. Res. 2021, 14, 647. 33420681 10.1007/s12265-020-10091-8

[105] T. Weiss‐Sadan , Y. Ben‐Nun , D. Maimoun , E. Merquiol , I. Abd‐Elrahman , I. Gotsman , G. Blum , Theranostics 2019, 9, 5731. 31534515 10.7150/thno.34402 PMC6735363

[106] N. Cheraga , Z. Ye , M. J. Xu , L. Zou , N. C. Sun , Y. Hang , C. J. Shan , Z. Z. Yang , L. J. Chen , N. P. Huang , Nanoscale 2022, 14, 8709. 35673987 10.1039/d1nr06514a

[107] H. Xu , P. Y. She , B. X. Ma , Z. Y. Zhao , G. C. Li , Y. B. Wang , Biomaterials 2022, 288, 121734. 35999079 10.1016/j.biomaterials.2022.121734

[108] K. Song , Z. Tang , Z. Song , S. Meng , X. Yang , H. Guo , Y. Zhu , X. Wang , Pharmaceutics 2022, 14, 1265. 35745836 10.3390/pharmaceutics14061265 PMC9227583

[109] Y. Huang , T. Li , W. Gao , Q. Wang , X. Li , C. Mao , M. Zhou , M. Wan , J. Shen , J. Mater. Chem. B 2020, 8, 5765. 32519722 10.1039/d0tb00789g

[110] a) A. Dunphy , K. Patel , S. Belperain , A. Pennington , N. H. L. Chiu , Z. Y. Yin , X. W. Zhu , B. Priebe , S. M. Tian , J. J. Wei , X. W. Yi , Z. Q. Jia , Nanomaterials 2021, 11, 1116; 33925858 10.3390/nano11051116 PMC8145848

[111] M. Gifani , D. J. Eddins , H. Kosuge , Y. Zhang , S. L. A. Paluri , T. Larson , N. Leeper , L. A. Herzenberg , S. S. Gambhir , M. V. McConnell , E. E. B. Ghosn , B. R. Smith , Adv. Funct. Mater. 2021, 31, 2101005. 34733130 10.1002/adfm.202101005 PMC8559995

[112] J. Liu , B. Zhou , Y. Guo , A. Zhang , K. Yang , Y. He , J. Wang , Y. Cheng , D. Cui , ACS Appl. Mater. Interfaces 2021, 13, 29349. 10.1021/acsami.1c06380 34133141

[113] B. Oh , C. H. Lee , Mol. Pharmaceutics 2015, 12, 3226. 10.1021/acs.molpharmaceut.5b00181 26161461

[114] a) W. Tao , O. C. Farokhzad , Chem. Rev. 2022, 122, 5405; 35317560 10.1021/acs.chemrev.2c00089

[115] J. Jia , G. Y. Liu , W. J. Xu , X. L. Tian , S. B. Li , F. Han , Y. H. Feng , X. C. Dong , H. Y. Chen , Angew. Chem., Int. Ed. 2020, 59, 14443. 10.1002/anie.202000474 32476215

[116] C. Wang , X.‐G. Nie , Y. Shi , Y. Zhou , J.‐J. Xu , X.‐H. Xia , H.‐Y. Chen , ACS Nano 2017, 11, 5897. 28494145 10.1021/acsnano.7b01637

[117] a) S. Bhagat , N. V. Srikanth Vallabani , V. Shutthanandan , M. Bowden , A. S. Karakoti , S. Singh , J. Colloid Interface Sci. 2018, 513, 831; 29223890 10.1016/j.jcis.2017.11.064

[118] B. B. Karakoçak , A. Laradji , T. Primeau , M. Y. Berezin , S. Li , N. Ravi , ACS Appl. Mater. Interfaces 2021, 13, 277. 33355448 10.1021/acsami.0c20088 PMC8243741

[119] a) W. Feng , X. Han , H. Hu , M. Chang , L. Ding , H. Xiang , Y. Chen , Y. Li , Nat. Commun. 2021, 12, 2203; 33850133 10.1038/s41467-021-22278-x PMC8044242

[120] X. Ge , H. Cui , J. Kong , S.‐Y. Lu , R. Zhan , J. Gao , Y. Xu , S. Lin , K. Meng , L. Zu , S. Guo , L. Zheng , Adv. Mater. 2020, 32, 2000037. 10.1002/adma.202000037 32803803

[121] a) D. E. Large , R. G. Abdelmessih , E. A. Fink , D. T. Auguste , Adv. Drug Delivery Rev. 2021, 176, 113851; 10.1016/j.addr.2021.113851 34224787

[122] S. Jebari‐Benslaiman , K. B. Uribe , A. Benito‐Vicente , U. Galicia‐Garcia , A. Larrea‐Sebal , I. Santin , I. Alloza , K. Vandenbroeck , H. Ostolaza , C. Martín , Small 2022, 18, e2105915. 35156292 10.1002/smll.202105915

[123] D. Gigmes , T. Trimaille , Adv. Colloid Interface Sci. 2021, 294, 102483. 34274723 10.1016/j.cis.2021.102483

[124] F. Fang , Y. H. Ni , H. C. Yu , H. M. Yin , F. Yang , C. L. Li , D. L. Sun , T. Pei , J. Ma , L. Deng , H. Y. Zhang , G. X. Wang , S. Li , Y. Shen , X. H. Liu , Theranostics 2022, 12, 4200. 35673565 10.7150/thno.70896 PMC9169363

[125] M. Yousefi , A. Narmani , S. M. Jafari , Adv. Colloid Interface Sci. 2020, 278, 102125. 32109595 10.1016/j.cis.2020.102125

[126] S. Li , L. Wang , Y. Gu , L. Lin , M. Zhang , M. Jin , C. Mao , J. Zhou , W. Zhang , X. Huang , C. Corbo , W. Tao , E. Lu , J. Liu , Matter 2021, 4, 3621.

[127] Y. Wang , K. Zhang , X. Qin , T. Li , J. Qiu , T. Yin , J. Huang , S. McGinty , G. Pontrelli , J. Ren , Q. Wang , W. Wu , G. Wang , Adv. Sci. 2019, 6, 1900172. 10.1002/advs.201900172 PMC6662054 31380165

[128] J. Shi , L. Kundrat , N. Pishesha , A. Bilate , C. Theile , T. Maruyama , S. K. Dougan , H. L. Ploegh , H. F. Lodish , Proc. Natl. Acad. Sci. USA 2014, 111, 10131. 24982154 10.1073/pnas.1409861111 PMC4104923

[129] W. Gao , H. Yang , X. Liu , Z. Liu , L. Tong , Y. Sun , W. Cao , Y. Cao , B. Tang , Chem. Commun. 2019, 55, 11535. 10.1039/c9cc06039a 31490499

[130] L. Chen , Z. Zhou , C. Hu , M. F. Maitz , L. Yang , R. Luo , Y. Wang , Research 2022, 2022, 9845459. 35118420 10.34133/2022/9845459 PMC8791388

[131] Y. Wang , K. Zhang , T. H. Li , A. Maruf , X. Qin , L. Luo , Y. Zhong , J. H. Qiu , S. McGinty , G. Pontrelli , X. L. Liao , W. Wu , G. X. Wang , Theranostics 2021, 11, 164. 33391468 10.7150/thno.47841 PMC7681077

[132] Z. Zhang , J. Zhou , C. Liu , J. Zhang , Y. Shibata , N. Kong , C. Corbo , M. B. Harris , W. Tao , Trends Chem. 2022, 4, 420.

[133] C. Gao , Q. X. Huang , C. H. Liu , C. H. T. Kwong , L. D. Yue , J. B. Wan , S. M. Y. Lee , R. B. Wang , Nat. Commun. 2020, 11, 2622. 32457361 10.1038/s41467-020-16439-7 PMC7251120

[134] J. M. Tarkin , M. R. Dweck , N. R. Evans , R. A. P. Takx , A. J. Brown , A. Tawakol , Z. A. Fayad , J. H. F. Rudd , Circ. Res. 2016, 118, 750. 26892971 10.1161/CIRCRESAHA.115.306247 PMC4756468

[135] R. Di Corato , F. Gazeau , C. Le Visage , D. Fayol , P. Levitz , F. Lux , D. Letourneur , N. Luciani , O. Tillement , C. Wilhelm , ACS Nano 2013, 7, 7500. 23924160 10.1021/nn401095p

[136] E. Gallo , C. Diaferia , E. D. Gregorio , G. Morelli , E. Gianolio , A. Accardo , Pharmaceuticals 2020, 13, 19. 31973215 10.3390/ph13020019 PMC7168922

[137] R. Chen , D. Ling , L. Zhao , S. Wang , Y. Liu , R. Bai , S. Baik , Y. Zhao , C. Chen , T. Hyeon , ACS Nano 2015, 9, 12425. 26567968 10.1021/acsnano.5b05783

[138] a) P. Caravan , J. J. Ellison , T. J. McMurry , R. B. Lauffer , Chem. Rev. 1999, 99, 2293; 11749483 10.1021/cr980440x

[139] F. Capuana , A. Phinikaridou , R. Stefania , S. Padovan , B. Lavin , S. Lacerda , E. Almouazen , Y. Chevalier , L. Heinrich‐Balard , R. M. Botnar , S. Aime , G. Digilio , J. Med. Chem. 2021, 64, 15250. 34661390 10.1021/acs.jmedchem.1c01286 PMC8558862

[140] T. M. Cheng , R. Li , Y. C. J. Kao , C. H. Hsu , H. L. Chu , K. Y. Lu , C. A. Changou , C. C. Chang , L. H. Chang , M. L. Tsai , F. L. Mi , Mater. Sci. Eng.: C 2020, 114, 111064. 10.1016/j.msec.2020.111064 32994013

[141] M. R. Makowski , G. Varma , A. J. Wiethoff , A. Smith , K. Mattock , C. H. P. Jansen , A. Warley , M. Taupitz , T. Schaeffter , R. M. Botnar , Circ.: Cardiovasc. Imaging 2011, 4, 295. 21422166 10.1161/CIRCIMAGING.110.957209

[142] H. Qiao , Y. Wang , R. Zhang , Q. Gao , X. Liang , L. Gao , Z. Jiang , R. Qiao , D. Han , Y. Zhang , Y. Qiu , J. Tian , M. Gao , F. Cao , Biomaterials 2017, 112, 336. 27788352 10.1016/j.biomaterials.2016.10.011

[143] X. Huang , C. Lin , C. Luo , Y. Guo , J. Li , Y. Wang , J. Xu , Y. Zhang , H. Wang , Z. Liu , B. Chen , Nanomedicine 2021, 33, 102348. 33321215 10.1016/j.nano.2020.102348

[144] S. Hossaini Nasr , A. Tonson , M. H. El‐Dakdouki , D. C. Zhu , D. Agnew , R. Wiseman , C. Qian , X. Huang , ACS Appl. Mater. Interfaces 2018, 10, 11495. 29558108 10.1021/acsami.7b19708 PMC5995107

[145] A. Yilmaz , M. A. Dengler , H. van der Kuip , H. Yildiz , S. Rösch , S. Klumpp , K. Klingel , R. Kandolf , X. Helluy , K.‐H. Hiller , P. M. Jakob , U. Sechtem , Eur. Heart J. 2013, 34, 462. 23103659 10.1093/eurheartj/ehs366

[146] a) W. H. Kong , W. J. Lee , Z. Y. Cui , K. H. Bae , T. G. Park , J. H. Kim , K. Park , S. W. Seo , Biomaterials 2007, 28, 5555; 17904632 10.1016/j.biomaterials.2007.08.044

[147] D. B. Elrod , R. Partha , D. Danila , S. W. Casscells , J. L. Conyers , Nanomedicine 2009, 5, 42. 18783999 10.1016/j.nano.2008.06.007

[148] S. You , H.‐Y. Jung , C. Lee , Y. H. Choe , J. Y. Heo , G.‐T. Gang , S.‐K. Byun , W. K. Kim , C.‐H. Lee , D.‐E. Kim , Y. I. Kim , Y. Kim , J. Controlled Release 2016, 226, 258. 10.1016/j.jconrel.2016.01.036 26812006

[149] Q. Yin , F. Y. Yap , L. Yin , L. Ma , Q. Zhou , L. W. Dobrucki , T. M. Fan , R. C. Gaba , J. Cheng , J. Am. Chem. Soc. 2013, 135, 13620. 23987119 10.1021/ja405196f PMC4232444

[150] a) F. Hyafil , J.‐C. Cornily , J. E. Feig , R. Gordon , E. Vucic , V. Amirbekian , E. A. Fisher , V. Fuster , L. J. Feldman , Z. A. Fayad , Nat. Med. 2007, 13, 636; 17417649 10.1038/nm1571

[151] J. L. Van Herck , G. R. Y. De Meyer , W. Martinet , R. A. Salgado , B. Shivalkar , R. De Mondt , H. Van De Ven , A. Ludwig , P. Van Der Veken , L. Van Vaeck , H. Bult , A. G. Herman , C. J. Vrints , Basic Res. Cardiol. 2010, 105, 51. 19693628 10.1007/s00395-009-0052-0

[152] D. P. Cormode , E. Roessl , A. Thran , T. Skajaa , R. E. Gordon , J.‐P. Schlomka , V. Fuster , E. A. Fisher , W. J. M. Mulder , R. Proksa , Z. A. Fayad , Radiology 2010, 256, 774. 20668118 10.1148/radiol.10092473 PMC2923725

[153] J.‐Y. Kim , J. H. Ryu , D. Schellingerhout , I.‐C. Sun , S.‐K. Lee , S. Jeon , J. Kim , I. C. Kwon , M. Nahrendorf , C.‐H. Ahn , K. Kim , D.‐E. Kim , Theranostics 2015, 5, 1098. 26199648 10.7150/thno.11679 PMC4508499

[154] P. Chhour , P. C. Naha , S. M. O'Neill , H. I. Litt , M. P. Reilly , V. A. Ferrari , D. P. Cormode , Biomaterials 2016, 87, 93. 26914700 10.1016/j.biomaterials.2016.02.009 PMC4783300

[155] O. Lee , S. Kappler , C. Polster , K. Taguchi , IEEE Trans. Med. Imaging 2017, 36, 2389. 28866486 10.1109/TMI.2017.2746269

[156] S. A. Si‐Mohamed , M. Sigovan , J. C. Hsu , V. Tatard‐Leitman , L. Chalabreysse , P. C. Naha , T. Garrivier , R. Dessouky , M. Carnaru , L. Boussel , D. P. Cormode , P. C. Douek , Radiology 2021, 300, 98. 33944628 10.1148/radiol.2021203968 PMC8217298

[157] A. Irkle , A. T. Vesey , D. Y. Lewis , J. N. Skepper , J. L. E. Bird , M. R. Dweck , F. R. Joshi , F. A. Gallagher , E. A. Warburton , M. R. Bennett , K. M. Brindle , D. E. Newby , J. H. Rudd , A. P. Davenport , Nat. Commun. 2015, 6, 7495. 26151378 10.1038/ncomms8495 PMC4506997

[158] P. Nogales , C. Velasco , A. Mota‐Cobián , L. González‐Cintado , R. A. Mota , S. España , J. Mateo , J. F. Bentzon , Arterioscler., Thromb., Vasc. Biol. 2021, 41, e480. 34289703 10.1161/ATVBAHA.121.316075

[159] a) Z. Varasteh , F. De Rose , S. Mohanta , Y. Li , X. Zhang , B. Miritsch , G. Scafetta , C. Yin , H. B. Sager , S. Glasl , D. Gorpas , A. J. R. Habenicht , V. Ntziachristos , W. A. Weber , A. Bartolazzi , M. Schwaiger , C. D'Alessandria , Theranostics 2021, 11, 1864; 33408786 10.7150/thno.50247 PMC7778602

[160] J. Pellico , I. Fernández‐Barahona , J. Ruiz‐Cabello , L. Gutiérrez , M. Muñoz‐Hernando , M. J. Sánchez‐Guisado , I. Aiestaran‐Zelaia , L. Martínez‐Parra , I. Rodríguez , J. Bentzon , F. Herranz , ACS Appl. Mater. Interfaces 2021, 13, 45279. 34529427 10.1021/acsami.1c13417 PMC8485330

[161] M. E. Lobatto , T. Binderup , P. M. Robson , L. F. P. Giesen , C. Calcagno , J. Witjes , F. Fay , S. Baxter , C. H. Wessel , M. Eldib , J. Bini , S. D. Carlin , E. S. G. Stroes , G. Storm , A. Kjaer , J. S. Lewis , T. Reiner , Z. A. Fayad , W. J. M. Mulder , C. Pérez‐Medina , Bioconjugate Chem. 2020, 31, 360. 10.1021/acs.bioconjchem.9b00256 PMC7460274 31095372

[162] L. Detering , A. Abdilla , H. P. Luehmann , J. W. Williams , L.‐H. Huang , D. Sultan , A. Elvington , G. S. Heo , P. K. Woodard , R. J. Gropler , G. J. Randolph , C. J. Hawker , Y. Liu , Mol. Pharmaceutics 2021, 18, 1386. 10.1021/acs.molpharmaceut.0c01183 PMC8737066 33591187

[163] M. Liang , H. Tan , J. Zhou , T. Wang , D. Duan , K. Fan , J. He , D. Cheng , H. Shi , H. S. Choi , X. Yan , ACS Nano 2018, 12, 9300. 30165015 10.1021/acsnano.8b04158

[164] W.‐L. Wu , H.‐L. Ma , L.‐L. Xi , M.‐F. Huang , K.‐M. Wang , J.‐Y. Miao , B.‐X. Zhao , Talanta 2019, 194, 308. 30609535 10.1016/j.talanta.2018.10.006

[165] F. Maafi , B. Li , C. Gebhard , M. R. Brodeur , W. Nachar , L. Villeneuve , F. Lesage , D. Rhainds , E. Rhéaume , J.‐C. Tardif , Atherosclerosis 2017, 258, 8. 28167355 10.1016/j.atherosclerosis.2017.01.026

[166] Q. Wang , R. Lou , Q. Yin , R. Yang , S. Li , J. Zhou , Analyst 2021, 146, 4674. 34190228 10.1039/d1an00484k

[167] J.‐A. Chen , W. Guo , Z. Wang , N. Sun , H. Pan , J. Tan , Z. Ouyang , W. Fu , Y. Wang , W. Hu , X. Gu , Anal. Chem. 2020, 92, 12613. 32786453 10.1021/acs.analchem.0c02670

[168] B. Wang , F. Zhang , S. Wang , R. Yang , C. Chen , W. Zhao , Chem. Commun. 2020, 56, 2598. 10.1039/c9cc07256j 32016205

[169] H. Ikeda , A. Ishii , K. Sano , H. Chihara , D. Arai , Y. Abekura , H. Nishi , M. Ono , H. Saji , S. Miyamoto , Atherosclerosis 2018, 275, 1. 29852399 10.1016/j.atherosclerosis.2018.05.028

[170] a) M. S. Albaghdadi , R. Ikegami , M. B. Kassab , J. A. Gardecki , M. Kunio , M. M. Chowdhury , R. Khamis , P. Libby , G. J. Tearney , F. A. Jaffer , Arterioscler., Thromb., Vasc. Biol. 2021, 41, e385; 34011166 10.1161/ATVBAHA.120.315612 PMC8222195

[171] a) S.‐A. Manea , M.‐L. Vlad , D. Rebleanu , A.‐G. Lazar , I. M. Fenyo , M. Calin , M. Simionescu , A. Manea , Oxid. Med. Cell. Longevity 2021, 2021, 6685612; 10.1155/2021/6685612 PMC7963910 33763173

[172] Y. Narita , K. Shimizu , K. Ikemoto , R. Uchino , M. Kosugi , M. B. Maess , Y. Magata , N. Oku , M. Ogawa , J. Controlled Release 2019, 302, 105. 10.1016/j.jconrel.2019.03.025 30936020

[173] K. Wang , H. Q. Gao , Y. W. Zhang , H. Y. Yan , J. H. Si , X. Y. Mi , S. A. Xia , X. Q. Feng , D. B. Liu , D. L. Kong , T. Wang , D. Ding , Adv. Mater. 2022, 34, 2106994. 10.1002/adma.202106994 34921573

[174] R. Qiao , H. Qiao , Y. Zhang , Y. Wang , C. Chi , J. Tian , L. Zhang , F. Cao , M. Gao , ACS Nano 2017, 11, 1816. 28121134 10.1021/acsnano.6b07842

[175] X. Sun , W. Li , X. Zhang , M. Qi , Z. Zhang , X.‐E. Zhang , Z. Cui , Nano Lett. 2016, 16, 6164. 27622963 10.1021/acs.nanolett.6b02386

[176] E. C. Ximendes , U. Rocha , B. del Rosal , A. Vaquero , F. Sanz‐Rodríguez , L. Monge , F. Ren , F. Vetrone , D. Ma , J. García‐Solé , C. Jacinto , D. Jaque , N. Fernández , Adv. Healthcare Mater. 2017, 6, 1601195. 10.1002/adhm.201601195 28009096

[177] H. P. Deng , C. J. Konopka , S. Prabhu , S. Sarkar , N. G. Medina , M. Fayyaz , O. H. Arogundade , H. E. V. Gamage , S. H. Shahoei , D. Nall , Y. Youn , I. T. Dobrucka , C. O. Audu , A. Joshi , W. J. Melvin , K. A. Gallagher , P. R. Selvin , E. R. Nelson , L. W. Dobrucki , K. S. Swanson , A. M. Smith , ACS Nano 2022, 16, 1999. 35107994 10.1021/acsnano.1c07010 PMC8900655

[178] L. V. Wang , S. Hu , Science 2012, 335, 1458. 22442475 10.1126/science.1216210 PMC3322413

[179] B. Wang , J. L. Su , J. Amirian , S. H. Litovsky , R. Smalling , S. Emelianov , Opt. Express 2010, 18, 4889. 20389501 10.1364/OE.18.004889 PMC3378348

[180] Y. Cao , A. Kole , L. Lan , P. Wang , J. Hui , M. Sturek , J.‐X. Cheng , J. Photoacoust. 2017, 7, 12. 10.1016/j.pacs.2017.05.002 PMC5472148 28649497

[181] B. R. Smith , E. E. B. Ghosn , H. Rallapalli , J. A. Prescher , T. Larson , L. A. Herzenberg , S. S. Gambhir , Nat. Nanotechnol. 2014, 9, 481. 24727688 10.1038/nnano.2014.62 PMC4236538

[182] Z. Xie , Y. Yang , Y. He , C. Shu , D. Chen , J. Zhang , J. Chen , C. Liu , Z. Sheng , H. Liu , J. Liu , X. Gong , L. Song , S. Dong , Theranostics 2020, 10, 4694. 32292523 10.7150/thno.41211 PMC7150488

[183] Y. Ma , L. Xu , B. Yin , J. Shang , F. Chen , J. Xu , Z.‐L. Song , B. Nan , G. Song , X.‐B. Zhang , Nano Lett. 2021, 21, 4484. 33978427 10.1021/acs.nanolett.1c01359

[184] M. Araki , S. J. Park , H. L. Dauerman , S. Uemura , J. S. Kim , C. Di Mario , T. W. Johnson , G. Guagliumi , A. Kastrati , M. Joner , N. R. Holm , F. Alfonso , W. Wijns , T. Adriaenssens , H. Nef , G. Rioufol , N. Amabile , G. Souteyrand , N. Meneveau , E. Gerbaud , M. P. Opolski , N. Gonzalo , G. J. Tearney , B. Bouma , A. D. Aguirre , G. S. Mintz , G. W. Stone , C. V. Bourantas , L. Räber , S. Gili , et al., Nat. Rev. Cardiol. 2022, 19, 684. 35449407 10.1038/s41569-022-00687-9 PMC9982688

[185] J. Hu , F. Rivero , R. A. Torres , H. Loro Ramírez , E. M. Rodríguez , F. Alfonso , J. García Solé , D. Jaque , J. Biophotonics 2017, 10, 674. 27273138 10.1002/jbio.201600062

[186] J. Hu , F. Sanz‐Rodríguez , F. Rivero , E. M. Rodríguez , R. A. Torres , D. H. Ortgies , J. G. Solé , F. Alfonso , D. Jaque , Nano Res. 2018, 11, 676.

[187] J. Hu , D. H. Ortgies , R. Aguliar Torres , N. Fernández , L. Porto , E. Martín Rodríguez , J. García Solé , D. Jaque , F. Alfonso , F. Rivero , Adv. Funct. Mater. 2017, 27, 1703276.

[188] R. Chaudhary , K. Roy , R. K. Kanwar , K. Walder , J. R. Kanwar , J. Nanobiotechnol. 2016, 14, 6. 10.1186/s12951-016-0157-1 PMC4715323 26775253

[189] M. Larivière , C. S. Lorenzato , L. Adumeau , S. Bonnet , A. Hémadou , M.‐J. Jacobin‐Valat , A. Noubhani , X. Santarelli , L. Minder , C. Di Primo , S. Sanchez , S. Mornet , J. Laroche‐Traineau , G. Clofent‐Sanchez , Nanomed.: Nanotechnol. Biol. Med. 2019, 22, 102082. 10.1016/j.nano.2019.102082 31404651

[190] M. Wu , X. Li , Q. Guo , J. Li , G. Xu , G. Li , J. Wang , X. Zhang , Nanomed.: Nanotechnol. Biol. Med. 2021, 32, 102330. 10.1016/j.nano.2020.102330 33171287

[191] W. Tong , H. Hui , W. Shang , Y. Zhang , F. Tian , Q. Ma , X. Yang , J. Tian , Y. Chen , Theranostics 2021, 11, 506. 33391489 10.7150/thno.49812 PMC7738857

[192] O. Will , S. Purkayastha , C. Chan , T. Athanasiou , A. W. Darzi , W. Gedroyc , P. P. Tekkis , Lancet Oncol. 2006, 7, 52. 16389184 10.1016/S1470-2045(05)70537-4

[193] a) S. P. Howarth , T. Y. Tang , R. Trivedi , R. Weerakkody , J. M. U‐King‐Im , M. E. Gaunt , J. R. Boyle , Z. Y. Li , S. R. Miller , M. J. Graves , J. H. Gillard , Eur. J. Radiol. 2009, 70, 555; 18356000 10.1016/j.ejrad.2008.01.047

[194] L. P. Smits , F. Tiessens , K. H. Zheng , E. S. Stroes , A. J. Nederveen , B. F. Coolen , Atherosclerosis 2017, 263, 211. 28662398 10.1016/j.atherosclerosis.2017.06.020

[195] K. H. Zheng , J. Schoormans , L. C. A. Stiekema , C. Calcagno , I. Cicha , C. Alexiou , G. J. Strijkers , A. J. Nederveen , E. S. G. Stroes , B. F. Coolen , JACC: Cardiovasc. Imaging 2019, 12, 2081. 31202746 10.1016/j.jcmg.2019.04.014

[196] A. Usman , A. J. Patterson , J. Yuan , A. Cluroe , I. Patterson , M. J. Graves , J. H. Gillard , U. Sadat , Sci. Rep. 2020, 10, 1808. 32020031 10.1038/s41598-020-58708-x PMC7000763

[197] a) C. G. Stirrat , S. R. Alam , T. J. MacGillivray , C. D. Gray , R. Forsythe , M. R. Dweck , J. R. Payne , S. K. Prasad , M. C. Petrie , R. S. Gardner , S. Mirsadraee , P. A. Henriksen , D. E. Newby , S. I. Semple , J. Cardiovasc. Magn. Reson. 2016, 18, 46; 27465647 10.1186/s12968-016-0261-2 PMC4964058

[198] a) S. Y. Chong , X. Wang , L. van Bloois , C. Huang , N. S. Syeda , S. Zhang , H. J. Ting , V. Nair , Y. Lin , C. K. L. Lou , A. A. Benetti , X. Yu , N. J. Y. Lim , M. S. Tan , H. Y. Lim , S. Y. Lim , C. H. Thiam , W. D. Looi , O. Zharkova , N. W. S. Chew , C. H. Ng , G. K. Bonney , M. Muthiah , X. Chen , G. Pastorin , A. M. Richards , V. Angeli , G. Storm , J. W. Wang , J. Controlled Release 2023, 360, 344; 10.1016/j.jconrel.2023.06.035 37406819

[199] a) Y. Chen , D. Qin , J. Zou , X. Li , X. D. Guo , Y. Tang , C. Liu , W. Chen , N. Kong , C. Y. Zhang , W. Tao , Adv. Mater. 2023, 35, 2207787; 10.1002/adma.202207787 36317596

[200] a) D. D. Chin , N. Patel , W. Lee , S. Kanaya , J. Cook , E. J. Chung , Bioact. Mater. 2023, 27, 327; 37122900 10.1016/j.bioactmat.2023.04.001 PMC10140752

[201] B. Boersma , K. Möller , L. Wehl , V. Puddinu , A. Huard , S. Fauteux‐Daniel , C. Bourquin , G. Palmer , T. Bein , J. Controlled Release 2022, 351, 989. 10.1016/j.jconrel.2022.09.063 36202154

[202] Y. Liu , M. He , Y. Yuan , C. Nie , K. Wei , T. Zhang , T. Chen , X. Chu , ACS Nano 2023, 17, 7721. 37023215 10.1021/acsnano.3c00288

[203] J. Wan , J. Yang , W. R. Lei , Z. Z. Xiao , P. Y. Zhou , S. Y. Zheng , P. Zhu , Int. J. Nanomed. 2023, 18, 579. 10.2147/IJN.S384675 PMC9901454 36756051

[204] M. Rakshit , A. Darwitan , A. Muktabar , P. Das , L. T. H. Nguyen , Y. Cao , C. Vizetto‐Duarte , J. K. Tang , Y. S. Wong , S. Venkatraman , K. W. Ng , Nanomed.: Nanotechnol. Biol. Med. 2021, 37, 102434. 10.1016/j.nano.2021.102434 34214684

[205] N. Benne , R. M. Cardoso , A. L. Boyle , A. Kros , W. Jiskoot , J. Kuiper , J. Bouwstra , M. Van Eck , B. Slutter , Adv. Healthcare Mater. 2020, 9, 2000043. 10.1002/adhm.202000043 32329226

[206] a) X. Jia , X. Bai , X. Yang , L. Wang , Y. Lu , L. Zhu , Y. Zhao , W. Cheng , M. Shu , Q. Mei , S. Jin , Metabolism 2022, 135, 155274; 35917895 10.1016/j.metabol.2022.155274

[207] Y. Wu , Y. Zhang , L. Dai , Q. Wang , L. Xue , Z. Su , C. Zhang , J. Controlled Release 2019, 316, 236. 10.1016/j.jconrel.2019.10.043 31672624

[208] Y. Song , Z. Huang , X. Liu , Z. Pang , J. Chen , H. Yang , N. Zhang , Z. Cao , M. Liu , J. Cao , C. Li , X. Yang , H. Gong , J. Qian , J. Ge , Nanomedicine 2019, 15, 13. 30171903 10.1016/j.nano.2018.08.002

[209] Y. N. Song , N. Zhang , Q. Y. Li , J. Chen , Q. Z. Wang , H. B. Yang , H. P. Tan , J. F. Gao , Z. H. Dong , Z. Q. Pang , Z. Y. Huang , J. Y. Qian , J. B. Ge , Chem. Eng. J. 2021, 408, 127296.

[210] J. X. Liu , Y. Y. Yang , X. Liu , A. S. Widjaya , B. H. Jiang , Y. Y. Jiang , J. Controlled Release 2021, 337, 224. 10.1016/j.jconrel.2021.07.032 34298057

[211] X. Sha , Y. Dai , L. J. Chong , M. Wei , M. Y. Xing , C. Zhang , J. J. Li , J. Nanobiotechnol. 2022, 20, 506. 10.1186/s12951-022-01720-2 PMC9714205 36456996

[212] Y. Li , J. Che , L. Chang , M. Guo , X. Bao , D. Mu , X. Sun , X. Zhang , W. Lu , J. Xie , Adv. Healthcare Mater. 2022, 11, 2101788. 10.1002/adhm.202101788 34786845

[213] M. L. Shen , S. Y. Yao , S. J. Li , X. D. Wu , S. Liu , Q. B. Yang , J. S. Du , J. Y. Wang , X. Y. Zheng , Y. P. Li , Nanoscale 2021, 13, 20013. 34842887 10.1039/d1nr05355h

[214] C. Gao , C. H. Liu , Q. Chen , Y. Wang , C. H. T. Kwong , Q. F. Wang , B. B. Xie , S. M. Y. Lee , R. B. Wang , J. Controlled Release 2022, 349, 2. 10.1016/j.jconrel.2022.06.053 35779655

[215] Z. J. Li , Q. Wang , H. S. Jing , X. H. Luo , L. F. Du , Y. R. Duan , ACS Appl. Nano Mater. 2021, 4, 11554.

[216] a) R. Zhao , X. Ning , M. Wang , H. Wang , G. Xing , L. Wang , C. Lu , A. Yu , Y. Wang , ACS Appl. Mater. Interfaces 2022, 14, 25080; 35618653 10.1021/acsami.2c02354

[217] M. R. Guo , Z. S. He , Z. H. Jin , L. J. Huang , J. M. Yuan , S. G. Qin , X. C. Wang , L. L. Cao , X. R. Song , Nano Res. 2022, 16, 925.

[218] a) X. Y. Li , R. Wu , H. Chen , T. Li , H. M. Jiang , X. Q. Xu , X. T. Tang , M. Wan , C. Mao , D. Q. Shi , ACS Appl. Mater. Interfaces 2021, 13, 30930; 34156244 10.1021/acsami.1c03600

[219] H. J. Pu , M. H. Yao , Z. Y. Wu , Z. J. Xu , C. Y. Cui , R. H. Huang , M. Shafiq , W. M. Li , X. W. Lu , B. Li , Nano Res. 2022, 15, 7342.

[220] W. D. Sun , Y. Y. Xu , Y. Yao , J. Yue , Z. Wu , H. C. Li , G. H. Shen , Y. Liao , H. Y. Wang , W. H. Zhou , J. Nanobiotechnol. 2022, 20, 88. 10.1186/s12951-022-01296-x PMC8858544 35183183

[221] Z. J. Xu , Z. Y. Wu , S. Huang , K. C. Ye , Y. H. Jiang , J. Q. Liu , J. C. Liu , X. W. Lu , B. Li , J. Controlled Release 2023, 354, 615. 10.1016/j.jconrel.2023.01.024 36641123

[222] X. Zhu , H. Wang , L. Zheng , Z. Zhong , X. Li , J. Zhao , J. Kou , Y. Jiang , X. Zheng , Z. Liu , H. Li , W. Cao , Y. Tian , Y. Wang , L. Yang , Int. J. Nanomed. 2015, 10, 3719. 10.2147/IJN.S82162 PMC4447170 26045663

[223] L. Zou , Y. Zhang , N. Cheraga , O. D. Abodunrin , K.‐Y. Qu , L. Qiao , Y.‐Q. Ma , L.‐J. Chen , N.‐P. Huang , Talanta 2023, 265, 124772. 37327664 10.1016/j.talanta.2023.124772

[224] M. Xu , C. Mao , H. Chen , L. Liu , Y. Wang , A. Hussain , S. Li , X. Zhang , R. G. Tuguntaev , X.‐J. Liang , W. Guo , F. Cao , Acta Pharm. Sin. B 2022, 12, 2014. 35847489 10.1016/j.apsb.2021.12.020 PMC9279717

[225] G. Wang , Y. Zhu , K. Li , B. Liao , F. Wang , L. Shao , L. Huang , D. Bai , J. Cardiovasc. Pharmacol. 2021, 79, 390. 10.1097/FJC.0000000000001069 PMC8340951 34091481

[226] Y. Ma , Y. Ma , M. Gao , Z. Han , W. Jiang , Y. Gu , Y. Liu , Adv. Sci. 2021, 8, 2004128. 10.1002/advs.202004128 PMC8061396 33898191

[227] X. Zhang , J. Liu , X. Yang , G. He , B. Li , J. Qin , P. R. Shearing , D. J. L. Brett , J. Hu , X. Lu , Nanoscale 2019, 11, 9733. 31066405 10.1039/c9nr00772e

[228] X. Wang , X. Wu , J. Qin , K. Ye , F. Lai , B. Li , G. He , X. Lu , D. J. L. Brett , I. P. Parkin , ACS Appl. Mater. Interfaces 2019, 11, 41009. 31599564 10.1021/acsami.9b12258

[229] X. Peng , J. Liu , C. Ming , B. Li , Z. Zhao , K. Ye , M. Zeng , R. Zou , X. Lu , J. Hu , Nanoscale 2020, 12, 11288. 32420577 10.1039/d0nr01587c

[230] R. Lu , W. Wang , B. Dong , C. Xu , B. Li , Y. Sun , J. Liu , B. Hong , Molecules 2022, 27, 8134. 36500227 10.3390/molecules27238134 PMC9737671

[231] a) J. Ouyang , X. Ji , X. Zhang , C. Feng , Z. Tang , N. Kong , A. Xie , J. Wang , X. Sui , L. Deng , Y. Liu , J. S. Kim , Y. Cao , W. Tao , Proc. Natl. Acad. Sci. USA 2020, 117, 28667; 33139557 10.1073/pnas.2016268117 PMC7682336

[232] T. Dai , W. He , S. Tu , J. Han , B. Yuan , C. Yao , W. Ren , A. Wu , Bioact. Mater. 2022, 17, 18. 35386468 10.1016/j.bioactmat.2022.01.013 PMC8958315

[233] J. Ouyang , Z. Tang , N. Farokhzad , N. Kong , N. Y. Kim , C. Feng , S. Blake , Y. Xiao , C. Liu , T. Xie , W. Tao , Nano Today 2020, 35, 100949.

[234] Z. Li , X. Sun , S. Guo , L. Wang , T. Wang , C. Peng , W. Wang , Z. Tian , R. Zhao , W. Cao , Y. Tian , Thromb. Haemostasis 2015, 114, 793. 26179778 10.1160/TH14-12-1030

[235] a) H. Wang , Y. Yang , X. Sun , F. Tian , S. Guo , W. Wang , Z. Tian , H. Jin , Z. Zhang , Y. Tian , Theranostics 2018, 8, 4969; 30429880 10.7150/thno.26193 PMC6217053

[236] J. Yao , W. Gao , Y. Wang , L. Wang , K. Diabakte , J. Li , J. Yang , Y. Jiang , Y. Liu , S. Guo , X. Zhao , Z. Cao , X. Chen , Q. Li , H. Zhang , W. Wang , Z. Tian , B. Li , F. Tian , G. Wu , S. Pourteymour , X. Huang , F. Tan , X. Cao , Z. Yang , K. Li , Y. Zhang , Y. Li , Z. Zhang , H. Jin , et al., JACC: Basic Transl. Sci. 2020, 5, 53. 32043020 10.1016/j.jacbts.2019.10.007 PMC7000870

[237] B. Li , J. Gong , S. Sheng , M. Lu , S. Guo , J. Yao , H. Zhang , X. Zhao , Z. Cao , X. Sun , H. Wang , Y. Cao , Y. Jiang , Z. Tian , B. Liu , H. Zhao , Z. Zhang , H. Jin , Y. Tian , Bioeng. Transl. Med. 2021, 6, e10193. 33532592 10.1002/btm2.10193 PMC7823128

[238] Y. Jiang , J. Kou , X. Han , X. Li , Z. Zhong , Z. Liu , Y. Zheng , Y. Tian , L. Yang , Oxid. Med. Cell. Longevity 2017, 2017, 8519169. 10.1155/2017/8519169 PMC5278230 28191279

[239] F. Wang , Q. Gao , S. Guo , J. Cheng , X. Sun , Q. Li , T. Wang , Z. Zhang , W. Cao , Y. Tian , Biomed. Res. Int. 2013, 2013, 737264. 23509769 10.1155/2013/737264 PMC3591177

[240] L. Jiang , J. Wang , J. Jiang , C. Zhang , M. Zhao , Z. Chen , N. Wang , D. Hu , X. Liu , H. Peng , M. Lian , Eur. J. Pharm. Biopharm. 2020, 146, 101. 31841689 10.1016/j.ejpb.2019.12.005

[241] Z. Cao , G. Yuan , L. Zeng , L. Bai , X. Liu , M. Wu , R. Sun , Z. Chen , Y. Jiang , Q. Gao , Y. Chen , Y. Zhang , Y. Pan , J. Wang , ACS Nano 2022, 16, 10608. 35759554 10.1021/acsnano.2c02177

[242] a) O. Bornachea , A. Benitez‐Amaro , A. Vea , L. Nasarre , D. de Gonzalo‐Calvo , J. C. Escola‐Gil , L. Cedo , A. Iborra , L. Martínez‐Martínez , C. Juarez , J. A. Camara , C. Espinet , M. Borrell‐Pages , L. Badimon , J. Castell , V. Llorente‐Cortés , Theranostics 2020, 10, 3263; 32194867 10.7150/thno.37305 PMC7053206

[243] M. Lameijer , T. Binderup , M. M. T. van Leent , M. L. Senders , F. Fay , J. Malkus , B. L. Sanchez‐Gaytan , A. J. P. Teunissen , N. Karakatsanis , P. Robson , X. Zhou , Y. Ye , G. Wojtkiewicz , J. Tang , T. T. P. Seijkens , J. Kroon , E. S. G. Stroes , A. Kjaer , J. Ochando , T. Reiner , C. Pérez‐Medina , C. Calcagno , E. A. Fisher , B. Zhang , R. E. Temel , F. K. Swirski , M. Nahrendorf , Z. A. Fayad , E. Lutgens , W. J. M. Mulder , et al., Nat. Biomed. Eng. 2018, 2, 279. 30936448 10.1038/s41551-018-0221-2 PMC6447057

[244] Y. Zhou , S. Y. Wang , X. Y. Liang , Z. Heger , M. Xu , Q. Lu , M. Yu , V. Adam , N. Li , ACS Nano 2022, 16, 10517. 35762565 10.1021/acsnano.2c01778

[245] T. Binderup , R. Duivenvoorden , F. Fay , M. M. T. van Leent , J. Malkus , S. Baxter , S. Ishino , Y. Zhao , B. Sanchez‐Gaytan , A. J. P. Teunissen , Y. C. A. Frederico , J. Tang , G. Carlucci , S. Lyashchenko , C. Calcagno , N. Karakatsanis , G. Soultanidis , M. L. Senders , P. M. Robson , V. Mani , S. Ramachandran , M. E. Lobatto , B. A. Hutten , J. F. Granada , T. Reiner , F. K. Swirski , M. Nahrendorf , A. Kjaer , E. A. Fisher , Z. A. Fayad , et al., Sci. Transl. Med. 2019, 11, eaaw7736. 31434756 10.1126/scitranslmed.aaw7736 PMC7328283

[246] a) T. Y. Wang , X. Y. Zhu , F. G. Wu , Bioact. Mater. 2023, 23, 129; 36406249 10.1016/j.bioactmat.2022.10.008 PMC9661653

[247] J. S. Rink , W. Sun , S. Misener , J.‐J. Wang , Z. J. Zhang , M. R. Kibbe , V. P. Dravid , S. Venkatraman , C. S. Thaxton , ACS Appl. Mater. Interfaces 2018, 10, 6904. 29385802 10.1021/acsami.7b18525 PMC8495904

[248] A. M. Garzón‐Porras , D. L. Bertuzzi , K. Lucas , L. C. E. da Silva , M. G. de Oliveira , C. Ornelas , ACS Appl. Polym. Mater. 2020, 2, 2027.

[249] a) A. Wijaya , Y. Wang , D. Tang , Y. Zhong , B. Y. Liu , M. Yan , Q. H. Jiu , W. Wu , G. X. Wang , J. Mater. Chem. B 2022, 10, 607; 34994373 10.1039/d1tb01455b

[250] Z. Wu , M. Zhou , X. Tang , J. Zeng , Y. Li , Y. Sun , J. Huang , L. Chen , M. Wan , C. Mao , ACS Nano 2022, 16, 3808. 35199998 10.1021/acsnano.1c08391

[251] M. Xu , Y. Zhou , C. Ren , X. Liang , N. Li , Adv. Funct. Mater. 2021, 31, 2104892.

[252] R. Hu , C. Dai , C. Dong , L. Ding , H. Huang , Y. Chen , B. Zhang , ACS Nano 2022, 16, 15959. 36219731 10.1021/acsnano.2c03422

[253] M. E. Lobatto , Z. A. Fayad , S. Silvera , E. Vucic , C. Calcagno , V. Mani , S. D. Dickson , K. Nicolay , M. Banciu , R. M. Schiffelers , J. M. Metselaar , L. van Bloois , H.‐S. Wu , J. T. Fallon , J. H. Rudd , V. Fuster , E. A. Fisher , G. Storm , W. J. M. Mulder , Mol. Pharmaceutics 2010, 7, 2020. 10.1021/mp100309y PMC3345199 21028895

[254] F. M. van der Valk , D. F. van Wijk , M. E. Lobatto , H. J. Verberne , G. Storm , M. C. Willems , D. A. Legemate , A. J. Nederveen , C. Calcagno , V. Mani , S. Ramachandran , M. P. Paridaans , M. J. Otten , G. M. Dallinga‐Thie , Z. A. Fayad , M. Nieuwdorp , D. M. Schulte , J. M. Metselaar , W. J. Mulder , E. S. Stroes , Nanomedicine 2015, 11, 1039. 25791806 10.1016/j.nano.2015.02.021 PMC4625798

[255] A. A. Shiozaki , T. Senra , A. T. Morikawa , D. F. Deus , A. T. Paladino‐Filho , I. M. Pinto , R. C. Maranhão , Clinics 2016, 71, 435. 27626473 10.6061/clinics/2016(08)05 PMC4975788

[256] S. E. Nissen , T. Tsunoda , E. M. Tuzcu , P. Schoenhagen , C. J. Cooper , M. Yasin , G. M. Eaton , M. A. Lauer , W. S. Sheldon , C. L. Grines , S. Halpern , T. Crowe , J. C. Blankenship , R. Kerensky , JAMA, J. Am. Med. Assoc. 2003, 290, 2292. 10.1001/jama.290.17.2292 14600188

[257] D. G. Kallend , J. A. A. Reijers , S. E. Bellibas , A. Bobillier , H. Kempen , J. Burggraaf , M. Moerland , P. L. J. Wijngaard , Eur. Heart J.: Cardiovasc. Pharmacother. 2016, 2, 23. 27418968 10.1093/ehjcvp/pvv041 PMC4900740

[258] S. J. Nicholls , R. Puri , C. M. Ballantyne , J. W. Jukema , J. J. P. Kastelein , W. Koenig , R. S. Wright , D. Kallend , P. Wijngaard , M. Borgman , K. Wolski , S. E. Nissen , JAMA Cardiol. 2018, 3, 806. 30046837 10.1001/jamacardio.2018.2112 PMC6233637

[259] a) J.‐C. Tardif , C. M. Ballantyne , P. Barter , J.‐L. Dasseux , Z. A. Fayad , M.‐C. Guertin , J. J. P. Kastelein , C. Keyserling , H. Klepp , W. Koenig , P. L. L'Allier , J. Lespérance , T. F. Lüscher , J. F. Paolini , A. Tawakol , D. D. Waters , M. Pfeffer , V. Brown , J. Rouleau , P. Watkins , L. J. Wei , G. Gosselin , C. Chayer , S. Lanthier , G. B. Pelletier , N. Racine , H. Agarwal , E. Brilakis , L. Cannon , D. Carrié , et al., Eur. Heart J. 2014, 35, 3277; 24780501 10.1093/eurheartj/ehu171 PMC4258222

[260] J.‐C. Tardif , J. Grégoire , P. L. L'Allier , R. Ibrahim , J. Lespérance , T. M. Heinonen , S. Kouz , C. Berry , R. Basser , M.‐A. Lavoie , M.‐C. Guertin , J. Rodés‐Cabau , Effect of rHDL on Atherosclerosis‐Safety and Efficacy (ERASE) Investigators , JAMA, J. Am. Med. Assoc. 2007, 297, 1675. 10.1001/jama.297.15.jpc70004 17387133

[261] C. Michael Gibson , S. Korjian , P. Tricoci , Y. Daaboul , M. Yee , P. Jain , J. H. Alexander , P. G. Steg , A. M. Lincoff , J. J. Kastelein , R. Mehran , D. M. D'Andrea , L. I. Deckelbaum , B. Merkely , M. Zarebinski , T. O. Ophuis , R. A. Harrington , Circulation 2016, 134, 1918. 27881559 10.1161/CIRCULATIONAHA.116.025687 PMC5147036

[262] a) B. Zheng , D. Duffy , P. Tricoci , H. Kastrissios , M. Pfister , S. D. Wright , A. Gille , M. A. Tortorici , Br. J. Clin. Pharmacol. 2021, 87, 2558; 33217027 10.1111/bcp.14666 PMC8247400

[263] S. Korjian , S. H. A. Kazmi , G. Chi , A. Kalayci , J. J. Lee , U. Talib , S. D. Wright , D. Duffy , B. A. Kingwell , R. Mehran , P. M. Ridker , C. M. Gibson , Eur. Heart J.: Cardiovasc. Pharmacother. 2023, 9, 387. 36787889 10.1093/ehjcvp/pvad014 PMC10236524

[264] P. Tricoci , D. M. D'Andrea , P. A. Gurbel , Z. Yao , M. Cuchel , B. Winston , R. Schott , R. Weiss , M. A. Blazing , L. Cannon , A. Bailey , D. J. Angiolillo , A. Gille , C. L. Shear , S. D. Wright , J. H. Alexander , J. Am. Heart Assoc. 2015, 4, e002171. 26307570 10.1161/JAHA.115.002171 PMC4599471

[265] C. M. Gibson , M. Kerneis , M. K. Yee , Y. Daaboul , S. Korjian , A. P. Mehr , P. Tricoci , J. H. Alexander , J. J. P. Kastelein , R. Mehran , C. Bode , B. S. Lewis , R. Mehta , D. Duffy , J. Feaster , M. Halabi , D. J. Angiolillo , D. Duerschmied , T. O. Ophuis , B. Merkely , Am. Heart J. 2019, 208, 81. 30580130 10.1016/j.ahj.2018.11.008

[266] C. M. Gibson , S. H. A. Kazmi , S. Korjian , G. Chi , A. T. Phillips , S. M. Montazerin , D. Duffy , B. Zheng , M. Heise , C. Liss , L. I. Deckelbaum , S. D. Wright , A. Gille , J. Cardiovasc. Pharmacol. Ther. 2022, 27, 10742484221121507. 36282079 10.1177/10742484221121507

[267] M. Dastani , H. R. Rahimi , V. R. Askari , M. R. Jaafari , L. Jarahi , A. Yadollahi , V. B. Rahimi , BioFactors 2023, 49, 108. 35674733 10.1002/biof.1874

[268] A. N. Kharlamov , A. E. Tyurnina , V. S. Veselova , O. P. Kovtun , V. Y. Shur , J. L. Gabinsky , Nanoscale 2015, 7, 8003. 25864858 10.1039/c5nr01050k

[269] A. N. Kharlamov , J. A. Feinstein , J. A. Cramer , J. A. Boothroyd , E. V. Shishkina , V. Shur , Future Cardiol. 2017, 13, 345. 28644056 10.2217/fca-2017-0009

[270] A. Kharlamov , Atherosclerosis 2019, 287, E34.

[271] L. Maillard , A. Tavildari , N. Barra , J. Billé , P. Joly , P. Peycher , M. Silvestri , F. Vochelet , Arch. Cardiovasc. Dis. 2017, 110, 682. 29102364 10.1016/j.acvd.2017.04.010

[272] L. Maillard , A. de Labriolle , C. Brasselet , B. Faurie , N. Durel , F. de Poli , S. Bosle , H. Madiot , J. Berland , L. Belle , Catheter. Cardiovasc. Interventions 2021, 98, 45. 10.1002/ccd.29065 32548891

[273] R. Colleran , M. Joner , D. Cutlip , P. Urban , M. Maeng , R. Jauhar , M. Barakat , J. M. Michel , R. Mehran , A. J. Kirtane , L. Maillard , A. Kastrati , R. A. Byrne , Cardiovasc. Revasc. Med. 2022, 34, 17. 33608239 10.1016/j.carrev.2021.01.022

[274] a) Q. Ma , S. J. Wu , L. Yang , Y. H. Wei , C. Y. He , W. S. Wang , Y. X. Zhao , Z. J. Wang , S. W. Yang , D. M. Shi , Y. Y. Liu , Z. M. Zhou , J. F. Sun , Y. J. Zhou , Adv. Sci. 2022, 10, 5;

[275] B. Banik , B. Surnar , B. W. Askins , M. Banerjee , S. Dhar , ACS Appl. Mater. Interfaces 2020, 12, 6852. 31886643 10.1021/acsami.9b19036

[276] a) Y. Yang , X. Fan , L. Li , Y. Yang , A. Nuernisha , D. Xue , C. He , J. Qian , Q. Hu , H. Chen , J. Liu , W. Huang , ACS Nano 2020, 14, 2509; 32022539 10.1021/acsnano.0c00043

[277] Q. Wang , Y. Wang , S. Liu , X. Sha , X. Song , Y. Dai , M. Zhao , L. Cai , K. Xu , J. Li , J. Nanobiotechnol. 2021, 19, 222. 10.1186/s12951-021-00962-w PMC8317354 34320994

[278] W.‐L. Wan , B. Tian , Y.‐J. Lin , C. Korupalli , M.‐Y. Lu , Q. Cui , D. Wan , Y. Chang , H.‐W. Sung , Nat. Commun. 2020, 11, 534. 31988280 10.1038/s41467-020-14413-x PMC6985250

[279] G. Wu , J. Zhang , Q. Zhao , W. Zhuang , J. Ding , C. Zhang , H. Gao , D.‐W. Pang , K. Pu , H.‐Y. Xie , Angew. Chem., Int. Ed. 2020, 59, 4068. 10.1002/anie.201913700 31854064

[280] J. Hou , J. Zhou , M. Chang , G. Bao , J. Xu , M. Ye , Y. Zhong , S. Liu , J. Wang , W. Zhang , H. Ran , Z. Wang , Y. Chen , D. Guo , Bioact. Mater. 2022, 16, 120. 35386311 10.1016/j.bioactmat.2022.02.022 PMC8958425

[281] M. Ye , J. Zhou , Y. Zhong , J. Xu , J. Hou , X. Wang , Z. Wang , D. Guo , ACS Appl. Mater. Interfaces 2019, 11, 9702. 30785263 10.1021/acsami.8b18190

[282] H. Xu , P. She , Z. Zhao , B. Ma , G. Li , Y. Wang , Adv. Mater. 2023, 35, 2300439. 10.1002/adma.202300439 36828777

[283] R. J. Zotz , U. Dietz , S. Lindemann , S. Genth‐Zotz , Herz 2019, 44, 35. 30569181 10.1007/s00059-018-4777-0

[284] N. S. Vos , N. D. Fagel , G. Amoroso , J. R. Herrman , M. S. Patterson , L. H. Piers , R. J. van der Schaaf , T. Slagboom , M. A. Vink , JACC: Cardiovasc. Interventions 2019, 12, 1691. 10.1016/j.jcin.2019.04.016 31126887

[285] K. Lee , S. G. Lee , I. Jang , S. H. Park , D. Yang , I. H. Seo , S. K. Bong , D. H. An , M. K. Lee , I. K. Jung , Y. H. Jang , J. S. Kim , W. Ryu , Sci. Rep. 2018, 8, 3666. 29507314 10.1038/s41598-018-21649-7 PMC5838243

[286] a) W. C. Xu , X. Dong , J. L. Ding , J. C. Liu , J. J. Xu , Y. H. Tang , Y. P. Yi , C. Lu , W. Yang , J. S. Yang , Y. Gong , J. L. Zhou , Int. J. Nanomed. 2019, 14, 441; 10.2147/IJN.S188439 PMC6330985 30666106

[287] a) N. Bricout , F. Chai , J. Sobocinski , A. Hertault , W. Laure , A. Ung , P. Woisel , J. Lyskawa , N. Blanchemain , Mater. Sci. Eng.: C 2020, 113, 110967; 10.1016/j.msec.2020.110967 32487386

[288] a) C. H. Lee , M. J. Hsieh , K. S. Liu , C. W. Cheng , S. H. Chang , S. J. Liu , C. J. Wang , M. Y. Hsu , K. C. Hung , Y. H. Yeh , W. J. Chen , I. C. Hsieh , J. H. Juang , M. S. Wen , Int. J. Nanomed. 2018, 13, 6039; 10.2147/IJN.S166785 PMC6179723 30323591

[289] L. Huang , H. Fang , T. Zhang , B. Hu , S. Liu , F. Lv , Z. Zeng , H. Liu , W. Zhou , X. Wang , Bioact. Mater. 2023, 23, 526. 36514389 10.1016/j.bioactmat.2022.11.015 PMC9730155

[290] M. R. Moradi , E. Salahinejad , E. Sharifi , L. Tayebi , Carbohydr. Polym. 2023, 314, 120961. 37173015 10.1016/j.carbpol.2023.120961 PMC10585653

[291] D. Kersani , J. Mougin , M. Lopez , S. Degoutin , N. Tabary , F. Cazaux , L. Janus , M. Maton , F. Chai , J. Sobocinski , N. Blanchemain , B. Martel , Eur. J. Pharm. Biopharm. 2020, 150, 156. 32179100 10.1016/j.ejpb.2019.12.017

[292] A. Gossart , D. Letourneur , A. Gand , V. Regnault , M. A. Ben Mlouka , P. Cosette , E. Pauthe , V. Ollivier , J. P. Santerre , Biomaterials 2019, 217, 119306. 31271854 10.1016/j.biomaterials.2019.119306

[293] H. Mo , C. Fu , Z. Wu , P. Liu , Z. Wen , Q. Hong , Y. Cai , G. Li , RSC Adv. 2020, 10, 15346. 35495447 10.1039/c9ra10509c PMC9052309

[294] a) X. Qi , C. Pan , L. Zhang , D. Yue , ACS Appl. Mater. Interfaces 2023, 15, 3497; 36598772 10.1021/acsami.2c19782

[295] Y. Wang , H. Lan , T. Yin , X. Zhang , J. Huang , H. Fu , J. Huang , S. McGinty , H. Gao , G. Wang , Z. Wang , Mater. Sci. Eng.: C 2020, 106, 110187. 10.1016/j.msec.2019.110187 31753395

[296] a) B. Zhang , Y. Qin , Y. Wang , Biomaterials 2022, 284, 121478; 35366606 10.1016/j.biomaterials.2022.121478

[297] a) T. J. Beldman , M. L. Senders , A. Alaarg , C. Pérez‐Medina , J. Tang , Y. Zhao , F. Fay , J. Deichmöller , B. Born , E. Desclos , N. N. van der Wel , R. A. Hoebe , F. Kohen , E. Kartvelishvily , M. Neeman , T. Reiner , C. Calcagno , Z. A. Fayad , M. P. J. de Winther , E. Lutgens , W. J. M. Mulder , E. Kluza , ACS Nano 2017, 11, 5785; 28463501 10.1021/acsnano.7b01385 PMC5492212

[298] J. Qin , Z. Peng , B. Li , K. Ye , Y. Zhang , F. Yuan , X. Yang , L. Huang , J. Hu , X. Lu , Nanoscale 2015, 7, 13991. 26228112 10.1039/c5nr02521d

[299] a) W. Najahi‐Missaoui , R. D. Arnold , B. S. Cummings , Int. J. Mol. Sci. 2020, 22, 385; 33396561 10.3390/ijms22010385 PMC7794803

[300] T. Skotland , T. G. Iversen , A. Llorente , K. Sandvig , Adv. Drug Delivery Rev. 2022, 186, 114326. 10.1016/j.addr.2022.114326 35588953

[301] a) K. S. Kim , K. Suzuki , H. Cho , Y. S. Youn , Y. H. Bae , ACS Nano 2018, 12, 8893; 30088412 10.1021/acsnano.8b04315 PMC6377080

[302] Z. Zhang , J. Guan , Z. Jiang , Y. Yang , J. Liu , W. Hua , Y. Mao , C. Li , W. Lu , J. Qian , C. Zhan , Nat. Commun. 2019, 10, 3561. 31395892 10.1038/s41467-019-11593-z PMC6687821

[303] a) C. Ghimire , H. Wang , H. Li , M. Vieweger , C. Xu , P. Guo , ACS Nano 2020, 14, 13180; 32902260 10.1021/acsnano.0c04863 PMC7799665

[304] a) E. P. Stater , A. Y. Sonay , C. Hart , J. Grimm , Nat. Nanotechnol. 2021, 16, 1180; 34759355 10.1038/s41565-021-01017-9 PMC9031277

[305] M. A. Dobrovolskaia , S. E. McNeil , J. Controlled Release 2013, 172, 456. 10.1016/j.jconrel.2013.05.025 PMC5831149 23742883

[306] R. Mohammadpour , M. A. Dobrovolskaia , D. L. Cheney , K. F. Greish , H. Ghandehari , Adv. Drug Delivery Rev. 2019, 144, 112. 10.1016/j.addr.2019.07.006 PMC6745262 31295521

[307] E. B. Ehlerding , P. Grodzinski , W. Cai , C. H. Liu , ACS Nano 2018, 12, 2106. 29462554 10.1021/acsnano.7b07252 PMC5878691

